# Pathology and Genetics in a Global Cohort of Parkinsonian Disorders

**DOI:** 10.1001/jamaneurol.2026.1634

**Published:** 2026-06-08

**Authors:** Lesley Y. Wu, Tessa du Toit, Tatiana Georgiades, Eleanor J. Stafford, Kristin Levine, Zih-Hua Fang, Simona Jasaityte, Ana-Luisa Gil Martinez, Patrick Cullinane, Eduardo De Pablo-Fernandez, Cornelis Blauwendraat, Andrew B. Singleton, Sonja W. Scholz, Bryan J. Traynor, Nicholas Wood, John Hardy, Patrick Chinnery, Henry Houlden, Richard Cain, Claire Troakes, Viorica Chelban, Geidy E. Serrano, Djordje Gveric, Catriona McLean, Seth Love, Andrew King, Andrew C. Robinson, Federico Roncaroli, Claire Shepherd, Glenda Halliday, Laura Parkkinen, Christopher M. Morris, Colin Smith, Thomas G. Beach, Steve Gentleman, Thomas T. Warner, Tammaryn Lashley, Zane Jaunmuktane, Raquel Real, Huw R. Morris

**Affiliations:** 1 Department of Clinical and Movement Neurosciences, UCL Queen Square Institute of Neurology, London, United Kingdom; 2 Data Tecnica International, Washington, DC; 3 The German Center for Neurodegenerative Diseases, Tübingen, Germany; 4 Queen Square Brain Bank for Neurological Disorders, UCL Queen Square Institute of Neurology, London, United Kingdom; 5 Global Parkinson’s Genetics Program, Chevy Chase, Maryland; 6 Coalition for Aligning Science (CAS), Chevy Chase, Maryland; 7 Neurodegenerative Diseases Research Section, National Institute of Neurological Disorders and Stroke, National Institutes of Health, Bethesda, Maryland; 8 Department of Neurology, Johns Hopkins University School of Medicine, Baltimore, Maryland; 9 Neuromuscular Diseases Research Section, National Institute on Aging, National Institutes of Health, Bethesda, Maryland; 10 Aligning Science Across Parkinson’s (ASAP) Collaborative Research Network, Chevy Chase, Maryland; 11 Department of Neurodegenerative Disease, UCL Queen Square Institute of Neurology, London, United Kingdom; 12 Department of Clinical Neurosciences, University of Cambridge, Cambridge, United Kingdom; 13 University of Bristol, Bristol, Horfield, United Kingdom; 14 London Neurodegenerative Diseases Brain Bank, King’s College London, London, United Kingdom; 15 Department of Pathology, Banner Sun Health Research Institute, Sun City, Arizona; 16 Department of Brain Sciences, Faculty of Medicine, Imperial College London, London, United Kingdom; 17 Victorian Brain Bank, Parkville, Victoria, Australia; 18 Geoffrey Jefferson Brain Research Centre, Division of Neuroscience, Faculty of Biology, Medicine and Health, University of Manchester, Manchester, United Kingdom; 19 Neuroscience Research Australia, Sydney, New South Wales, Australia; 20 Department of Biomedical Science, Faculty of Health and Medicine, University of New South Wales, Sydney, New South Wales, Australia; 21 School of Medical Sciences, Faculty of Medicine and Health, University of Sydney, Sydney, New South Wales, Australia; 22 Department of Neuropathology and The Queen’s College, University of Oxford, Oxford, United Kingdom; 23 Newcastle Brain Tissue Resource, NIHR Newcastle Biomedical Research Centre, Translational and Clinical Research Institute, Newcastle University, Newcastle upon Tyne, United Kingdom; 24 Academic Department of Neuropathology, Institute of Neurological and Cardiovascular Research, University of Edinburgh, Edinburgh, United Kingdom

## Abstract

**Importance:**

Accurate diagnosis of neurodegenerative movement disorders is challenging because of a lack of in vivo biomarkers, overlapping clinical features, and a delay in the emergence of pathognomonic features.

**Objective:**

To evaluate clinicopathological correlation, diagnostic accuracy, genetic association with pathology, and ancestry-related differences in a multiancestry brain bank cohort.

**Design, Setting, and Participants:**

This was a multicenter, retrospective, autopsy-confirmed cross-sectional brain bank study on donors enrolled between 1985 and 2024. Included were donors from 11 academic brain banks in the UK, US, and Australia. Among brain donors with available genetic data from participating brain banks, included were individuals with clinical diagnoses of Parkinson disease, Parkinson disease dementia, dementia with Lewy bodies (DLB), progressive supranuclear palsy, corticobasal syndrome, multiple system atrophy, or neurologically normal controls.

**Exposures:**

Genetic variant carrier status and clinical diagnostic category.

**Main Outcomes and Measures:**

Outcomes included clinical diagnostic accuracy, Lewy body and Alzheimer disease pathology burden, survival, association with genetic variants, and genetically inferred ancestry.

**Results:**

Among 5648 brain donors with available genetic data, a total of 3353 eligible donors (mean [SD] age at death, 76.8 [10.6] years; 2072 male [61.8%]) were included. Misdiagnosis rates for movement disorders ranged approximately from 10% to 20%. Clinical diagnoses of dementia with parkinsonism (ie, Parkinson disease dementia and DLB) were more strongly associated with Lewy body pathology than Parkinson disease without dementia (odds ratio [OR], 1.96; 95% CI, 1.30-3.04; *P* = 7.2 × 10^−4^). Lewy pathology was identified in 33 of 745 of neurologically normal controls (4.4%). Alzheimer disease copathology was present in 426 of 1064 cases (40.0%) with Lewy body disease. Carriers of the *GBA1* variant exhibited greater Lewy body burden compared with noncarriers (OR, 1.94; 95% CI, 1.24-3.03; *P* = .01) or carriers of the *LRRK2* variant (OR, 7.44; 95% CI, 2.16-25.64; *P* = .01). Pathological diagnoses differed by ancestry, with South Asian donors more likely to have progressive supranuclear palsy pathology and Ashkenazi Jewish donors more likely to have Lewy body disease (χ^2^_2_ = 35.5; *P* < .001), independent of *GBA1* and *LRRK2* variant status.

**Conclusions and Relevance:**

Findings of this cross-sectional brain bank study highlight the value of integrating genetic and pathological data to improve diagnostic accuracy. The high prevalence of Alzheimer disease copathology and ancestry-associated differences in pathology point to the need for biologically informed diagnostic tools. These results suggest supporting the integration of genetically and pathologically stratified approaches, correlating pathology with in vivo biomarkers, for future therapeutic trials.

## Key Points

**Question:**

How are genetic variants and neuropathology associated with clinical features and diagnostic accuracy in movement disorders?

**Findings:**

In this multiancestry brain bank cross-sectional study including over 3000 individuals, clinical misdiagnosis was common; dementia with parkinsonism was more strongly associated with Lewy body (LB) pathology than Parkinson disease without dementia, and Alzheimer disease copathology was frequent. Genetic variation was associated with pathological differences; *GBA1* carriers had greater LB burden, whereas carriers of the *LRRK2* pathogenic variant had a lower LB burden and longer survival.

**Meaning:**

Study results suggest that integrating genetics and neuropathology may improve diagnosis and support pathology-informed therapeutic trials.

## Teaser

This cross-sectional study investigates how genetic variants and neuropathology are associated with clinical features and diagnostic accuracy in movement disorders.

## Introduction

Neurodegenerative movement disorders are a heterogeneous group of conditions characterized by progressive motor and cognitive impairment. Clinicopathological studies of Parkinson disease (PD), dementia with Lewy bodies (DLB), progressive supranuclear palsy (PSP), corticobasal degeneration (CBD), and multiple system atrophy (MSA) have led to the development of consensus clinical diagnostic criteria, usually based on a hallmark protein-based pathology.^1,2,3,4,5^ Genetic factors contribute to both monogenic and complex forms of movement disorders; however, their integration into the clinicopathological framework remains incomplete. Genetic studies are typically conducted with clinically diagnosed patients, which may overlook the impact of misdiagnosis and copathology. Importantly, genetic profiling can help highlight the diversity of pathological features relating to prototypic clinical presentations. For instance, variants in *GBA1* are associated with widespread LB pathology^6^ whereas carriers of the *LRRK2* variant can present with clinically typical PD in the absence of LB at postmortem examination.^7^ Differentiating these diseases is challenging in the early stages, as they often present overlapping clinical features, affecting the interpretation of clinical research and investigational drug studies.

Existing work integrating genetics and pathology is often restricted to small or family-based cohorts, selected by genotype, providing limited population-level insights. Movement disorders research has largely focused on individuals of European ancestry, despite growing evidence that ancestry influences disease heterogeneity, clinical presentation, and outcome, including mortality.^8^

We harmonized the genetic data and integrated with the clinical and neuropathological data from multiple brain banks, including individuals of diverse ancestries with clinically diagnosed movement disorders and neurologically normal controls, with the aim of assessing clinicopathological correlation in individuals carrying disease-associated and risk genetic variants and the frequency of clinical misdiagnosis.

## Methods

### Study Design and Participants

We included individuals with clinically diagnosed movement disorders and neurologically unaffected controls from the Defining and Diagnosing Neurodegenerative Movement Disorders Through Integrated Analysis of Genetics and Neuropathology (MD-GAP) study (eMethods in Supplement 1) and collaborating brain banks within the Global Parkinson Genetics Program (GP2). Ethical approval to coordinate the MD-GAP study was obtained from the University College London Research Ethics Committee. Each contributing brain bank obtained local ethics approval for recruitment, storage, and distribution of brain donor material with written informed consent. The MD-GAP study integrates clinical, pathological, and genetic data from autopsy-confirmed cases with neurodegenerative diseases, focusing on movement disorders. Demographic data, main clinical diagnosis, primary pathology, and copathology were provided by brain banks (eTable 1 in Supplement 1). Clinical diagnoses included PD, PDD, DLB, PSP, corticobasal syndrome (CBS), MSA, and controls. Pathological diagnoses include LB disease, PSP, MSA, CBD, and other (ie, argyrophilic grain disease, chronic traumatic encephalopathy, primary age-related tauopathy, Pick disease, tauopathy not otherwise specified, aging-related tau astrogliopathy, vascular pathology). Brains were donated between 1985 and 2024. Self-reported race and ethnicity data were not collected from the brain banks; rather, the term *ancestry* was used. Ancestry was determined genetically using Genotools as part of the results. This study followed the Strengthening the Reporting of Observational Studies in Epidemiology (STROBE) reporting guidelines.

### Diagnostic Accuracy

The diagnostic accuracy for each pathologically defined movement disorder was evaluated by comparing the primary clinical diagnosis with neuropathology. LB diseases were classified as a single pathological entity, as distinctions between PD, PDD, and DLB relate to clinical features. CBS can arise from diverse underlying pathologies; we examined clinically diagnosed CBS relative to pathologically confirmed CBD.

### Neuropathological Assessment and Genetic Characterization

We documented amyloid-β, neurofibrillary tangle (NFT) phosphorylated tau, and α-synuclein pathology in PD, PDD, and DLB using established staging systems McKeith staging system,^9^ the unified staging system for LB disease (USSLB),^10^ Braak LB ^11^ and NFT stages,^12^ Consortium to Establish a Registry for Alzheimer’s Disease (CERAD),^13^ and Thal phases^14^ to compare pathological distributions between cases with LB disease with and without dementia. For statistical power, LB staging systems were collapsed into neocortical, limbic, and brainstem LB disease subtypes (eTable 2 in Supplement 1).

All cases were genotyped using the NeuroBooster array (Illumina) and/or underwent genome sequencing in GP2 (eTables 13 and 14 in Supplement 1).^15,16,17^ We used the PanelAPP neurodegenerative disease panel (eTables 3 and 4 in Supplement 1) to define genes of interest for neurodegenerative disease. Variants were extracted using BCFtools and annotated with ANNOVAR and defined by ClinVar as pathogenic or likely pathogenic variants.

Genetic ancestry was determined using Genotools.^18^ Clinical sex was confirmed using genetic sex inferred from genotyping data (eMethods and eTables 13 and 14 in Supplement 1).

### Statistical Analysis

Demographic comparisons between each diagnostic and ancestry group with more than 10 individuals were performed using the Kruskal-Wallis test for continuous data, followed by Bonferroni-corrected pairwise comparisons.

The accuracy of clinical diagnosis and concordance with pathological findings were evaluated with sensitivity, specificity, positive predictive value (PPV), and negative predictive value (NPV). We classified *GBA1* variants as either PD risk or Gaucher disease causing^19^ and compared their frequencies between cases and controls with Fisher exact test. We applied proportional odds logistic regression, adjusted for sex, disease duration, and age at death (AAD), with false discovery rate (FDR) correction, to assess associations between *GBA1* and *LRRK2* mutation status with DLB subtypes and Braak NFT stages, as well as the association between *APOE e4* dosage (0-2) and DLB subtypes. We evaluated the association between disease duration and *GBA1* and *LRRK2* genetic status using a Cox model, with age at onset as covariate. We compared *MAPT* haplotype distribution across pathological diagnostic groups using the χ^2^ test with FDR-corrected pairwise comparisons. We compared genetic ancestry and pathological diagnosis using Pearson χ^2^ test.

All *P* values were 2-sided, and *P* < .05 was considered statistically significant. Data analysis was performed from April to October 2025 using R statistical software, version 4.3.1 (R Project for Statistical Computing) (eTable 12 in Supplement 1).

## Results

We identified 3353 of 5648 donors (mean [SD] age at death, 76.8 [10.6] years; 1281 female [38.2%]; 2072 male [61.8%]) with antemortem primary clinical diagnoses of a movement disorder or controls: 1171 PD, 399 PDD, 227 DLB, 811 Parkinson-plus syndromes (PPS; 491 PSP, 244 MSA, 76 CBS) and 745 neurologically normal controls (Table 1).

**Table 1.** Demographic Features and Underlying Primary Pathology in 3403 Patients With Clinically Diagnosed Movement Disorder and Neurologically Healthy Controls

Clinical diagnosis	No. (%)							
	PD	PDD	DLB	PSP	MSA	CBS	Control	All
No.	1171	399	227	491	244	76	745	3353
Sex, female	470 (40.1)	115 (28.8)^a^	47 (20.1)^a^	170 (34.6)^b^	103 (42.2)^b^^,^^c^	42 (55.2)^b^^,^^c^^,^^d^	334 (44.8)^b^^,^^c^^,^^d^	1281 (38.2)
AAO, mean (SD), y	63.6 (11.8)	63.0 (10.6)	72.1 (8.5)^a^^,^^c^	66.8 (8.3)^a^^,^^b^^,^^c^	58.1 (9.5)^a^^,^^b^^,^^c^^,^^d^	64.8 (8.3)^b^	NA	NA
Disease duration, mean (SD), y	14.9 (8.2)	15.1 (8.0)	6.8 (3.6)^a^^,^^c^	7.8 (4.0)^a^^,^^b^^,^^c^	8.9 (3.8)^a^^,^^b^^,^^c^^,^^d^	8.1 (3.6)^a^^,^^c^	NA	NA
Age at death, mean (SD)	78.7 (7.9)	77.9 (6.7)	78.6 (7.6)	74.9 (7.7)^a^^,^^b^^,^^c^	67.4 (9.0)^a^^,^^b^^,^^c^^,^^d^^,^^e^^,^^f^	72.8 (7.9)^a^^,^^b^^,^^c^	77.4 (16.1)^a^^,^^c^^,^^d^^,^^e^	76.8 (10.6)
Primary pathology								
LBD	1059 (90.4)	381 (95.5)	214 (94.3)	35 (7.1)	41 (16.8)	6 (7.9)	33 (4.4)	1769 (52.8)
AD	14 (1.1)	4 (1.0)	7 (3.1)	2 (0.4)	1 (0.4)	12 (15.8)	10 (1.3)	50 (1.5)
PSP	36 (3.1)	8 (2.0)	2 (0.9)	430 (87.6)	16 (6.5)	27 (35.5)	3 (0.4)	522 (15.6)
MSA	29 (2.5)	0 (0.0)	1 (0.4)	11 (2.2)	183 (75.0)	4 (5.3)	0 (0.0)	228 (6.8)
CBD	2 (0.2)	0 (0.0)	0 (0.0)	4 (0.8)	0 (0.0)	18 (23.7)	1 (0.1)	25 (0.7)
Control	8 (0.7)	1 (0.2)	2 (0.9)	0 (0.0)	1 (0.4)	0 (0.0)	683 (91.7)	695 (20.7)
Other^g^	23 (2.0)	5 (1.3)	1 (0.4)	9 (1.8)	2 (0.8)	9 (11.8)	15 (2.0)	64 (1.9)

Abbreviations: AAO, age at onset; CBD, corticobasal degeneration; CBS, corticobasal syndrome; DLB, dementia with Lewy bodies; PSP, progressive supranuclear palsy; MSA, multiple system atrophy; PD, Parkinson disease; PDD, Parkinson disease dementia.

^a^ *P* < .05 when compared with PD.

^b^ *P* < .05 when compared with DLB.

^c^ *P* < .05 when compared with PDD.

^d^ *P* < .05 when compared with PSP.

^e^ *P* < .05 when compared with CBS.

^f^ *P* < .05 when compared with control.

^g^ Other includes argyrophilic grain disease, chronic traumatic encephalopathy, primary age-related tauopathy, Pick disease, tauopathy not otherwise specified, aging-related tau astrogliopathy, and vascular pathology.

### Demographics

Age at onset differed significantly across clinical diagnostic groups. Cases with DLB had a later disease onset than all other groups but a shorter disease duration (mean [SD], 6.8 [3.6] years) compared with PD (mean [SD], 14.9 [8.2] years) and PDD (15.1 [8.0] years), suggesting a more aggressive disease course (*P* < 2 × 10^−16^) (Table 1). Cases with MSA had a significantly earlier onset and died at a younger age (mean [SD] age, 67.4 [9.0] years) compared with other groups (*P* = 2.2 × 10^−16^). Overall, PPS were associated with shorter disease duration (mean [SD], PSP, 7.8 [4.0] years; MSA, 8.9 [3.8] years; CBS, 8.1 [3.6] years) compared with PD (mean [SD], 14.9 [8.2] years) and PDD (15.1 [8.0] years; *P* < 2 × 10^−16^). Individuals of South Asian ancestry died at a significantly younger age (mean [SD] age, 71.1 [4.9] years) than individuals of European (mean [SD] age, 77.2 [10.2] years) or Ashkenazi Jewish ancestry (mean [SD] age, 79.9 [9.1] years; *P* < .001) (eTable 5 in Supplement 1).

### Diagnostic Accuracy Across Movement Disorders

Study results suggest a high rate of clinical misdiagnosis when compared with the pathological criterion standard,^20^ with PPV varying across disease groups (Table 2). Misdiagnosis rates for movement disorders ranged approximately from 10% to 20%. A clinical diagnosis of PD, PDD, and DLB was associated with underlying LB pathology (PPV, 92.0%; 95% CI, 90.7%-93.2%). The presence of dementia significantly increased diagnostic accuracy: PDD and DLB were nearly twice as likely to be associated with LB disease pathology compared with PD (odds ratio [OR], 1.96; 95% CI, 1.30-3.04; *P = *7.2 × 10^−4^). Conversely, PD without dementia showed lower diagnostic accuracy (PPV, 90.4%). CBS exhibited the lowest PPV at 23.7%, reflecting the heterogeneous nature of the disease and the challenge of accurately predicting CBD pathology. MSA and CBS both had high specificity (98.1% and 98.3%, respectively), whereas specificity for clinically diagnosed PD, PDD, and DLB was the lowest (91.9%), indicating misdiagnosis of other movement disorders as PD, PDD, and DLB in almost 8.0% of cases.

**Table 2.** Diagnostic Accuracy Across Clinical Movement Disorder Diagnoses Compared With Neuropathological Confirmation

Disease group	%			
	Sensitivity	Specificity	PPV	NPV
PD, PDD, or DLB	92.5	91.9	92.0	91.8
PSP	80.8	97.9	87.5	96.5
MSA	79.2	98.1	75.0	98.5
CBS-CBD	72.0	98.3	23.7	99.7
Control	97.9	97.8	91.7	99.5

Abbreviations: CBD, corticobasal degeneration; CBS, corticobasal syndrome; DLB, dementia with Lewy bodies; PSP, progressive supranuclear palsy; MSA, multiple system atrophy; NPV, negative predictive value; PD, Parkinson disease; PDD, Parkinson disease dementia; PPV, positive predictive value.

There was overlap between PSP and PD as follows: 36 of 1171 patients (3.1%) clinically diagnosed with PD had primary PSP pathology, and 35 of 491 patients (7.1%) clinically diagnosed with PSP had primary LB disease pathology. Among cases clinically diagnosed with MSA, 41 of 244 (16.8%) had primary LB pathology, and 16 of 244 (6.5%) had PSP pathology. Cases with clinically diagnosed CBS were often due to PSP pathology (27 of 76 [35.5%]) or Alzheimer disease (AD) pathology (12 of 76 [15.8%]) at autopsy. Background neurodegenerative pathology was identified in 62 of 745 clinical controls (8.3%), including 80 of 745 individuals (10.7%) who died before age 65 years. LB pathology was present in 33 of 745 clinical controls (4.4%), including neocortical involvement in 10 of 33 individuals (30.3%), of whom 2 of 10 (20.0%) carried an *APOE* ε4 allele (eTable 6 in Supplement 1).

Copathology was present in 1102 of 1312 clinically affected cases (84.0%) with available data. AD pathology was most frequent, present in 426 of 1064 cases (40.0%) with LB disease (eTable 7 in Supplement 1) and associated with more extensive LB distribution (OR, 2.61; 95% CI, 1.79-3.80; *P* = 5.8 × 10^−7^). AD copathology was less common in PSP and CBD (35 of 216 [16.2%] and 2 of 12 [16.7%], respectively), which more often had additional tau pathology, such as primary age-related tauopathy or argyrophilic grain disease, or other copathologies, such as cerebral amyloid angiopathy or limbic-predominant age-related TAR DNA-binding protein 43 (TDP-43) encephalopathy. Across diagnostic groups, copathologies were associated with later age at onset (β = 1.66; 95% CI, 0.99-2.32; *P* = 1.1 × 10^−6^), as compared with patients reported to have no copathology.

### Dementia in LB Disease

We examined the association between dementia (defined by the clinical diagnosis of PDD and DLB) and pathological staging for LB, NFT, and amyloid-β plaques in LB disease. A stepwise increase in the severity of both LB and AD pathology was observed across the clinical spectrum. Greater burdens of LB and AD pathology were independently associated with a clinical diagnosis of dementia in individuals with PDD and DLB compared with PD without dementia. Neocortical LB pathology was present in 113 of 140 individuals (80.7%) with DLB, compared with 194 of 288 (67.4%) with PDD and 396 of 744 (53.2%) with PD (χ^2^_3_ = 45.75, *P* = 1.16 × 10^−10^). Similar gradients were observed in Braak NFT, CERAD, and Thal phase, supporting a cumulative increased burden of AD and LB disease pathology from PD to PDD to DLB (eTable 8 in Supplement 1).

### Genetic Variation

**Figure 1.**
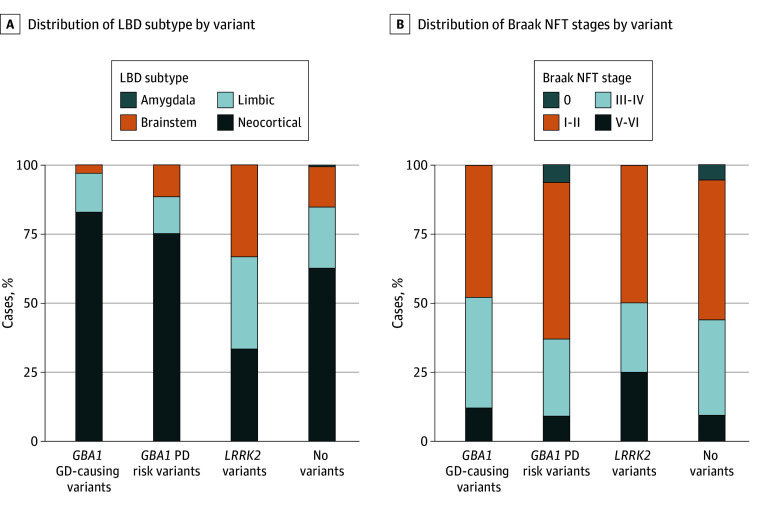
Stacked Bar Plots Showing the Distribution of Lewy Body Disease (LBD) Subtype and Braak Neurofibrillary Tangle (NFT) Stages by Variant Group in Individuals With LBD Illustrated in the figure is the proportion of cases with pathologically diagnosed LBD within each variant group stratified by LBD subtypes (A) and Braak NFT (B). Gene variant groups include *GBA1* Gaucher disease (GD)–causing variants (R159W, R170C, S235P, I299T, P305Lfs*31, T362, N409S, D448H, L483P, R502C), *GBA1* Parkinson disease (PD) risk variants (E365K, T408M), *LRRK2* variants (G2019S and Y1699C), and cases without pathogenic variants. A, Individuals carrying *GBA1* variants, particularly GD-causing variants, are more likely to have a higher LBD pathology burden. B, Indicates a relatively even distribution of Braak NFT stages across groups, with a trend toward higher NFT stages in *LRRK2* carriers.

We assessed the frequency of individuals carrying common and rare variants in genes previously associated with movement disorders (Table 3). Individuals with primary LB pathology were more likely than pathological controls to carry *GBA1* GD-causing variants (OR, 5.65; 95% CI, 1.35-23.66; *P* = 5 × 10^−3^) and *GBA1* PD risk variants (OR, 1.58; 95% CI, 1.07-2.34; *P* = .02) (eTable 9 in Supplement 1). No significant differences in *GBA1* variant frequency were observed in patients with primary PSP, MSA, or CBD pathology compared with controls. Cases with primary LB pathology carrying *GBA1* variants (regardless of clinical diagnosis) exhibited significantly more widespread LB pathology compared with cases with primary LB pathology without any known gene variants (OR, 1.94; 95% CI, 1.24-3.03; *P* = .01), or with an *LRRK2* variant (OR, 7.44; 95% CI, 2.16-25.64; *P* = .01) after adjusting for disease duration (Figure 1).

**Table 3.** Genetic Variation Across Pathologically Defined Diagnostic Groups

Diagnostic group	No. (%)							
	LBD	PSP	CBD	MSA	Other^a^	All cases	Control	Total^b^
Total, No.	1759	531	25	228	113	2656	697	3353
Sex, female	610 (34.7)	191 (36.0)	12 (48.0)	110 (48.2)	50 (44.3)	971 (36.6)	308 (44.2)	1281 (38.1)
WGS, No.	1157	473	20	183	76	1909	387	2296
*MAPT*								
H1/H1	1045 (59.4)^c^	443 (83.4)	18 (72.0)	110 (48.2)^c^^,^^d^	60 (53.1)^c^	1676 (63.1)	377 (54.1)^c^^,^^d^	2053 (61.2)
H1/H2	490 (27.9)	44 (8.3)	3 (12.0)	80 (35.1)	37 (32.7)	654 (24.6)	229 (32.9)	883 (26.3)
H2/H2	55 (3.1)	3 (0.6)	1 (4.0)	14 (6.1)	5 (4.4)	78 (2.9)	31 (4.4)	109 (3.3)
Missing	169 (9.6)	41 (7.7)	3 (12.0)	24 (10.6)	11 (9.7)	248 (9.3)	60 (8.6)	308 (9.2)
*APOE e4*								
0	1059 (60.2)	370 (69.7)	18 (72.0)	161 (70.6)	57 (50.4)	1665 (62.7)	492 (70.5)	2157 (64.3)
1	445 (25.3)	102 (19.2)	1 (4.0)	38 (16.7)	33 (29.2)	619 (23.3)	121 (17.4)	740 (22.1)
2	51 (2.9)	9 (1.7)^d^^,^^e^	1 (4.0)	1 (0.4)^d^^,^^e^	3 (2.7)	65 (2.5)	6 (0.9)^d^^,^^e^	71 (2.1)
Missing	204 (11.6)	50 (9.4)	5 (20.0)	28 (12.3)	20 (17.7)	307 (11.5)	78 (11.2)	385 (11.5)
*GBA1*								
D448H	3 (0.2)	NA	NA	NA	NA	3 (0.1)	NA	3 (0.1)
E365K	98 (5.6)	16 (3.0)	NA	6 (2.6)	2 (1.8)	122 (4.6)	21 (3.0)	143 (4.3)
I299T	1 (0.1)	NA	NA	NA	NA	1 (0.04)	NA	1 (0.03)
L483P^d^	6 (0.5)	NA	NA	NA	NA	6 (0.3)	NA	6 (0.3)
N409S	17 (1.0)	1 (0.2)	NA	1 (0.4)	NA	19 (0.7)	3 (0.4)	21 (0.6)
P305Lfs*31^f^	3 (0.3)	NA	NA	NA	NA	3 (0.2)	NA	3 (0.1)
R159W^f^	2 (0.2)	NA	NA	NA	NA	2 (0.1)	NA	2 (0.1)
R170C	1 (0.1)	1 (0.2)	NA	NA	NA	1 (0.04)	NA	3 (0.1)
R502C	5 (0.3)	NA	NA	NA	NA	5 (0.2)	NA	5 (0.2)
S235P^f^	1 (0.1)	NA	NA	NA	NA	1 (0.1)	NA	1 (0.04)
T362I	1 (0.1)	NA	NA	NA	NA	1 (0.04)	NA	1 (0.03)
T408M	33 (1.9)	11 (2.1)	1 (4.0)	3 (1.3)	3 (2.7)	51 (1.9)	12 (1.7)	63 (1.9)
c.115 + 1G>A	1 (0.1)	NA	NA	NA	NA	1 (0.04)	NA	1 (0.03)
*LRRK2*								
G2019S	14 (0.8)	2 (0.4)	NA	NA	1 (0.9)	17 (0.6)	1 (0.1)	18 (0.5)
Y1699C^f^	NA	NA	NA	NA	1 (1.3)	1 (0.1)	NA	1 (0.04)
*PRKN*								
R275W	1 (0.1)	NA	NA	NA	NA	1 (0.04)	NA	1 (0.03)
*SNCA*								
G51D	2 (0.1)	NA	NA	NA	NA	2 (0.1)	NA	2 (0.1)
*TBK1*								
E703X^f^	NA	NA	NA	NA	1 (1.3)	1 (0.1)	NA	1 (0.04)

Abbreviations: CBD, corticobasal degeneration; H1, haplotype 1; H2, haplotype 2; LBD, Lewy body disease; MSA, multiple system atrophy; NA, not applicable; PSP, progressive supranuclear palsy; WGS, whole-genome sequence.

^a^ Other includes Alzheimer disease, argyrophilic grain disease, cerebral amyloid angiopathy, cerebral vascular disease, chronic traumatic encephalopathy, frontotemporal lobar degeneration, primary age-related tauopathy, Pick disease, small vessels disease, tauopathy, and tumor.

^b^ Total includes number of cases with genetic data, either genotyped or sequenced.

^c^ *P* < .001 compared with PSP.

^d^ *P* < .05 compared with LBD.

^e^ *P* < .001 compared with other.

^f^ Frequencies have been calculated based on WGS No.

**Figure 2.**
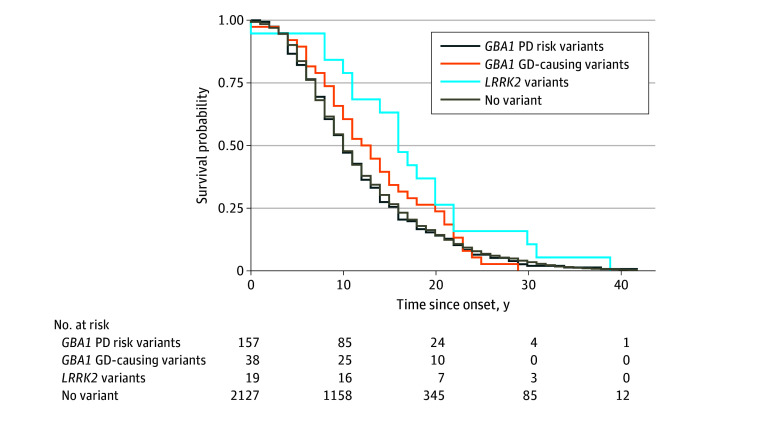
Kaplan-Meier (KM) Survival Curve in Individuals With *GBA1* and *LRRK2* Variants and Those Without Known Variants KM survival curves showing disease duration (years from symptom onset to death) stratified by gene variant group. Variant groups include *GBA1* Gaucher disease (GD)–causing variants (R159W, R170C, S235P, I299T, P305Lfs*31, T362, N409S, D448H, L483P, R502C), *GBA1* Parkinson disease (PD) risk variants (E365K, T408M), *LRRK2* variants (G2019S and Y1699C), and cases without pathogenic variants. *LRRK2* carriers had a significantly longer disease duration compared with individuals without variants (hazard ratio [HR], 0.60; 95% CI, 0.38-0.95; *P* = .02) or those carrying *GBA1* PD risk variants (HR, 0.58; 95% CI, 0.36-0.94; *P* = .03).

We identified 19 individuals with clinically diagnosed movement disorders who carried *LRRK2* gene variants (eTable 10 in Supplement 1). Of these, 18 had the p.G2019S variant, and 1 carried the p.Y1699C variant. Ashkenazi Jewish ancestry was present in 4 of 19 *LRRK2* cases (21.0%) vs 59 of 3384 cases (1.7%) in the remainder of the cohort, indicating significant enrichment of *LRRK2* among individuals of Ashkenazi Jewish descent. At a pathological level, LB pathology was found in 13 of 19 individuals (68.4%), whereas the remaining cases exhibited PSP, frontotemporal lobar degeneration (FTLD), other, or no pathology. Carriers of the *LRRK2* pathogenic variant were less likely to exhibit advanced stages of LB pathology compared with individuals without variants (OR, 0.26; 95% CI, 0.08-0.84; *P* = .05). Conversely, these individuals showed a trend toward higher Braak NFT stages; however, this was not statistically significant (Figure 1), and NFT stage distributions were similar to age-matched controls. In the survival analysis (Figure 2), carriers of the *LRRK2* variant showed a significantly reduced hazard, indicating longer survival compared with individuals without variants (hazard ratio [HR], 0.60; 95% CI, 0.38-0.95; *P* = .02) and those carrying *GBA1* PD risk variants (HR, 0.58; 95% CI, 0.36-0.94; *P* = .03). In contrast, carriers of *GBA1* variants (GD causing or PD risk and combined) did not differ significantly from the reference group of patients with LB disease without *GBA1* variants (HR, 0.99; 95% CI, 0.85-1.15; *P* = .88).

In addition to *GBA1* and *LRRK2*, pathogenic variants were identified in *SNCA*,* PRKN*, and *TBK1* genes. The pathogenic *TBK1* variant was identified in a case of clinical CBS with primary progressive aphasia, with FTLD with TDP-43 type A neuropathology, as previously reported.^21^

*MAPT* haplotype frequencies differed significantly across pathological diagnostic groups. Haplotype 1/haplotype 1 (H1/H1) is most frequent in PSP, with significantly higher proportions compared to all other groups except CBD, and the least frequent in MSA (χ^2^_5_ = 160; *P* = 8.2 × 10 ^−33^) (Table 3).

*APOE ε*4 dosage differed significantly between groups (χ^2^_5_ = 54.5; *P* = 1.6 × ^−10^). Cases with primary LB pathology had the highest burden of ε4 dosage compared with MSA, PSP, and controls (Table 3). In cases with primary LB pathology, each additional copy of the *APOE* ε4 allele was associated with a 2-fold increase in the odds of having more widespread LB pathology (OR, 2.25; 95% CI, 1.75-2.90; *P* = 4.2 × 10^−10^), independent of age, sex, and brain bank.

### Ancestry Analysis

We found a significant association between ancestry and pathological diagnosis (χ^2^_2_ = 35.5; *P* = 1.95 × 10^−8^) (eTable 5 in Supplement 1). Primary LB pathology was more common in individuals of Ashkenazi Jewish ancestry compared with the South Asian population, whereas PSP was more frequent in the South Asian population (eFigure in Supplement 1). The association between Ashkenazi Jewish ancestry and primary LB pathology remained after removal of *LRRK2*, *GBA1* risk, and rare variant carriers.

## Discussion

We have completed a large multicenter autopsy-confirmed analysis integrating clinical, genetic, and pathological data in neurodegenerative movement disorders. Our findings reinforce the complexity of clinicopathological correlations in PD, DLB, and PPS and highlight the need for in vivo biomarkers for identifying underlying pathology.

We assessed misdiagnosis of parkinsonian syndromes by comparing primary clinical diagnosis to postmortem diagnosis. Despite our cohort being over 30 times larger than that of Hughes et al,^22^ clinical misdiagnosis rates remained similar (10%-20%), consistent with other clinicopathological studies^20^ and longitudinal cohorts such as Cambridgeshire Parkinson’s Incidence From General Practitioner to Neurologist (CAMPAIGN).^23^ Diagnostic discordance likely reflects limitations of clinical criteria, particularly in early disease,^24,25^ and may be further influenced by copathology, underscoring the need for careful documentation of mixed pathologies in future clinicopathological studies. We showed that clinical diagnostic accuracy for primary LB pathology increases in the presence of dementia, consistent with previous research showing that symptoms such as visual hallucinations strongly support underlying LB pathology, and occur less commonly in PSP or MSA.^26^ This study suggests a high rate of misdiagnosis in MSA, where over 20% of clinically diagnosed cases had alternative pathology at autopsy, most commonly primary LB pathology or PSP. Although some misclassification may reflect limited familiarity with the clinical features of PPS, these findings underscore the inherent difficulty in differentiating movement disorders, particularly in the early stages. Recent advances in seed amplification assays (SAAs) may help define underlying pathology during life. α-Synuclein seeding activity has been detected in PPS using cerebrospinal fluid α-synuclein SAAs and may be associated with differences in clinical disease course.^27^ These findings align with our autopsy data showing LB copathology in 19% of PPS in our cohort, comparable with cerebrospinal fluid α-synuclein SAA positivity reported in clinical cohorts (10%-30%).^27,28,29^

The increasing focus on biological disease classification and in vivo biomarkers has renewed interest in grouping PD, PDD, and DLB as a unified disease entity characterized by neuronal synuclein pathology.^30,31^ Although these disorders may be indistinguishable at the individual pathological level,^32^ group-level differences are evident. Previous studies, confirmed by our cohort, have shown that dementia in PD and DLB is strongly associated with a higher burden of cortical LB pathology.^33^ Differences between PDD and DLB are also apparent, with DLB, defined by primary or early dementia, associated with a later age at onset and a higher rate of AD pathology,^34^ as observed in our cohort. The presence of multiple pathologies was associated with older age at onset, possibly reflecting an age-related decline in protein clearance mechanisms and the accumulation of pathological proteins.^35^

Large-scale genotyping and genome sequencing allow for the rapid definition of relevant common and rare genetic variations. We identified pathogenic/likely pathogenic variants in 5 genes previously implicated in neurodegenerative movement disorders. The most frequently observed mutations were in *GBA1* and *LRRK2*, consistent with their established role in parkinsonism. The frequencies of *GBA1* GD-causing, PD risk, and *LRRK2* variants in our autopsy cohort are comparable with those reported in living UK cohorts.^36,37^ Carriers of the *GBA1* variant exhibited a broader distribution of LB pathology, contrasting with smaller prior studies showing no significant differences between carriers and noncarriers.^38,39^ These discrepancies may reflect limited statistical power in earlier studies and highlight the need for large-scale genetic-pathological studies.

In primary LB pathology, carriers of the *LRRK2* variant had longer disease duration than *GBA1* carriers and noncarriers, consistent with previous reports of a milder *LRRK2*-associated disease course.^40^ All *LRRK2* carriers (n = 19) exhibited some degree of NFT pathology, including 2 carriers of p.G2019S clinically diagnosed with PD but pathologically confirmed as PSP, consistent with previous reports describing PSP-like tau pathology in carriers of *LRRK2* p.G2019S.^41,42^ Interestingly, 1 patient clinically diagnosed with *LRRK2* PD did not have pathology (ie, α-synuclein or tau) at autopsy. Although α-synuclein oligomer levels were not assessed, emerging evidence suggests *LRRK2* PD without LB pathology may involve higher levels of α-synuclein oligomers in the brain.^43,44^ These findings highlight the pleiotropic and heterogeneous pathological effects of pathogenic *LRRK2* variants.

Sample sizes for non-European ancestry groups remained limited, precluding ancestry-specific analyses. SAS ancestry was more frequently associated with PSP and Ashkenazi Jewish ancestry with primary LB pathology in this autopsy series. However, these patterns may reflect recruitment bias rather than true biological variation, as cultural factors and differences in health system capacity may influence brain bank representation, underscoring the need for expanded representation and validation studies in underrepresented populations.

### Limitations

This study has several limitations inherent to brain bank research. Referral and sampling bias may persist despite the multicenter design, reflected in a younger age at diagnosis and longer disease duration than reported in population-based cohorts. Pathological staging systems, although invaluable in understanding disease processes, provide relatively coarse measures of disease burden and may vary between pathologists and brain banks. Quantitative approach using whole scanned digital images may improve correlations between pathology and clinical phenotypes. In addition, incomplete documentation of coexisting pathologies, evolving diagnostic frameworks (eg, Aging-Related Tau Astrogliopathy [ARTAG]), variability in staging systems across centers and time periods, and the use of center-specific or broad classifications (eg, tauopathy) may have influenced prevalence estimates and interpretation. To promote transparency, we compiled the diagnostic and methodological approaches used across brain banks over time in eTable 11 in Supplement 1.

## Conclusions

To our knowledge, this cross-sectional brain bank study constitutes one of the largest genotyped and genome sequenced movement disorder cohorts integrated with clinical and neuropathological annotation. Our findings highlight the power of large-scale multimodal integration for advancing understanding of movement disorders. This dataset provides a potential resource for investigating the phenotypic impact of rare variants and variants of uncertain significance and for identifying novel genotype-phenotype associations. As digital pathology advances, brain banks should adopt more systematic data collection and prioritize inclusion of underrepresented populations to capture disease heterogeneity across ancestries and support pathology-targeted diagnostics and genotype-informed therapies.

## Notes

Supplement 1.
eMethods.
**eTable 1.** Number of Clinically Diagnosed Movement Disorder Cases and Control by Brain Bank **eTable 2.** Correspondence Between Lewy Body Pathology Staging Systems **eTable 3.** List of Genes Assessed in Short-Read Genome Sequencing (n=38) **eTable 4.** Variants Identified From Short-Read Genome Sequencing (n=17) and Additional Variants of Interest (n=21) Analyzed in Genotyped Samples **eTable 5.** Frequency of Primary Pathological Diagnosis by Genetically Determined Ancestry **eTable 6.** Distribution of Incidental Lewy body Cases by Lewy body Disease Stage **eTable 7.** Distribution of Secondary Pathologies by Primary Pathology Groups **eTable 8.** Distribution of Pathology Stages by Clinical Diagnosis in Lewy Body Diseases **eTable 9.** Frequency of GBA1 Variants Aggregated into Gaucher’s Disease Causing Mutations Group and Parkinson’s Disease Risk Mutations Group by Pathological Diagnosis **eTable 10.** Cases With Pathogenic LRRK2 Mutations **eTable 11.** Summary of Neuropathological Methods Used Across Contributing Brain Banks **eTable 12.** Key Resource Table **eTable 13.** Performance of NeuroBooster Array (NBA) Probes Compared With Short-Read Genome Sequencing (GS) for Neurodegenerative Movement Disorder Associated Variants **eTable 14.** Variants With NeuroBooster Array (NBA) Probes Not Detected in Short-Read Genome Sequencing (GS) **eFigure.** Proportion of LBD and PSP Diagnoses Across Ancestry Groups
eReferences.

Supplement 2. **Nonauthor Collaborators.** Global Parkinson’s Genetic Program (GP2).

Supplement 3.
Data Sharing Statement.
