# Supplementary material for: Pathology and Genetics in a Global Cohort of Parkinsonian Disorders
Source: JAMA Neurol. 2026 Jun 8:e261634. Online ahead of print. doi: 10.1001/jamaneurol.2026.1634 (PMC13247843; doi:10.1001/jamaneurol.2026.1634)
Supplement: Supplement 1. — eMethods. eTable 1. Number of Clinically Diagnosed Movement Disorder Cases and Control by Brain Bank eTable 2. Correspondence Between Lewy Body Pathology Staging Systems eTable 3. List of Genes Assessed in Short-Read Genome Sequencing (n=38) eTable 4. Variants Identified From Short-Read Genome Sequencing (n=17) and Additional Variants of Interest (n=21) Analyzed in Genotyped Samples eTable 5. Frequency of Primary Pathological Diagnosis by Genetically Determined Ancestry eTable 6. Distribution of Incidental Lewy body Cases by Lewy body Disease Stage eTable 7. Distribution of Secondary Pathologies by Primary Pathology Groups eTable 8. Distribution of Pathology Stages by Clinical Diagnosis in Lewy Body Diseases eTable 9. Frequency of GBA1 Variants Aggregated into Gaucher’s Disease Causing Mutations Group and Parkinson’s Disease Risk Mutations Group by Pathological Diagnosis eTable 10. Cases With Pathogenic LRRK2 Mutations eTable 11. Summary of Neuropathological Methods Used Across Contributing Brain Banks eTable 12. Key Resource Table eTable 13. Performance of NeuroBooster Array (NBA) Probes Compared With Short-Read Genome Sequencing (GS) for Neurodegenerative Movement Disorder Associated Variants eTable 14. Variants With NeuroBooster Array (NBA) Probes Not Detected in Short-Read Genome Sequencing (GS) eFigure. Proportion of LBD and PSP Diagnoses Across Ancestry Groups eReferences. [file jamaneurol-e261634-s001.pdf]

Supplementary Online Content

Wu LY, du Toit T, Georgiades T, et al; Global Parkinson's Genetic Program (GP2). Pathology and genetics in a global cohort of parkinsonian disorders. *JAMA Neurol*. Published online June 8, 2026. doi:10.1001/jamaneurol.2026.1634

eMethods.

eTable 1. Number of Clinically Diagnosed Movement Disorder Cases and Control by Brain Bank

eTable 2. Correspondence Between Lewy Body Pathology Staging Systems

eTable 3. List of Genes Assessed in Short-Read Genome Sequencing (n=38)

eTable 4. Variants Identified From Short-Read Genome Sequencing (n=17) and Additional Variants of Interest (n=21) Analyzed in Genotyped Samples

eTable 5. Frequency of Primary Pathological Diagnosis by Genetically Determined Ancestry

eTable 6. Distribution of Incidental Lewy body Cases by Lewy body Disease Stage

eTable 7. Distribution of Secondary Pathologies by Primary Pathology Groups

eTable 8. Distribution of Pathology Stages by Clinical Diagnosis in Lewy Body Diseases

eTable 9. Frequency of GBA1 Variants Aggregated into Gaucher's Disease Causing Mutations Group and Parkinson's Disease Risk Mutations Group by Pathological Diagnosis

eTable 10. Cases With Pathogenic LRRK2 Mutations

eTable 11. Summary of Neuropathological Methods Used Across Contributing Brain Banks

eTable 12. Key Resource Table

eTable 13. Performance of NeuroBooster Array (NBA) Probes Compared With Short-Read Genome Sequencing (GS) for Neurodegenerative Movement Disorder Associated Variants

eTable 14. Variants With NeuroBooster Array (NBA) Probes Not Detected in Short-Read Genome Sequencing (GS)

eFigure. Proportion of LBD and PSP Diagnoses Across Ancestry Groups

eReferences.

This supplementary material has been provided by the authors to give readers additional information about their work.

© 2026 Wu LY et al. *JAMA Neurology*.

eMethods.

Study design

Understanding movement disorders through the integrated analysis of Genetics and neuroPathology (MD-GAP) includes 11 brain banks (eTable 1) and is funded by the Medical Research Council UK and Global Parkinson's Genetic Program (GP2, https://gp2.org/). We obtained ethical approval to coordinate the MD-GAP study from the UCL Queen Square Institute of Neurology research ethics committee (23473/001). Each brain bank has local ethics approval for the recruitment, storage and distribution of brain material from brain donors with appropriate consent. We set up data and material sharing agreements with each brain bank in the UK and Australia. An additional contract was signed with the Michael J. Fox Foundation (MJFF), allowing all genetic data generated through this project to be available on Verily Workbench (https://workbench.verily.com/), a cloud-native platform designed to host harmonised data for biomedical research.

We recruited all available subjects from collaborating brain banks with either DNA or frozen tissue for DNA extraction, with clinically or pathologically confirmed diagnoses of movement disorders. Clinical diagnoses include Parkinson's disease (PD), Parkinson's disease dementia (PDD), dementia with Lewy bodies (DLB), progressive supranuclear palsy (PSP), corticobasal syndrome (CBS), multiple system atrophy (MSA), and neurologically healthy controls. Pathological movement disorders diagnoses include Lewy body disorders (LBD), progressive supranuclear palsy, corticobasal degeneration (CBD), multiple system atrophy and Other (Alzheimer's disease, Argyrophilic grain disease, Cerebral amyloid angiopathy, Cerebral Vascular Disease, Chronic Traumatic Encephalopathy, Frontotemporal lobar degeneration, Primary Age Related Tauopathy, Pick's Disease, Tauopathy not otherwise specified, Tumour). Cases with a pathological diagnosis of motor neuron disease or prion disease were excluded from the study.

The summary demographic, clinical, and pathological data were obtained through three sources: direct sharing by the brain banks' manager, extraction from the UK Brain Bank Network (UKBBN) database, and clinical and pathology reports for cases from London Neurodegenerative Diseases Brain Bank (King's College London) and Multiple Sclerosis and Parkinson's Tissue Bank (Imperial College London). Clinical diagnoses were assigned according to these sources, and where available, clinical records were reviewed by MD-GAP's clinical team. Pathological diagnoses were recorded as reported in the pathology reports, preserving the original order of diagnoses. In cases where a formal diagnosis was not provided but semi-quantitative staging was available, a pathological diagnosis was inferred based on the staging data. Some cases had a clinical diagnosis but no corresponding pathological findings; for example, a clinical diagnosis of PD without LBs on Braak staging was recorded as a control. Cases were only recorded as lacking co-pathologies if this was explicitly stated in the reports. To improve transparency and facilitate assessment of inter-center variability, we compiled a table (eTable 11) summarising diagnostic criteria, staging system and antibodies used by the contributing brain banks, where such information was available.

We used REDCap, a secure web-based application designed for data collection and management (https://projectredcap.org/) to store clinical and pathological data. The database includes five instruments: 1) Base and demographics, 2) Family history, 3) Clinical information, 4) Neuropathology and 5) Genetics and transcriptomics. The data fields were harmonised with data fields from the GP2 data dictionary to facilitate data integration and large-scale meta-analyses. GP2 is an ASAP-funded project aimed at genotyping over 150,000 participants and genome sequence (GS) over 10,000 individuals across the world to further our understanding of the genetic architecture of PD. GP2 includes clinical and brain bank cohorts.

DNA extraction

DNA was extracted from either the cerebellum or frontal cortex. We conducted quality control by performing Qubit fluorometry or nanodrop (260/280 ratio of ~1.8) to determine DNA concentration and diluted accordingly in 96-well plates before genotyping and sequencing.

© 2026 Wu LY et al. *JAMA Neurology*.

Genotyping

Available samples were genotyped using the Illumina Global Diversity Array chip with Neurobooster, which has a high-density SNV backbone (1.9 M SNVs) with an extra 95K custom content added, including known causal variants for various neurodegenerative diseases and imputation boosters for underrepresented populations (https://github.com/GP2code/Neuro_Booster_Array). Unlike GS, microarray genotyping focuses on specific regions. Genotyped data were called from IDAT files and processed using GenoTools (https://github.com/GP2code/GenoTools), a pipeline developed to automate the quality control (QC) process and estimation of ancestry. All data were imputed against the TOPMed r2 panel with Eagle v2.4 phasing on the TOPMed Imputation Server using Minimac4 (https://imputation.biodatacatalyst.nhlbi.nih.gov/33).

Genotype data were filtered using PLINK v2 (https://www.cog-genomics.org/plink/2.0/). Individuals and SNPs with >5% missing data were excluded using the –mind 0.05 and –geno 0.05 filters, respectively, to ensure high-quality data.

Variant selection was based on GS findings and additional important movement disorder-associated variants, shown in eTable 4. GS identified variants from this cohort (n=17) classified as pathogenic/ likely pathogenic based on Clinvar (https://www.ncbi.nlm.nih.gov/clinvar/ downloaded on 2024/09/17) were assessed in all cases that underwent NeuroBooster array (NBA). Of the 17 variants detected by GS, 13 had valid probes in the Illumina NBA chip. Additional rare variants (n = 21) of interest in *GBA1*, *LRRK2*, *PINK1*, *PRKN*, *SNCA* and *VPS35* with probes present in the NBA chip were selected. This includes all *LRRK2* and *SNCA* variants defined as pathogenic in Genereviews (https://www.ncbi.nlm.nih.gov/books/NBK1116/) and ClinVar (https://www.ncbi.nlm.nih.gov/clinvar/), *GBA1* variants (PD risk alleles and GD causing variants) that occurred in >0.1% of European PD as per Malek et al. 2018 [1], *PRKN* variants with >1% frequency in autosomal recessive PD patients as per Menon et al. 2024 [2], and the *PINK1* L347P variant described by Morales-Briceno et al. [3]. Therefore, a total of 34 variants were assessed in cases that underwent NBA.

Genome sequencing

Selected samples (n = 2954) underwent Illumina short-read Genome sequencing (GS) to examine both coding and non-coding regions, enabling detection of rare and common variants, structural variations, and mutations linked to phenotypes. Reads were aligned to the GRCh38DH reference genome, and small variants were called.

Joint genotyping was performed using the Broad Institute's pipeline for samples passing AMP-PD quality control (https://amp-pd.org). gVCFs generated with DeepVariant were merged using GLnexus (v1.6.1) with settings requiring minimum allele quality scores (min_AQ1, min_AQ2 ≥10), allowing monoallelic sites, and supporting up to 32 alternate alleles. The pipeline preserved partial data, genotype likelihoods (PL), and revised genotypes. Sample-level FORMAT fields—DP, AD, GQ, and PL—were conservatively merged (e.g., minimum DP and GQ values).

High-quality variants were retained if they passed variant quality score recalibration, had a call rate >0.95, genotype quality ≥20 and read depth ≥10. No allele balance (AB) filtering was applied at the sample level. Samples were excluded for excessive heterozygosity (F-statistic > ±0.15) or sex mismatches between clinical and genetic sex (based on the X chromosome). Relatedness checks were used to identify duplicates and contamination.

A total of 415 genes were selected based on the Genomics England PanelApp Adult Onset Neurodegenerative Disorders panel, release R.58 (v7.15, 30 October 2024) https://panelapp.genomicsengland.co.uk/panels/474/. Gene-level variant extraction was performed using bcftools (bcftools(1)) and variant-level annotation was performed with ANNOVAR (December 2023, ANNOVAR Documentation). Only genes associated with a movement disorder phenotype and a "green" (GEL status 3) classification, indicating a high level of evidence supporting the gene's role in disease, were included in the final analysis (n=36). Exceptions were made for genes of particular interest

© 2026 Wu LY et al. *JAMA Neurology*.

regardless of GEL status, including RAB39B and VPS13C, therefore, 38 genes were examined as described in eTable 3.

Variants in dominant and recessive genes were retained if coding or splicing, and classified as pathogenic/ likely pathogenic according to Clinvar (https://www.ncbi.nlm.nih.gov/clinvar/). *GBA1* variants were retained irrespective of Clinvar classification and allele frequency. Single heterozygous carriers in recessive genes were excluded from analysis, except for *GBA1*. Synonymous variants were excluded.

17 variants of interest were identified in 2954 cases, shown in eTable 4.

Quantification of NeuroBooster array probe performance

In a subset of cases (n=2214) across varied cohorts both NBA and GS data were available. We calculated the proportion of false positive and false negative genotype calls and calculated the sensitivity and specificity of each probe for variants detected by NBA, considering GS as the reference (eTable 13). When more than one probe was available for the same variant, the best probe was selected, prioritising specificity over sensitivity to limit the rate of false positives. Where probes performed equally, a single probe per variant was selected. Of the variants searched for in NBA, 21 probes were validated against GS, each demonstrating a specificity greater than 99.9%.

eTable 14 lists NBA probe names for variants not detected in GS. As these variants were not observed in GS, their corresponding probes were not validated and were not reported further.

Combining GS and validated findings genotyping, 20 unique mutations were identified in our cohort. Following collaboration with brain banks and review of relevant literature, variants in *PSAP* and *C19orf12* were excluded from the main results shown in Table 3. In contrast to the classification in PanelApp, we found no confirmatory segregation studies for *PSAP*, and reported cases have largely involved sporadic rather than familial disease [4]. Furthermore, neither of the two cases with *C19orf12* variants demonstrated brain iron accumulation on post-mortem examination, questioning its pathogenicity. Accordingly, only 18 pathogenic or likely pathogenic variants are reported in the main text.

© 2026 Wu LY et al. *JAMA Neurology*.

eTable 1. Number of clinically diagnosed movement disorder cases and control by brain bank

Brain Bank	Contacts	N
Queen Square Brain Bank	**Zane Jaunmuktane**<br>**Tammaryn Lashley**<br>**Tom Warner**	1335
Multiple Sclerosis and Parkinson's Tissue Bank	Djordje Gveric<br>**Steve Gentleman**	637
Banner Sun Health Research Institute [5]	**Thomas Beach**<br>Geidy Serrano	551
Edinburgh Brain and Tissue Bank	**Colin Smith**<br>Chris-Anne McKenzi	138
Newcastle Brain Tissue Resource	Debbie Lett<br>**Chris Morris**	113
Oxford Brain Bank	Carolyn Sloan<br>**Laura Parkkinen**	116
Sydney Brain Bank	**Glenda Halliday**<br>**Claire Shepherd**	110
Manchester Brain Bank	**Andrew Robinson**<br>**Federico Roncaroli**	110
London Neurodegenerative Diseases Brain Bank	Claire Troakes<br>**Andrew King**	101
South West Dementia Brain Bank	Candida Tasman<br>Richard Cain<br>**Seth Love**	78
Victoria Brain Bank	**Catriona McLean**	64
**Total**	-	3353

© 2026 Wu LY et al. *JAMA Neurology*.

11 brain banks have been included in the present study. UK and Australian brain banks are part of the MD-GAP study. Banner Sun Health Research Institute is part of the GP2 study only.
In the Contacts column, group Principal Investigators are in bold

© 2026 Wu LY et al. *JAMA Neurology*.

eTable 2. Correspondence between Lewy Body Pathology staging systems

Unified staging system for LBD	McKeith stage	Lewy Body Braak stage
Neocortical	Neocortical	Stages 5 - 6
Limbic	Limbic	Stage 4
Brainstem	Brainstem	Stages 1 - 3
Amygdala	Amygdala	..

A table showing equivalence between different staging systems. Based on BrainNet Europe Protocol [6].

© 2026 Wu LY et al. *JAMA Neurology*.

eTable 3. List of genes assessed in short-read Genome Sequencing (n=38)

Gene	Mode of Inheritance	Phenotypes
ATP1A3	Monoallelic	Alternating hemiplegia of childhood; CAPOS syndrome; Rapid-Onset Dystonia-Parkinsonism
AUH	Biallelic	Dystonia
C19orf12	Both Monoallelic and Biallelic	Spastic paraplegia; Neurodegeneration with brain iron accumulation
CHCHD2	Monoallelic	PD
CHMP2B	Monoallelic	FTD and/or amyotrophic lateral sclerosis; Dystonia
COASY	Biallelic	COASY protein-associated neurodegeneration; Neurodegeneration with brain iron accumulation
DNAJC6	Biallelic	EOPD
FBXO7	Biallelic	Dystonia; PD
FTL	Monoallelic	Neurodegeneration with brain iron accumulation
GBA	Biallelic	Late-onset PD
GCH1	Both Monoallelic and Biallelic	Dystonia; DOPA-responsive; with or without hyperphenylalaninemia
KIAA1161	Biallelic	Basal ganglia calcification
LRRK2	Monoallelic	PD
LYST	Biallelic	Chediak-Higashi syndrome; peripheral neuropathy; Parkinsonism; spastic paraplegia
MAPT	Monoallelic	FTD with or without parkinsonism
NAA60	Biallelic	Basal ganglia calcification
NPC1	Biallelic	Niemann-Pick disease
NPC2	Biallelic	Dystonia; Niemann-Pick disease
PANK2	Biallelic	Dystonia; Neurodegeneration with brain iron accumulation

© 2026 Wu LY et al. *JAMA Neurology*.

PARK7	Biallelic	EOPD
PDGFB	Monoallelic	Basal ganglia calcification
PDGFRB	Monoallelic	Dystonia; Basal ganglia calcification
PINK1	Biallelic	EOPD; Dystonia
**Gene**	**Mode of Inheritance**	**Phenotypes**
PLA2G6	Biallelic	PD; Neurodegeneration with brain iron accumulation
PRKN	Biallelic	PD
PSAP	Monoallelic	PD
RAB32	Monoallelic	PD
RAB39B	X-Linked: hemizygous mutation in males, monoallelic mutations in females may cause disease (may be less severe, later onset than males)	Early onset parkinsonism and intellectual disability; Waisman syndrome
SLC20A2	Monoallelic	Dystonia; Basal ganglia calcification
SNCA	Monoallelic	PD; Dementia
SPG11	Biallelic	Early onset parkinsonism; Spastic paraplegia
SYNJ1	Biallelic	PD
TBK1	Monoallelic	FTD and/or ALS; PSP-like and Cerebellar phenotypes
TREM2	Biallelic	Polycystic lipomembranous osteodysplasia with sclerosing leukoencephalopathy; Dystonia
VPS13C	Biallelic	EOPD
VPS35	Monoallelic	PD
WDR45	X-Linked: hemizygous mutation in males	Dystonia;Neurodegeneration with brain iron accumulation
XPR1	Monoallelic	Basal ganglia calcification

© 2026 Wu LY et al. *JAMA Neurology*.

Abbreviations: PD: Parkinson disease; EOPD: Early onset Parkinson disease; LOPD: Late onset Parkinson disease; FTD: Frontotemporal dementia; ALS: Amyotrophic lateral sclerosis; PSP: Progressive Supranuclear Palsy

© 2026 Wu LY et al. *JAMA Neurology*.

eTable 4. Variants identified from short-read Genome Sequencing (n=17) and additional variants of interest (n=21) analysed in genotyped samples

Gene	Hg38 Chr:pos:ref:alt	HGVS	NBA Illumina probe present (Y/N)	Variant selection
C19orf12	19:29702957:29702967:ACAGCCCCCC G:-	p.Gly58ArgfsTer10	N	Short-read GS
GBA1	1:155235843:T:C	p.Asn409Ser	Y	Short-read GS
GBA1	1:155236246:G:A	p.Thr408Met	Y	Short-read GS
GBA1	1:155236376:C:T	p.Glu365Lys	Y	Short-read GS
GBA1	1:155238192:A:G	p.Ser235Pro	Y	Short-read GS
GBA1	1:155237444:A:G	p.Ile299Thr	Y	Short-read GS
GBA1	1:155235252:A:G	p.Leu483Pro	Y	Short-read GS
GBA1	1:155237426:G:-	p.Pro305LeufsTer 31	N	Short-read GS
GBA1	1:155238630:G:A	p.Arg159Trp	N	Short-read GS
GBA1	1:155235727:C:G	p.Asp448His	Y	Short-read GS
GBA1	1:155240629:C:T	..	Y	Short-read GS
GBA1	1:155236384:G:A	p.Thr362Ile	Y	Short-read GS
LRRK2	12:40340400:G:A	p.Gly2019Ser	Y	Short-read GS
LRRK2	12:40321114:A:G	p.Tyr1699Cys	Y	Short-read GS
PRKN	6:161785820:G:A	p.Arg275Trp	Y	Short-read GS
PSAP	10:71851221:T:A	p.Met1Leu	Y	Short-read GS
TBK1	12:64498008:G:T	p.Glu703Ter	N	Short-read GS
GBA1	1:155235196:G:A	p.Arg502Cys	Y	Additional variant of interest

© 2026 Wu LY et al. *JAMA Neurology*.

GBA1	1:155238597:G:A	p.Arg170Cys	Y	Additional variant of interest
PINK1	1:20645640:T:C	p.Leu347Pro	Y	Additional variant of interest
**Gene**	**Hg38 Chr:pos:ref:alt**	**HGVS**	**NBA Illumina probe present (Y/N)**	**Variant selection**
PRKN	1:34784887:G:A	p.Arg42Pro	Y	Additional variant of interest
LRRK2	12:40299125:A:G	p.Ile1122Val	Y	Additional variant of interest
LRRK2	12:40309225:A:G	p.Asn1437Asp	Y	Additional variant of interest
LRRK2	12:40310434:C:T	p.Arg1441Cys	Y	Additional variant of interest
LRRK2	12:40310435:G:A	p.Arg1441His	Y	Additional variant of interest
LRRK2	12:40340404:T:C	p.Ile2020Thr	Y	Additional variant of interest
LRRK2	12:40363526:G:A	p.Gly2385Asp	Y	Additional variant of interest
VPS35	16:46662452:C:T	p.Asp620Asn	Y	Additional variant of interest
SNCA	4:89828149:C:T	p.Ala53Thr	Y	Additional variant of interest
SNCA	4:89828154:C:T	p.Gly51Asp	Y	Additional variant of interest
SNCA	4:89828170:C:T	p.Glu46Lys	Y	Additional variant of interest
SNCA	4:89835580:C:G	p.Ala30Pro	Y	Additional variant of interest
PRKN	6:161350205:C:T	p.Cys431Phe	Y	Additional variant of interest
PRKN	6:161350208:C:T	p.Gly430Asp	Y	Additional variant of

© 2026 Wu LY et al. *JAMA Neurology*.

				interest
PRKN	6:161360190:G:T	p.Glu395Ter	Y	Additional variant of interest
**Gene**	**Hg38 Chr:pos:ref:alt**	**HGVS**	**NBA Illumina probe present (Y/N)**	**Variant selection**
PRKN	6:161785885:C:T	p.Cys253Tyr	Y	Additional variant of interest
PRKN	6:162262715:C:CCA	p.Trp74CysfsTer8	Y	Additional variant of interest
PRKN	6:162443378:CCT:C	p.Gln34ArgfsTer5	Y	Additional variant of interest

eTable 4 contains all neurodegenerative movement disorder associated variants analysed in genotyped samples. Variants were selected from two sources: (1) ClinVar pathogenic/likely pathogenic variants identified from short-read genome sequencing (GS) (n=17; 13 with valid Illumina NBA chip probes), and (2) additional rare variants of interest (n=21) in *GBA1, LRRK2, PINK1, PRKN, SNCA,* and *VPS35.* A total of 34 variants were evaluated in NBA-genotyped cases.

eTable 5. Frequency of primary pathological diagnosis by genetically determined ancestry

	AAC/AFR	AJ	AMR	CAH	CAS	EAS	EUR	MDE	SAS
**N***	5	63	8	9	1	3	2758	2	18
**Sex, F (%)**	1 (20.0%)	25 (39.7%)	3 (37.5%)	3 (33.3%)	0 (0.0%)	2 (66.7%)	1051 (38.1%)	1 (50.0%)	8 (44.4%)
**Age at death, years(sd)**	71.2 (3.7)	79.9 (9.1)	73.8 (12.7)	73.2 (9.0)	53	72.7 (1.5)	77.2 (10.2)	81.5 (7.8)	71.1 (4.9)
**Pathology**									
LBD	3 (60.0%)	40 (63.5%)	1 (12.5%)	1 (11.1%)	0 (0.0%)	1 (33.3%)	1510 (54.7%)	1 (50.0%)	2 (11.1%)
PSP	2 (40.0%)	5 (7.9%)	1 (12.5%)	5 (55.6%)	0 (0.0%)	1 (33.3%)	433 (15.7%)	1 (50.0%)	12 (66.7%)
CBD	0 (0.0%)	0 (0.0%)	1 (12.5%)	0 (0.0%)	0 (0.0%)	1 (33.3%)	14 (0.5%)	0 (0.0%)	0 (0.0%)
MSA	0 (0.0%)	3 (4.8%)	0 (0.0%)	0 (0.0%)	1 (100.0%)	0 (0.0%)	187 (6.8%)	0 (0.0%)	2 (11.1%)

© 2026 Wu LY et al. *JAMA Neurology*.

Control	0 (0.0%)	13 (20.6%)	4 (50.0%)	2 (22.2%)	0 (0.0%)	0 (0.0%)	518 (18.8%)	0 (0.0%)	1 (5.5%)
Other	0 (0.0%)	2 (3.2%)	1 (12.5%)	1 (11.1%)	0 (0.0%)	0 (0.0%)	96 (3.5%)	0 (0.0%)	1 (5.5%)

* This table presents data for cases in which genetic ancestry was successfully inferred
AAC:African American, AFR:African, AJ:Ashkenazi Jews, AMR:Admixed American/Latin American, CAH:Complex Admixture History, CAS:Central Asian, EAS:East Asian, EUR: European, MDE: Middle Eastern, SAS: South Asian
LBD: Lewy Body Diseases,PSP: Progressive Supranuclear Palsy, MSA: Multiple System Atrophy, CBD: Corticobasal Degeneration

eTable 6. Distribution of incidental Lewy body cases by Lewy body disease stage

LBD Subtype	N	N with *APOE* e4
Amygdala	1 (3.0%)	0 (0.0%)
Brainstem	7 (21.2%)	0 (0.0%)
Limbic	7 (21.2%)	0 (0.0%)
Neocortical	10 (30.3%)	2 (40.0%)
No information available	5 (15.2%)	1 (20.0%)
*Unclassifiable	3 (9.1%)	2 (40.0%)
Total	33	5 (15.2%)

*Cases listed as “Unclassifiable” were designated as such according to the Braak LB system, indicating that the distribution of Lewy pathology did not conform to the canonical Braak staging pattern.

eTable 7. Distribution of secondary pathologies by primary pathology groups

Secondary Pathology		Primary Pathology			
		LBD	PSP	MSA	CBD
	n*	1064	216	20	12

© 2026 Wu LY et al. *JAMA Neurology*.

	**None**	209 (19.6%)	1 (0.5%)	0 (0.0%)	0 (0.0%)
	**LBD**	..	40 (18.5%)	4 (20.0%)	3 (25.0%)
	**AD**	426 (40.0%)	35 (16.2%)	4 (20.0%)	2 (16.7%)
	**PSP**	13 (1.2%)	..	0 (0.0%)	0 (0.0%)
	**MSA**	5 (0.5%)	0 (0.0%)	..	0 (0.0%)
	**CBD**	3 (0.3%)	4 (1.9%)	0 (0.0%)	..
	**Other**	408 (38.3%)	136 (63.0%)	12 (60.0%)	7 (58.3%)

* This table presents only cases for which data on secondary pathology were available.
Other tauopathies: Argyrophilic grain disease, Chronic Traumatic Encephalopathy, Primary Age Related Tauopathy, Picks, Tauopathy, Aging-related tau astrogliopathy
Other: Cerebral amyloid angiopathy, Other, Limbic-predominant age-related TDP-43 encephalopathy, Frontotemporal Lobar Degeneration, tumour

eTable 8. Distribution of pathology stages by clinical diagnosis in Lewy body diseases

		PD	PDD	DLB
	**Total**	744	288	140
	**None**	14 (1.9%)	2 (0.7%)	3 (2.1%)
	**Amygdala**	6 (0.8%)	3 (1.0%)	0 (0.0%)
	**Brainstem**	128 (17.2%)	48 (16.7%)	14 (10.0%)
	**Limbic**	200 (26.9%)	41 (14.2%)	10 (7.1%)
**McKeith Stages**	**Neocortical**	396 (53.2%)	194 (67.4%)	113 (80.7%)

© 2026 Wu LY et al. *JAMA Neurology*.

Braak LB	**Total**	582	237	80
	**0**	32 (5.4%)	7 (3.0%)	2 (2.5%)
	**1-2**	14 (2.5%)	3 (1.2%)	0 (0.0%)
	**3-4**	149 (25.6%)	27 (11.4%)	2 (2.5%)
	**5-6**	387 (66.5%)	200 (84.4%)	76 (95.0%)
Braak NFT	**Total**	778	351	178
	**0**	55 (7.1%)	18 (5.1%)	5 (2.8%)
	**I-II**	404 (51.9%)	187 (53.3%)	63 (35.4%)
	**III-IV**	258 (33.2%)	118 (33.6%)	82 (46.1%)
	**V-VI**	61 (7.8%)	28 (8.0%)	28 (15.7%)
CERAD	**Total**	703	258	123
	**No neuritic plaques**	388 (55.2%)	133 (51.6%)	25 (20.3%)
	**Sparse neuritic plaques**	119 (16.9%)	64 (24.8%)	16 (13.0%)
	**Moderate neuritic plaques**	113 (16.1%)	46 (17.8%)	28 (22.8%)
	**Frequent neuritic plaques**	83 (11.8%)	15 (5.8%)	54 (43.9%)
Thal	**Total**	615	248	121
	**0**	159 (26.0%)	77 (31.1%)	17 (14.0%)
	**1-2**	197 (32.0%)	69 (27.8%)	21 (17.4%)

© 2026 Wu LY et al. *JAMA Neurology*.

	**3**	151 (24.5%)	55 (22.2%)	50 (41.3%)
	**4-5**	108 (17.5%)	47 (18.9%)	33 (27.3%)

Percentages are calculated using cases with available data for each staging system.
PD: Parkinson's Disease, PDD: Parkinson's Disease Dementia, DLB: Dementia with Lewy Bodies

eTable 9. Frequency of *GBA1* variants aggregated into Gaucher's Disease causing mutations group and Parkinson's Disease risk mutations group by pathological diagnosis

	LBD	PSP	CBD	MSA	Other	All Cases	Control	TOTAL
WGS N	1157	473	20	183	76	1909	387	2296
GBA1 GD variants	33 (2.9%)	1 (0.2%)	0	1 (0.5%)	0	35 (1.8%)	2 (0.5%)	37 (1.6%)
GBA1 Risk variants	131 (11.3%)	27 (5.7%)	1 (5.0%)	9 (4.9%)	5 (6.6%)	173 (9.1%)	33 (8.5%)	206 (9.0%)

LBD: Lewy Body Diseases,PSP: Progressive Supranuclear Palsy, MSA: Multiple System Atrophy, CBD: Corticobasal Degeneration
Other: Alzheimer's disease, Argyrophilic grain disease, Cerebral amyloid angiopathy, Cerebral Vascular Disease, Chronic Traumatic Encephalopathy, Frontaltemporal lobar degeneration, Primary Age Related Tauopathy, Pick's Disease, Small Vessels Disease, Tauopathy, Tumour
GBA1 GD causing mutations include: D448H, I299T, L483P, N409S, P305Lfs*31, R159W, R170C, R502C, S235P, T362I. GBA1 PD risk mutations include: E365K and T408M.

eTable 10. Cases with pathogenic *LRRK2* mutations

Case	Sex	Genetic ancestry	Clinical Diagnosis	Pathological Diagnosis	Age at death	Mutation
1	M	EUR	PD	LBD	85	G2019S
2	F	AJ	PD	LBD	84	G2019S
3	F	EUR	PD	PSP	90	G2019S
4	F	EUR	PD	Control	79	G2019S
5	F	EUR	PD	LBD	73	G2019S

© 2026 Wu LY et al. *JAMA Neurology*.

6	M	EUR	PD	LBD	76	G2019S
7	F	AJ	PD	LBD	85	G2019S
8	M	EUR	PSP	FTLD	71	G2019S
9	M	CAH	PD	LBD	57	G2019S
10	F	EUR	PD	PSP	81	G2019S
11	F	EUR	PD	LBD	85	G2019S
12	F	EUR	PDD	TDP-43	87	G2019S
13	F	AJ	PD	LBD	82	G2019S
14	F	EUR	PD	Other	70	Y1699C
15	M	AJ	PD	LBD	65	G2019S
16	F	EUR	PD	LBD	81	G2019S
17	F	EUR	PD	LBD	73	G2019S
18	M	EUR	PD	LBD	70	G2019S
19	F	EUR	PD	LBD	88	G2019S

eTable 11. Summary of neuropathological methods used across contributing brain banks

© 2026 Wu LY et al. *JAMA Neurology*.

Brain Bank	Time Period	Lead pathologist	Pathological diagnosis	Diagnostic criteria	alpha-syn antibody	Tau antibody	Abeta antibody	Other antibody	Staging system	Region sampled	References	Notes
Queen Square Brain Bank	1985 - 1995		LBD		H&E						https://doi.org/10.1016/S1474-4422(05)70146-0	Brain regions sampled and consent procedure apply across al time periods. Listed regions reflect only those relevant to cases included in this analysis and are not exhaustive of overall brain bank practices
	1995 - 2005			McKeith et al. (1996)					McKeith system, Braak LB		10.1111/nan.12037	

© 2026 Wu LY et al. *JAMA Neurology*.

	2005 - 2015			McKeith et al. (2005) Braak et al, (2003)	MA1-90342; Thermo Scientific; 1:1,500				McKeith system, Braak LB		https://doi.org/10.1093/brain/awr031	
	2015 - 2025			McKeith et al. (2017), Braak et al, (2003)					McKeith system, Braak LB		https://doi.org/10.1111/nan.12648	
	1985 - 1995		PSP									
	1995 - 2005			Litvan et al (1996)								
	2005 - 2015			Litvan et al (1996)		AT8 (BioScience Life Sciences; 1:600), 3R tau (RD3; 1:2000); 4R tau (RD4; 1:200)					PMID: 12084879	Thal et al
	2015 - 2025										PMID: 22101365	NIA-AA Level of AD neuropathologicl change
	1985 - 1995		CBD								PMID: 12498954	

© 2026 Wu LY et al. *JAMA Neurology*.

	1995 - 2005			Lantos (2006)								
	2005 - 2015			Armstrong (2013)								
	2015 - 2025					AT8 clone; Thermo scientific MN1020; 1:600						
	1985 - 1995		MSA									
	1995 - 2005			Gilman et al. (1999)	MA1-90342; Thermo Scientific; 1:1,500							
	2005 - 2015											
	2015 - 2025											
	1985 - 1995		other co-pa									
	1995 - 2005											

© 2026 Wu LY et al. *JAMA Neurology*.

	2005 - 2015			(Mirra et al., 1991; Ball et al., 1997; Braak et al., 2006, Thal et al, 2002))		clone AT8, dilution 1:1200; Autogen Bioclear	clone 4G8, dilution 1:3000; Cell Sciences Inc; M0872; Dako; 1:100	microglia (CD68; Dako; PG-M1; 1:75), αB-crystallin (Novocastra; G2JF; 1:300); TDP-43 (2E2-D3, H00023435-M01, Abnova, 1:500)	CERAD, Braak NFT, Thal			
	2015 - 2025			Montine et al(. 2012)					CERAD, Braak NFT, Thal			
			Controls									
Parkinson's UK at Imperial	1985 - 1995		LBD								*Alafuzoff I, Arzberger T, Al-Sarraj S, Bodi I, Bogdanovic N, Braak H, et al. Staging of neurofibrillary pathology in Alzheimer'*	

© 2026 Wu LY et al. *JAMA Neurology*.

											s disease: a study of the BrainNet Europe Consortium. Brain Pathol 2008; 18(4): 484-96.	
	1995 - 2005										*Alafuzoff I, Ince PG, Arzberger T, Al-Sarraj S, Bell J, Bodi I, et al. Staging/typing of Lewy body related alpha-synuclein pathology: a study of the BrainNet Europe Consortium. Acta Neuropathol 2009;*	

© 2026 Wu LY et al. *JAMA Neurology*.

											117(6): 635-52.	
	2005 - 2015	ve Gentlem		McKeith et al. (2005)					McKeith, Braak, Alafuzoff		*Braak H, Del Tredici K, Rub U, de Vos RA, Jansen Steur EN, Braak E. Staging of brain pathology related to sporadic Parkinson's disease. Neurobiol Aging 2003; 24(2): 197-211.*	
	2015 - 2025	ve Gentlem		McKeith et al. (2017)	BD Transduction Clone 42				Attems		*McKeith IG, Dickson DW, Lowe J, Emre M, O'Brien JT, Feldman H, et al. Diagnosis and managem*	

© 2026 Wu LY et al. *JAMA Neurology*.

											ent of dementia with Lewy bodies: third report of the DLB Consortium. Neurology 2005; 65(12): 1863-72.	
	1985 - 1995		PSP								*Montine TJ, Phelps CH, Beach TG, Bigio EH, Cairns NJ, Dickson DW, et al. National Institute on Aging-Alzheimer's Association guidelines for the neuropathologic assessment of Alzheimer'*	

© 2026 Wu LY et al. *JAMA Neurology*.

											s disease: a practical approach. Acta Neuropathol 2012; 123(1): 1-11.	
	1995 - 2005										*Thal DR, Rub U, Orantes M, Braak H. Phases of A beta-deposition in the human brain and its relevance for the development of AD. Neurology 2002; 58(12): 1791-800.*	
	2005 - 2015			Litvan et al. (1996)					Hauw		*Attems J, Toledo JB, Walker L, Gelpi E, Gentleman S, Halliday G, et al.*	

© 2026 Wu LY et al. *JAMA Neurology*.

											Neuropathological consensus criteria for the evaluation of Lewy pathology in post-mortem brains: a multi-centre study. Acta Neuropathologica 2021; 141(2): 159-72.	
	2015 - 2025			Litvan et al. (1996)			ThermoFisher Scientific Phospho-tau Clone AT8		Roemer		*Nelson PT, Dickson DW, Trojanowski JQ, Jack CR, Boyle PA, Arfanakis K, et al. Limbic-predominant age-related*	

© 2026 Wu LY et al. *JAMA Neurology*.

											TDP-43 encephalopathy (LATE): consensus working group report. Brain 2019; 142(6): 1503-27.	
	1985 - 1995		CBD								*Trojanowski JQ, Revesz T; Neuropathology Working Group on MSA. Proposed neuropathological criteria for the post mortem diagnosis of multiple system atrophy. Neuropathol Appl Neurobiol. 2007*	

© 2026 Wu LY et al. *JAMA Neurology*.

											Dec;33(6): 615-20	
	1995 - 2005										*Dickson DW, Bergeron C, Chin SS, Duyckaerts C, Horoupian D, Ikeda K, Jellinger K, Lantos PL, Lippa CF, Mirra SS, Tabaton M, Vonsattel JP, Wakabayashi K, Litvan I; Office of Rare Diseases of the National Institutes of Health. Office of Rare Diseases*	

© 2026 Wu LY et al. *JAMA Neurology*.

											neuropathologic criteria for corticobasal degeneration.J Neuropathol Exp Neurol. 2002 Nov;61(11):935-46	
	2005 - 2015										*Hauw JJ, Daniel SE, Dickson D, Horoupian DS, Jellinger K, Lantos PL, McKee A, Tabaton M, Litvan I. Preliminary NINDS neuropathologic criteria for Steele-Richardson-Olszewski syndrome (progressi*	

© 2026 Wu LY et al. *JAMA Neurology*.

											ve supranuclear palsy). Neurology. 1994 Nov;44(11):2015-9	
	2015 - 2025						ThermoFisher Scientific Phospho-tau Clone AT8		Dickson		*Roemer SF, Grinberg LT, Crary JF, Seeley WW, McKee AC, Kovacs GG, Beach TG, Duyckaerts C, Ferrer IA, Gelpi E, Lee EB, Revesz T, White CL 3rd, Yoshida M, Pereira FL, Whitney K, Ghayal NB, Dickson DW. Rainwater*	

© 2026 Wu LY et al. *JAMA Neurology*.

											Charitable Foundation criteria for the neuropathologic diagnosis of progressive supranuclear palsy. Acta Neuropathol. 2022 Oct;144(4):603-614.	
	1985 - 1995		MSA									
	1995 - 2005											
	2005 - 2015			Quinn et al. (1994)								
	2015 - 2025			Quinn et al. (1994)	BD Transduction Clone 42				Trojanowski			
	1985 - 1995		other co-pa									
	1995 - 2005											

© 2026 Wu LY et al. *JAMA Neurology*.

	2005 - 2015								Braak, Thal			
	2015 - 2025						ThermoFisher Scientific Phospho-tau Clone AT8	Covance, 4G8, residues 17-24	Montine ABC, Nelson			
			Controls									
Banner Sun Health Research Institute	1985 - 1995		LBD							Anterior cingulate gyrus	10.1007/s10561-008-9067-2	Brain regions sampled and consent procedure apply across al time periods. Listed regions reflect only those relevant to cases included in this analysis and are not exhaustive of overall

© 2026 Wu LY et al. *JAMA Neurology*.

												brain bank practices
	1995 - 2005	Thomas G. Beach			polyclonal antibody raised against an α-synuclein peptide fragment phosphorylated at serine 129 (PMID: 18626651)	Gallyas	Campbell-Switzer	Thioflavine S, H&E		Amygdala		
	2005 - 2015	Thomas G. Beach			polyclonal antibody raised against an α-synuclein peptide fragment phosphorylated at serine 129 (PMID: 18626651)	Gallyas	Campbell-Switzer	Thioflavine S, H&E	USSLB (Beach et al., 2009), McKeith system	Entorhinal cortex	https://doi.org/10.1016/j.parkreldis.2014.02.012	

© 2026 Wu LY et al. *JAMA Neurology*.

	2015 - 2025	Thomas G. Beach			polyclonal antibody raised against an α-synuclein peptide fragment phosphorylated at serine 129 (PMID: 18626651)	Gallyas	Campbell-Switzer	Thioflavine S, H&E	USSLB (Beach et al., 2009), McKeith system	Middle frontal gyrus	10.1016/j.parkreldis.2011.02.017	
	1985 - 1995		PSP							Middle temporal gyrus	10.1212/NXG.0000000000000347	
	1995 - 2005	Thomas G. Beach			polyclonal antibody raised against an α-synuclein peptide fragment phosphorylated at serine 129 (PMID: 18626651)	Gallyas	Campbell-Switzer	Thioflavine S, H&E	NA	Inferior parietal lobule	10.1212/CPJ.0000000000001016	

© 2026 Wu LY et al. *JAMA Neurology*.

	2005 - 2015	Thomas G. Beach			polyclonal antibody raised against an α-synuclein peptide fragment phosphorylated at serine 129 (PMID: 18626651)	Gallyas	Campbell-Switzer	Thioflavine S, H&E	NA	Anterior medulla	10.1093/jnen/nlad025	
	2015 - 2025	Thomas G. Beach			polyclonal antibody raised against an α-synuclein peptide fragment phosphorylated at serine 129 (PMID: 18626651)	Gallyas	Campbell-Switzer	Thioflavine S, H&E	NA	Olfactory bulb		
	1985 - 1995		CBD									

© 2026 Wu LY et al. *JAMA Neurology*.

	1995 - 2005	Thomas G. Beach			polyclonal antibody raised against an α-synuclein peptide fragment phosphorylated at serine 129 (PMID: 18626651)	Gallyas; AT8 1: 1,000	Campbell-Switzer	Thioflavine S, H&E	NA			
	2005 - 2015	Thomas G. Beach			polyclonal antibody raised against an α-synuclein peptide fragment phosphorylated at serine 129 (PMID: 18626651)	Gallyas; AT8 1: 1,000	Campbell-Switzer	Thioflavine S, H&E	NA			

© 2026 Wu LY et al. *JAMA Neurology*.

	2015 - 2025	Thomas G. Beach			polyclonal antibody raised against an α-synuclein peptide fragment phosphorylated at serine 129 (PMID: 18626651)	Gallyas; AT8 1: 1,000	Campbell-Switzer	Thioflavine S, H&E	NA			
	1985 - 1995		MSA									
	1995 - 2005	Thomas G. Beach			polyclonal antibody raised against an α-synuclein peptide fragment phosphorylated at serine 129 (PMID: 18626651)	Gallyas	Campbell-Switzer	Thioflavine S, H&E	NA			

© 2026 Wu LY et al. *JAMA Neurology*.

	2005 - 2015	Thomas G. Beach			polyclonal antibody raised against an α-synuclein peptide fragment phosphorylated at serine 129 (PMID: 18626651)	Gallyas	Campbell-Switzer	Thioflavine S, H&E	NA			
	2015 - 2025	Thomas G. Beach			polyclonal antibody raised against an α-synuclein peptide fragment phosphorylated at serine 129 (PMID: 18626651)	Gallyas	Campbell-Switzer	Thioflavine S, H&E	NA			
	1985 - 1995		other co-pa			Gallyas	Campbell-Switzer	Thioflavine S, H&E	Braak NFT, CERAD			

© 2026 Wu LY et al. *JAMA Neurology*.

	1995 - 2005	Thomas G. Beach			polyclonal antibody raised against an α-synuclein peptide fragment phosphorylated at serine 129 (PMID: 18626651)	Gallyas	Campbell-Switzer	Thioflavine S, H&E	Braak NFT, CERAD			
	2005 - 2015	Thomas G. Beach			polyclonal antibody raised against an α-synuclein peptide fragment phosphorylated at serine 129 (PMID: 18626651)	Gallyas	Campbell-Switzer	Thioflavine S, H&E	Braak NFT, Thal, CERAD			

© 2026 Wu LY et al. *JAMA Neurology*.

	2015 - 2025	Thomas G. Beach			polyclonal antibody raised against an α-synuclein peptide fragment phosphorylated at serine 129 (PMID: 18626651)	Gallyas	Campbell-Switzer	Thioflavine S, H&E; TDP43 protein (2E2-E3 1:500	NIA-AA (Braak NFT, Thal, CERAD)			
			Controls	No major clinical neurological diagnosis & age-consistent neuropathology only								
Edinburgh Brain and Tissue Bank	1985 - 1995		LBD								10.1002/path.2247	
	1995 - 2005			McKeith et al. (1996)					McKeith system			
	2005 - 2015	CS		McKeith et al. (2005)	KM51 Leica				McKeith system			

© 2026 Wu LY et al. *JAMA Neurology*.

					NCL-L-ASYN							
	2015 - 2025	CS		McKeith et al. (2017)	KM51 Leica NCL-L-ASYN				McKeith system			
	1985 - 1995		PSP									
	1995 - 2005											
	2005 - 2015											
	2015 - 2025											
	1985 - 1995		CBD									
	1995 - 2005											
	2005 - 2015											
	2015 - 2025											
	1985 - 1995		MSA									
	1995 - 2005											
	2005 - 2015											
	2015 - 2025											

© 2026 Wu LY et al. *JAMA Neurology*.

	1985 - 1995		other co-pa									
	1995 - 2005											
	2005 - 2015	CS				AT8 Thermo MN1020	4G8 Biolegend 800712		Braak NFT, Thal,CERAD			
	2015 - 2025	CS		CS		AT8 Thermo MN1020	4G8 Biolegend 800712	CosmoBio CAC-TIP-PTD-MO1	Braak NFT, Thal,CERAD			
			Controls	with no know neurological disorders during life, and no significant neuropathology after detailed neuropathological examination								
Newcastle Brain Tissue Resource	1985 - 1995	RH Perry	LBD		KM51	Palmgren, Tau-2					https://doi.org/10.1186/s40478-023-01714-7	

© 2026 Wu LY et al. *JAMA Neurology*.

	1995 - 2005	RH Perry		McKeith et al. (1996)	KM51	AT8	4G8		McKeith system		https://doi.org/10.1007/s00401-020-02255-2	
	2005 - 2015	RH Perry/ J Attems		McKeith et al. (2005)	KM51	AT8	4G8		McKeith system, Braak LB		https://doi.org/10.1016/S0197-4580(02)00065-9	
	2015 - 2025	J Attems		McKeith et al. (2017)	KM51	AT8	4G8	pTDP-43 (Cosmo-Bio)	McKeith system, Braak LB, LPC		https://doi.org/10.1007/s00401-020-02255-2	
	1985 - 1995	RH Perry	PSP			Palmgren, Tau-2					https://doi.org/10.1093/brain/awz099	
	1995 - 2005	RH Perry			KM51	AT8	4G8					
	2005 - 2015	RH Perry/ J Attems			KM51	AT8	4G8	3R and 4R Tau staining	NA - pathology commensurate with PSP ie presence of cortical and basal ganglia tangles and neuropil		https://doi.org/10.1007/s00401-022-02479-4	

© 2026 Wu LY et al. *JAMA Neurology*.

									threads and tufted astrocytes			
	2015 - 2025	J Attems		Roemer et al, 2022, Acta Neuropathologica 144:603-14 (2022 onwards)	KM51	AT8	4G8	3R and 4R Tau staining	from 2022 Roemer et al, 2022, Acta Neuropathologica 144:603-14			
	1985 - 1995	RH Perry	CBD			Palmgren, Tau-2						
	1995 - 2005	RH Perry			KM51	AT8	4G8					
	2005 - 2015	RH Perry/ J Attems			KM51	AT8	4G8	3R and4R Tau staining				
	2015 - 2025	J Attems			KM51	AT8	4G8	3R and4R Tau staining				
	1985 - 1995	RH Perry	MSA			Palmgren, Tau-2]						
	1995 - 2005	RH Perry			KM51	AT8	4G8		NA-presence of alpha-synuclein GCI in oligodendrocytes and in multiple			

© 2026 Wu LY et al. *JAMA Neurology*.

									cortical white matter, basal ganglia, and subcortical nuclei, pons, inferior olives			
	2005 - 2015	RH Perry/ J Attems			KM51	AT8	4G8		NA- presence of alpha-synuclein GCI in multiple cortical white matter, basal ganglia, and subcortical nuclei, pons, inferior olives			
	2015 - 2025	J Attems			KM51	AT8	4G8		NA- presence of alpha-synuclein GCI in multiple			

© 2026 Wu LY et al. *JAMA Neurology*.

									cortical white matter, basal ganglia, and subcortical nuclei, pons, inferior olives			
	1985 - 1995	RH Perry	other co-pa		KM51	Palmgren, Tau2						
	1995 - 2005	RH Perry			KM51	AT8	4G8		Braak NFT, CERAD, BNE		https://doi.org/10.1007/bf00308809	
	2005 - 2015	RH Perry/ J Attems			KM51	AT8	4G8		Braak NFT, Thal,CERAD, BNE		https://doi.org/10.1007/s00401-009-0485-4	
	2015 - 2025	J Attems			KM51	AT8	4G8,dilution 1:15,000	pTDP-43 (Cosmo-Bio)	Braak NFT, Thal,CERAD, BNE		https://doi.org/10.1212/WNL.41.4.479	
			Controls						Braak NFT, Thal,CERAD, BNE		https://doi.org/10.1212/wnl.58.12.1791	

© 2026 Wu LY et al. *JAMA Neurology*.

Sydney Brain Bank	1985 - 1995		LBD									
	1995 - 2005											
	2005 - 2015											

© 2026 Wu LY et al. *JAMA Neurology*.

	2015 - 2025	SBB		Braak, McKeith, Attems	BD Transduction Laboratories, aa 15-123, cat#610787	phospho-PHF tau (AT8), Thermo Fisher Scientific cat#MN1020	2016-2024 Dako, clone 6F/3D, catalogue #M0872. 2024-to date Merck, clone W0-2, catalogue #MABN10	Anti-p62 lck ligand, BD Transduction laboratories, aa257-437, catalogue #610833. Anti-Phospho TDP-43, Cosmo Bio Co, clone 11-9, catalogue #TIP_PTD_M01	Braak LB, McKeith, LPC	superior and middle frontal gyri, anterior cingulate gyrus, inferior temporal gyrus, primary motor gyrus, hippocampus/entorhinal cortex, amygdala, caudate/putamen, midbrain, pons, medulla, cerebellum.	1. Braak H et al. Staging of brain pathology related to sporadic Parkinson's disease. Neurobiol Aging. 2003 Mar-Apr;24(2): 197-211. 2. McKeith IG et al. Consortium on DLB. Diagnosis and management of dementia with Lewy bodies: third report of the DLB Consortium. Neurology	all cases are retrospectively screened using new criteria, therefore only the latest criteria are listed

© 2026 Wu LY et al. *JAMA Neurology*.

											. 2005 Dec 27;65(12):1863-72. 3. Attems J et al. Neuropathological consensus criteria for the evaluation of Lewy pathology in post-mortem brains: a multi-centre study. Acta Neuropathol. 2021 Feb;141(2):159-172.	

© 2026 Wu LY et al. *JAMA Neurology*.

	1985 - 1995		PSP									
	1995 - 2005											
	2005 - 2015											
	2015 - 2025	SBB		Rainwater Charitable Foundation criteria	BD Transduction Laboratories, aa 15-123, cat#610787	phospho-PHF tau (AT8), Thermo Fisher Scientific cat#MN1020	2016-2024 Dako, clone 6F/3D, catalogue #M0872. 2024-to date Merck, clone W0-2, catalogue #MABN10	Anti-p62 lck ligand, BD Transduction laboratories, aa257-437, catalogue #610833. Anti-Phospho TDP-43, Cosmo Bio Co, clone 11-9, catalogue #TIP_PTD_M01	see ref	superior and middle frontal gyri, anterior cingulate gyrus, inferior temporal gyrus, primary motor gyrus, hippocampus/entorhinal cortex, amygdala, caudate/putamen, midbrain, pons, medulla, cerebellum,	Roemer SF et al. Rainwater Charitable Foundation criteria for the neuropathologic diagnosis of progressive supranuclear palsy. Acta Neuropathol. 2022 Oct;144(4):603-614	all cases are retrospectively screened using new criteria, therefore only the latest criteria are listed

© 2026 Wu LY et al. *JAMA Neurology*.

										subthalamus.		
	1985 - 1995		CBD									
	1995 - 2005											
	2005 - 2015											

© 2026 Wu LY et al. *JAMA Neurology*.

	2015 - 2025	SBB		Office of rare diseases	BD Transduction Laboratories, aa 15-123, cat#610787	phospho-PHF tau (AT8), Thermo Fisher Scientific cat#MN1020	2016-2024 Dako, clone 6F/3D, catalogue #M0872. 2024-to date Merck, clone W0-2, catalogue #MABN10	Anti-p62 lck ligand, BD Transduction laboratories, aa257-437, catalogue #610833. Anti-Phospho TDP-43, Cosmo Bio Co, clone 11-9, catalogue #TIP_PTD_M01	see ref	superior and middle frontal gyri, anterior cingulate gyrus, inferior temporal gyrus, primary motor gyrus, hippocampus/entorhinal cortex, amygdala, caudate/putamen, midbrain, pons, medulla, cerebellum. Other regions as needed eg, subthalamus,	Dickson DW et al. Office of Rare Diseases of the National Institutes of Health. Office of Rare Diseases neuropathologic criteria for corticobasal degeneration. J Neuropathol Exp Neurol. 2002 Nov;61(11):935-46	all cases are retrospectively screened using new criteria, therefore only the latest criteria are listed

© 2026 Wu LY et al. *JAMA Neurology*.

										globus pallidus.		
	1985 - 1995		MSA									
	1995 - 2005											
	2005 - 2015											

© 2026 Wu LY et al. *JAMA Neurology*.

	2015 - 2025	SBB		2nd consensus statement for MSA	BD Transduction Laboratories, aa 15-123, cat#610787	phospho-PHF tau (AT8), Thermo Fisher Scientific cat#MN1020	2016-2024 Dako, clone 6F/3D, catalogue #M0872. 2024-to date Merck, clone W0-2, catalogue #MABN10	Anti-p62 lck ligand, BD Transduction laboratories, aa257-437, catalogue #610833. Anti-Phospho TDP-43, Cosmo Bio Co, clone 11-9, catalogue #TIP_PTD_M01	2nd consensus statement for MSA	superior and middle frontal gyri, anterior cingulate gyrus, inferior temporal gyrus, primary motor gyrus, hippocampus/entorhinal cortex, amygdala, caudate/putamen, midbrain, pons, medulla, cerebellum, globus pallidus.	Gilman S et al. Second consensus statement on the diagnosis of multiple system atrophy. Neurology. 2008 Aug 26;71(9):670-6	all cases are retrospectively screened using new criteria, therefore only the latest criteria are listed
	1985 - 1995		other co-pa	Braak, Thal, CERAD, NIA-AAA								
	1995 - 2005											

© 2026 Wu LY et al. *JAMA Neurology*.

	2005 - 2015			AD neuropath								

© 2026 Wu LY et al. *JAMA Neurology*.

				ologic assessment of AD, LATE, PART and ARTAG consensus criteria, Esiri SVD								
	2015 - 2025	SBB			BD Transduction Laboratories, aa 15-123, cat#610787	phospho-PHF tau (AT8), Thermo Fisher Scientific cat#MN1020	2016-2024 Dako, clone 6F/3D, catalogue #M0872. 2024-to date Merck, clone W0-2, catalogue #MABN10	Anti-p62 lck ligand, BD Transduction laboratories, aa257-437, catalogue #610833. Anti-Phospho TDP-43, Cosmo Bio Co, clone 11-9, catalogue #TIP_PTD_M01	Braak, Thal, CERAD, NIA-AAA AD neuropathologic assessment of AD, LATE, PART and ARTAG consensus criteria, Esiri SVD	superior and middle frontal gyri, anterior cingulate gyrus, inferior temporal gyrus, primary motor gyrus, hippocampus/entorhinal cortex, amygdala, caudate/putamen, midbrain, pons, medulla, cerebellum. Other regions as required.	1. Hyman BT et al. National Institute on Aging-Alzheimer's Association guidelines for the neuropathologic assessment of Alzheimer's disease. Alzheimers Dement. 2012 Jan;8(1):1-13. 2. Shepherd CE et al. Condensing the Aβ protocol to reduce the effort and cost of NIA-AA guidelines for	all cases are retrospectively screened using new criteria, therefore only the latest criteria are listed

© 2026 Wu LY et al. *JAMA Neurology*.

											neuropathologic assessment of Alzheimer disease. J Neuropathol Exp Neurol. 2019 Oct 1;78(10):975-977. 3. Kovacs GG et al. Aging-related tau astrogliopathy (ARTAG): harmonized evaluation strategy. Acta Neuropathol. 2016 Jan;131(1):87-102. 4. Nelson PT et al. LATE-NC staging in routine	

© 2026 Wu LY et al. *JAMA Neurology*.

											neuropathologic diagnosis: an update. Acta Neuropathol. 2023 Feb;145(2):159-173. 5. Jellinger KA et al. PART, a distinct tauopathy, different from classical sporadic Alzheimer disease. Acta Neuropathol. 2015 May;129(5):757-62. 6. Esiri et al. Neuropathological assessment of the lesions of	

© 2026 Wu LY et al. *JAMA Neurology*.

											significance in vascular dementia. J Neurol Neurosurg Psychiatry. 1997 Dec;63(6):749-53.	

© 2026 Wu LY et al. *JAMA Neurology*.

	1985 - 1995											
	1995 - 2005											
	2005 - 2015											

© 2026 Wu LY et al. *JAMA Neurology*.

	2015 - 2025	SBB	Neurologically normal controls with only age-related pathology. Not meeting criteria for neurodegenerative disease.	Braak, Thal, CERAD, NIA-AAA AD neuropathologic assessment of AD, LATE, PART and ARTAG consensus criteria, Esiri SVD	BD Transduction Laboratories, aa 15-123, cat#610787	phospho-PHF tau (AT8), Thermo Fisher Scientific cat#MN1020	2016-2024 Dako, clone 6F/3D, catalogue #M0872. 2024-to date Merck, clone W0-2, catalogue #MABN10	Anti-p62 lck ligand, BD Transduction laboratories, aa257-437, catalogue #610833. Anti-Phospho TDP-43, Cosmo Bio Co, clone 11-9, catalogue #TIP_PTD_M01	Braak, Thal, CERAD, NIA-AAA AD neuropathologic assessment of AD, LATE, PART and ARTAG consensus criteria, Esiri SVD	superior and middle frontal gyri, anterior cingulate gyrus, inferior temporal gyrus, primary motor gyrus, hippocampus/entorhinal cortex, amygdala, caudate/putamen, midbrain, pons, medulla, cerebellum. Other regions as required.	1. Hyman BT et al. National Institute on Aging-Alzheimer's Association guidelines for the neuropathologic assessment of Alzheimer's disease. Alzheimers Dement. 2012 Jan;8(1):1-13. 2. Shepherd CE et al. Condensing the Aβ protocol to reduce the effort and cost of NIA-AA guidelines for	all cases are retrospectively screened using new criteria, therefore only the latest criteria are listed

© 2026 Wu LY et al. *JAMA Neurology*.

											neuropathologic assessment of Alzheimer disease. J Neuropathol Exp Neurol. 2019 Oct 1;78(10):975-977. 3. Kovacs GG et al. Aging-related tau astrogliopathy (ARTAG): harmonized evaluation strategy. Acta Neuropathol. 2016 Jan;131(1):87-102. 4. Nelson PT et al. LATE-NC staging in routine	

© 2026 Wu LY et al. *JAMA Neurology*.

											neuropathologic diagnosis: an update. Acta Neuropathol. 2023 Feb;145(2):159-173. 5. Jellinger KA et al. PART, a distinct tauopathy, different from classical sporadic Alzheimer disease. Acta Neuropathol. 2015 May;129(5):757-62. 6. Esiri et al. Neuropathological assessment of the lesions of	

© 2026 Wu LY et al. *JAMA Neurology*.

											significance in vascular dementia. J Neurol Neurosurg Psychiatry. 1997 Dec;63(6):749-53.	

© 2026 Wu LY et al. *JAMA Neurology*.

Manchester Brain Bank	1985 - 1995	Professor David Mann	LBD	n/a	n/a	n/a	n/a	n/a	n/a	frontal (mid frontal and superior frontal gyri)	10.3233/JAD-190580	
	1995 - 2005	Professor David Mann		n/a	n/a	n/a	n/a	n/a	n/a	cingulate		
	2005 - 2015	Professor David Mann		McKeith et al. (2017)	rabbit polyclonal antibody #1175, 1:1000	monoclonal antibody AT8, 1:750	Cambridge Bioscience, monoclonal antibody 4G8, 1:3000	TDP-43, Proteintech, 1:1000	Braak LB, McKeith system	temporal (including superior and middle temporal gyrus)		
	2015 - 2025	Professor Federico Roncaroli		Attems et al (2021)	BD mouse alpha-synuclein, 1:500	monoclonal antibody AT8, 1:750	Biolegend, monoclonal antibody 4G8, 1:3000 (changed to 1:2000 in 2024)	TDP-43, Proteintech, 1:1000 (changed to 1:3000 in 2022)	Lewy body consensus criteria	inferior parietal and occipital cortex		
	1985 - 1995	Professor David Mann	PSP	n/a	n/a	n/a	n/a	n/a	n/a	entorhinal cortex		

© 2026 Wu LY et al. *JAMA Neurology*.

	1995 - 2005	Professor David Mann		n/a	n/a	n/a	n/a	n/a	n/a	hippocampus		
	2005 - 2015	Professor David Mann		Hauw et al., (1994)	rabbit polyclonal antibody #1175, 1:1000	monoclonal antibody AT8, 1:750	Cambridge Bioscience, monoclonal antibody 4G8, 1:3000	TDP-43, Proteintech, 1:1000	n/a	amygdala		
	2015 - 2025	Professor Federico Roncaroli		Roemer et al (2022)	BD mouse alpha-synuclein, 1:500	monoclonal antibody AT8, 1:750	Biolegend, monoclonal antibody 4G8, 1:3000 (changed to 1:2000 in 2024)	TDP-43, Proteintech, 1:1000 (changed to 1:3000 in 2022)	Rainwater criteria	corpus striatum (caudate nucleus, putamen, and globus pallidus)		
	1985 - 1995	Professor David Mann	CBD	n/a	n/a	n/a	n/a	n/a	n/a	thalamus		
	1995 - 2005	Professor David Mann		n/a	n/a	n/a	n/a	n/a	n/a	midbrain (to include substantia nigra)		

© 2026 Wu LY et al. *JAMA Neurology*.

	2005 - 2015	Professor David Mann		Dickson et al. (2002)	rabbit polyclonal antibody #1175, 1:1000	monoclonal antibody AT8, 1:750	Cambridge Bioscience, monoclonal antibody 4G8, 1:3000	TDP-43, Proteintech, 1:1000	n/a	brainstem (to include locus coeruleus and dorsal vagus)		
	2015 - 2025	Professor Federico Roncaroli		Dickson et al. (2002)	BD mouse alpha-synuclein, 1:500	monoclonal antibody AT8, 1:750	Biolegend, monoclonal antibody 4G8, 1:3000 (changed to 1:2000 in 2024)	TDP-43, Proteintech, 1:1000 (changed to 1:3000 in 2022)	n/a	cerebellum with dentate nucleus		
	1985 - 1995	Professor David Mann	MSA	n/a	n/a	n/a	n/a	n/a	n/a			
	1995 - 2005	Professor David Mann		n/a	n/a	n/a	n/a	n/a	n/a			
	2005 - 2015	Professor David Mann		Trojanowski et al. (2007)	rabbit polyclonal antibody #1175, 1:1000	monoclonal antibody AT8, 1:750	Cambridge Bioscience, monoclonal antibody	TDP-43, Proteintech, 1:1000	n/a			

© 2026 Wu LY et al. *JAMA Neurology*.

							4G8, 1:3000					
	2015 - 2025	Professor Federico Roncaroli		Trojanowski et al. (2007)	BD mouse alpha-synuclein, 1:500	monoclonal antibody AT8, 1:750	Biolegend, monoclonal antibody 4G8, 1:3000 (changed to 1:2000 in 2024)	TDP-43, Proteintech, 1:1000 (changed to 1:3000 in 2022)	n/a			
	1985 - 1995	Professor David Mann	AD and other co-pathology	n/a	n/a	n/a	n/a	n/a	n/a			
	1995 - 2005	Professor David Mann		n/a	n/a	n/a	n/a	n/a	n/a			
	2005 - 2015	Professor David Mann		NIA-AA	rabbit polyclonal antibody #1175, 1:1000	monoclonal antibody AT8, 1:750	Cambridge Bioscience, monoclonal antibody 4G8, 1:3000	TDP-43, Proteintech, 1:1000	Braak NFT, Thal,CERAD, BNE			

© 2026 Wu LY et al. *JAMA Neurology*.

	2015 - 2025	Professor Federico Roncaroli		NIA-AA	BD mouse alpha-synuclein, 1:500	monoclonal antibody AT8, 1:750	Biolegend, monoclonal antibody 4G8, 1:3000 (changed to 1:2000 in 2024)	TDP-43, Proteintech, 1:1000 (changed to 1:3000 in 2022)	Braak NFT, Thal,CERAD, BNE			
			Controls									
London Neurodegenerative Diseases Brain Bank	1985 - 1995		LBD							Middle frontal gyrus at the level of the genu of the corpus callosum (BA 8/9)	https://doi.org/10.1007/s00702-015-1410-8	
	1995 - 2005			McKeith et al. (1996)					McKeith system, Braak LB	Superior and middle temporal gyri at the level of the lateral geniculate body (BA 21).	10.1007/s00702-015-1376-6	

© 2026 Wu LY et al. *JAMA Neurology*.

	2005 - 2015			McKeith et al. (2005)					McKeith system, Braak LB, BNE	Anterior hippocampus and parahippocampal gyrus at the same level as (2) (BA29).		
	2015 - 2025			McKeith et al. (2017)	BD Transduction Labs (610786)	AT8 (Invitrogen, MN1020)	bA4	H&E,TDP 43, P62	McKeith system, Braak LB, BNE	The superior sulcus margin (intraparietal sulcus) of the parietal lobe in plane 1 cm posterior to the posterior pole of the splenium (BA40).		
	1985 - 1995		PSP							Hemi-midbrain at the level of the third nerve		
	1995 - 2005			Litvan I et al. Validity and						Superior frontal gyrus at		

© 2026 Wu LY et al. *JAMA Neurology*.

				reliability of the preliminary NNIPS neuropath ologic criteria for progressiv e supranucle ar palsy and related disorder. J Neuropath ol Exp Neuro 1996;55:9 7-105						the level of the genu of the corpus callosum and including the cingulated gyrus (BA 24).		
	2005 - 2015			Litvan I et al. Validity and reliability of the preliminary NNIPS neuropath ologic criteria for progressiv e supranucle ar palsy and						Amygdala and anterior entorhinal cortex		

© 2026 Wu LY et al. *JAMA Neurology*.

				related disorder. J Neuropathol Exp Neuro 1996;55:97-105								
	2015 - 2025			Litvan I et al. Validity and reliability of the preliminary NNIPS neuropathologic criteria for progressive supranuclear palsy and related disorder. J Neuropathol Exp Neuro 1996;55:97-105	BD Transduction Labs (610786)	AT8 (Invitrogen, MN1020)	bA4	H&E,TDP43, P62		Pons, rostral part through the mid-point of the locus coeruleus		
	1985 - 1995		CBD							Medulla through the maximum		

© 2026 Wu LY et al. *JAMA Neurology*.

										diameter of the inferior olive.		
	1995 - 2005											
	2005 - 2015			Dickson et al. Neuropathological criteria of corticobasal degeneration. J Neuropathology Exp Neuro 2002. 61:935-946								
	2015 - 2025			Dickson et al. Neuropathological criteria of corticobasal degeneration. J Neuropathology Exp	BD Transduction Labs (610786)	AT8 (Invitrogen, MN1020)	bA4	H&E,TDP43, P62				

© 2026 Wu LY et al. *JAMA Neurology*.

				Neuro 2002. 61:935-946								
	1985 - 1995		MSA									
	1995 - 2005			Chin SS M and Goldman JE. Journal of Neuropathology and Experimental Neurology 1996;55: 499-508. Lantos PL. Journal of Neuropathology and Experimental Neurology 1998;57: 1099-1111								
	2005 - 2015			Chin SS M and								

© 2026 Wu LY et al. *JAMA Neurology*.

				Goldman JE. Journal of Neuropathology and Experimental Neurology 1996;55: 499-508. Lantos PL. Journal of Neuropathology and Experimental Neurology 1998;57: 1099-1111								
	2015 - 2025			Chin SS M and Goldman JE. Journal of Neuropathology and Experimental Neurology 1996;55: 499-508. Lantos PL.	BD Transduction Labs (610786)	AT8 (Invitrogen, MN1020)	bA4	H&E,TDP43, P62				

© 2026 Wu LY et al. *JAMA Neurology*.

				Journal of Neuropathology and Experimental Neurology 1998;57: 1099-1111								
	1985 - 1995		other co-pa					H&E, Bielschowsky				
	1995 - 2005			Mirra SS et al 1991. Neurology 41; 479-486 Braak H and Braak E. 1991. Acta Neuropathologica 82; 239-259		Gallyas		H&E, Bielschowsky, ubiquitin				

© 2026 Wu LY et al. *JAMA Neurology*.

	2005 - 2015			Alafuzoff I et al. (2006) Inter-laboratory comparison of assessments of Alzheimer disease-related lesions: a study of the BrainNet Europe Consortium. J Neuropathol Exp Neurol, 65, 740-57. Alafuzoff I et al. (2008a) Staging of Neurofibrillary Pathology in Alzheimer's Disease: A Study of		Gallyas			Braak NFT, Thal,CERAD, BNE			

© 2026 Wu LY et al. *JAMA Neurology*.

				the BrainNet Europe Consortium. Brain Pathol.								
	2015 - 2025			Modified Braak/BNE staging (Alafuzoff et al. 2008), NIA-AA		AT8 (Invitrogen, MN1020)	anti-β-amyloid (clone A4, 1:6000)	TDP43, P62	Braak NFT, Thal,CERAD, BNE			
			Controls	No neurodegenerative diseases								
South West Dementia Brain Bank	1985 - 1995	Tim Moss & Seth Love	LBD	Textbook/Braak LB stage	None/anti-asyn KM51	None(silver impreg)/AT8	None (silver impreg & thioflavin)/4G8		Braak LB stage			
	1995 - 2005	Seth Love		McKeith et al 1996/Braak LB stage	Anti-ubiquitin/anti-asyn KM51	AT8	DAKO M0872		Braak LB stage	see McKeith et al 1996		
	2005 - 2015	Seth Love		Braak LB stage	Anti-ubiquitin/anti-asyn KM51	AT8	4G8		Braak LB stage	As needed for Braak LB staging		

© 2026 Wu LY et al. *JAMA Neurology*.

	2015 - 2025	Seth Love and Johannes Attems		Braak LB stage + unified LB staging scheme	KM51	AT8	4G8		Braak LB stage + unified LB staging scheme	As needed for Braak LB staging		
	1985 - 1995	Tim Moss & Seth Love	PSP	NINDS criteria	None/anti-asyn KM51	None(silver impreg)/AT8	None (silver impreg & thioflavin)/ 4G8					
	1995 - 2005	Seth Love		NINDS criteria	Anti-ubiquitin/anti-asyn KM51	AT8	DAKO M0872					
	2005 - 2015	Seth Love		NINDS criteria	Anti-ubiquitin/anti-asyn KM51	AT8	4G8					
	2015 - 2025	Seth Love and Johannes Attems		NINDS criteria	KM51	AT8	4G8					
	1985 - 1995	Tim Moss & Seth Love	CBD	Textbook	None/anti-asyn KM51	None(silver impreg)/AT8	None (silver impreg & thioflavin)/ 4G8					
	1995 - 2005	Seth Love		Textbook	Anti-ubiquitin/anti-asyn KM51	AT8	DAKO M0872					

© 2026 Wu LY et al. *JAMA Neurology*.

	2005 - 2015	Seth Love		Textbook	Anti-ubiquitin/anti-asyn KM51	AT8	4G8					
	2015 - 2025	Seth Love and Johannes Attems		Textbook	KM51	AT8	4G8					
	1985 - 1995	Tim Moss & Seth Love	MSA	Textbook	None/anti-asyn KM51	None(silver impreg)/AT8	None (silver impreg & thioflavin)/4G8					
	1995 - 2005	Seth Love		Textbook	Anti-ubiquitin/anti-asyn KM51	AT8	DAKO M0872					
	2005 - 2015	Seth Love		Textbook	Anti-ubiquitin/anti-asyn KM51	AT8	4G8					
	2015 - 2025	Seth Love and Johannes Attems		Textbook	KM51	AT8	4G8					
	1985 - 1995	Tim Moss & Seth Love	AD and other co-pathology	Textbook/CERAD/NIA-AA	None	None(silver impreg)	None/4G8		Braak NFT, Thal,CERAD, BNE			
	1995 - 2005	Seth Love		CERAD/NIA-AA	Anti-ubiquitin/a	AT8	DAKO M0872		Braak NFT,			

© 2026 Wu LY et al. *JAMA Neurology*.

					nti-asyn KM51				Thal,CERAD, BNE			
	2005 - 2015	Seth Love		CERAD/NIA-AA	Anti-ubiquitin/anti-asyn KM51	AT8	4G8		Braak NFT, Thal,CERAD, BNE			
	2015 - 2025	Seth Love and Johannes Attems		CERAD/NIA-AA	KM51	AT8	4G8		Braak NFT, Thal,CERAD, BNE			
			Controls	Exclusion of other diseases								
Victoria Brain Bank	1985 - 1995	McLean	LBD							Midbrain	ISBN 9781003389699	
	1995 - 2005	McLean								Pons	DOI:10.1002/9781444341256	
	2005 - 2015	McLean			Antibody obtained from FLOREY					Medulla		
	2015 - 2025	McLean			Antibody obtained from FLOREY				Braak LB, McKeith system	Frontal cortex		
	1985 - 1995	McLean	PSP							Corpus callosum		

© 2026 Wu LY et al. *JAMA Neurology*.

	1995 - 2005	McLean								Insular cortex		
	2005 - 2015	McLean		NINDS-Society for PSP neuropathologic criteria (Hauw 1994)		Dako (order code: A002401-2)				Basal ganglia		
	2015 - 2025	McLean		NINDS-Society for PSP neuropathologic criteria (Hauw 1994)		Dako (order code: A002401-2)				Hippocampus		
	1985 - 1995	McLean	CBD							Temporal lobe		
	1995 - 2005	McLean								Occipital cortex		
	2005 - 2015	McLean		Armstrong et al. consensus neuropathologic criteria (2013)		Dako (order code: A002401-2)				Thalamus		
	2015 - 2025	McLean		Armstrong et al. consensus		Dako (order code:						

© 2026 Wu LY et al. *JAMA Neurology*.

				neuropathologic criteria (2013)		A002401-2)						
	1985 - 1995	McLean	MSA									
	1995 - 2005	McLean										
	2005 - 2015	McLean		MSA Neuropathology Working Group consensus criteria (2008)	Antibody obtained from FLOREY							
	2015 - 2025	McLean		MSA Neuropathology Working Group consensus criteria (2008)	Antibody obtained from FLOREY							
	1985 - 1995	McLean	other co-pa									
	1995 - 2005	McLean										
	2005 - 2015	McLean				Dako (order code:	Dako (order code: M0872)	UNITED BIORESEARCH, (order				

© 2026 Wu LY et al. *JAMA Neurology*.

						A002401-2)		code: CAC-TIP-PTD-M01)				
	2015 - 2025	McLean				Dako (order code: A002401-2)	Dako (order code: M0872)	UNITED BIORESEARCH, (order code: CAC-TIP-PTD-M01)	Braak NFT, Thal,CERAD			
			Controls	no known neurological deficits and died from other causes - AMI/etc								

Summary of neuropathological practices across participating brain banks, compiled in collaboration with each contributing centre. The table details antibodies, diagnostic criteria applied and regions evaluated during the time period in which cases included in this study were collected. This information is provided to enhance methodological transparency and to document variability in neuropathological assessments across centres and over time. Where available, information was provided by the contributing brain bank; however, documentation for earlier decades was occasionally incomplete, and some entries may therefore be missing.

eTable 12. Key Resource Table

RESOURCE TYPE	RESOURCE NAME	SOURCE	IDENTIFIER	NEW/RE USE	ADDITIONAL INFORMATION
Dataset	Defining and Diagnosing neurodegenerative Movement Disorders through integrated analysis of Genetics and	AMP-PD	DOI: 10.1002/mds.28494	NEW	All data generated in MD-GAP are now part of GP2. Full data can be accessed here: https://amp-pd.org/register-for-amp-

© 2026 Wu LY et al. *JAMA Neurology*.

	neuroPathology (MD-GAP)/Global Parkinson's Genetics Program (GP2)				pd
Software/code	R code: Analysis Script	Github	https://github.com/huw-morris-lab/MD-GAP-GP2-CPC.git	NEW	
Software/code	GenoTools	https://github.com/dvitale199/GenoTools	https://doi.org/10.1093/g3journal/jkae268	REUSE	
Software/code	R Project for Statistical Computing, v4.3.1	https://www.r-project.org/	RRID:SCR_001905	REUSE	
Software/code	R package: dplyr	https://cran.r-project.org/web/packages/dplyr/index.html	RRID:SCR_016708	REUSE	
Software/code	R package: data.table	https://github.com/Rdatatable/data.table	RRID:SCR_026117	REUSE	
Software/code	R package: readxl	https://cran.r-project.org/web/packages/readxl/index.html	RRID:SCR_018083	REUSE	
Software/code	R package: stringr	https://stringr.tidyverse.org/	RRID:SCR_022813	REUSE	
Software/code	R package: ggplot2	https://cran.r-project.org/web/packages/ggplot2/index.html	RRID:SCR_014601	REUSE	
Software/code	R package: scales	https://CRAN.R-project.org/package=scales	RRID:SCR_019295	REUSE	
Software/code	R package: RColorBrewer	https://cran.r-project.org/web/packages/RColorBrewer/index.html	RRID:SCR_016697	REUSE	

© 2026 Wu LY et al. *JAMA Neurology*.

Software/code	R package: tidyverse	https://CRAN.R-project.org/package=tidyverse	RRID:SCR_019186	REUSE	
Software/code	R package: pheatmap	https://www.rdocumentation.org/packages/pheatmap/versions/0.2/topics/pheatmap	RRID:SCR_016418	REUSE	
Software/code	R package: gridExtra	https://CRAN.R-project.org/package=gridExtra	RRID:SCR_025249	REUSE	
Software/code	R package: MASS	https://CRAN.R-project.org/package=MASS	RRID:SCR_019125	REUSE	
Software/code	R package: survival	https://CRAN.R-project.org/view=Survival	RRID:SCR_026244	REUSE	
Software/code	R package: survminer	https://rdocumentation.org/packages/survminer/versions/0.4.9	RRID:SCR_021094	REUSE	
Software/code	R package: broom	https://cran.r-project.org/web/packages/broom/index.html	RRID:SCR_026874	REUSE	
Software/code	Plink2	https://www.cog-genomics.org/plink/2.0/	RRID:SCR_001757	REUSE	

List of resources generated and used for this project.

© 2026 Wu LY et al. *JAMA Neurology*.

eTable 13. Performance of NeuroBooster Array (NBA) probes compared with short-read Genome Sequencing (GS) for neurodegenerative movement disorder associated variants.

Gene	HGVS	Illumina probe name	TP	TN	FP	FN	Sensitivity	Specificity
GBA1	p.Asp448His	**1:155205518-G-C**	0	2211	0	3	0	1
GBA1	p.Asp448His	ilmnseq_rs1064651	0	2211	0	3	0	1
GBA1	p.Asp448His	ilmnseq_rs1064651_ilmndup2_ilmnbot	0	2211	0	3	0	1
GBA1	p.Asp448His	rs1064651	0	2211	0	3	0	1
GBA1	p.Glu365Lys	**chr1:155236376:C:T**	93	2119	1	1	0.989361702	0.999528302
GBA1	p.Glu365Lys	kgp308887	93	2119	1	1	0.989361702	0.999528302
GBA1	p.Ile299Thr	seq-rs794727908	0	2213	1	0	-	0.999548329
GBA1	p.Ile299Thr	**Seq_rs794727908**	0	2214	0	0	-	1

© 2026 Wu LY et al. *JAMA Neurology*.

GBA1	p.Asn409Ser	exm106217	12	2199	1	2	0.8571 42857	0.9995 45455
GBA1	p.Asn409Ser	ilmnseq_rs76763715.1_F2BT	9	2199	1	5	0.6428 57143	0.9995 45455
GBA1	p.Asn409Ser	ilmnseq_rs76763715.2_F2BT	0	2200	0	14	0	1
GBA1	p.Asn409Ser	Seq_rs76763715.2_ilmnfwd_il mnF2BT	0	2200	0	14	0	1
GBA1	p.Asn409Ser	**chr1:155235843:T:C**	14	2198	2	0	1	0.9990 90909
GBA1	p.Arg170Cys	**chr1:155208388-155208388_G_T**	0	2212	0	2	0	1
GBA1	p.Arg170Cys	rs398123530	0	2212	0	2	0	1
GBA1	p.Arg502Cys	**1:155204987-C-T**	4	2210	0	0	1	1
GBA1	p.Arg502Cys	Seq_rs80356771.1_ilmnrev_il mnF2BT	4	2210	0	0	1	1

© 2026 Wu LY et al. *JAMA Neurology*.

GBA1	p.Arg502Cys	Seq_rs80356771.2_ilmnrev_ilmnF2BT	0	2210	0	4	0	1
GBA1	p.Arg502Cys	rs80356771	4	2210	0	0	1	1
GBA1	c.115+1G>A	**rs104886460**	0	2213	0	1	0	1
GBA1	p.Thr362Ile	**1:155206175-C-T**	1	2213	0	0	1	1
GBA1	p.Thr362Ile	rs76539814	0	2213	0	1	0	1
GBA1	p.Thr362Ile	Seq_rs76539814	1	2213	0	0	1	1
GBA1	p.Thr408Met	exm106220	42	2167	2	3	0.933333333	0.999077916
GBA1	p.Thr408Met	rs75548401	39	2167	2	6	0.866666667	0.999077916
GBA1	p.Thr408Met	**seq_rs75548401**	43	2167	2	2	0.955555556	0.999077916
LRRK2	p.Gly2019Ser	**exm994671**	12	2202	0	0	1	1

© 2026 Wu LY et al. *JAMA Neurology*.

LRRK2	p.Gly2019Ser	rs34637584	12	2202	0	0	1	1
LRRK2	p.Gly2019Ser	seq_rs34637584	12	2202	0	0	1	1
LRRK2	p.Gly2385Asp	**exm994721**	0	2214	0	0	-	1
LRRK2	p.Gly2385Asp	rs34778348	0	2214	0	0	-	1
LRRK2	p.Gly2385Asp	seq_rs34778348	0	2214	0	0	-	1
LRRK2	p.Arg1441His	**12:40704237-G-A**	0	2214	0	0	-	1
LRRK2	p.Arg1441His	Seq_rs34995376	0	2214	0	0	-	1
LRRK2	p.Tyr1699Cys	**12:40714916-A-G**	1	2213	0	0	1	1
LRRK2	p.Tyr1699Cys	Seq_rs35801418	1	2213	0	0	1	1

© 2026 Wu LY et al. *JAMA Neurology*.

PRKN	p.Cys253Tyr	**PARK2:NM_004562.2:c.758 G>T:p.(Cys253Phe)**	0	2214	0	0	-	1
PRKN	p.Gly430Asp	**Seq_rs191486604**	2	2212	0	0	1	1
PRKN	p.Gly430Asp	exm593474	2	2212	0	0	1	1
PRKN	p.Asn52Metf sTer24	**Variant13588**	1	2213	0	0	1	1
PRKN	p.Asn52Metf sTer24	Seq_rs754809877	1	2213	0	0	1	1
PRKN	p.Arg275Trp	exm593505	19	2194	0	1	0.95	1
PRKN	p.Arg275Trp	**rs34424986**	20	2194	0	0	1	1
PRKN	p.Arg275Trp	Seq_rs34424986.1_ilmnrev_il mnF2BT	6	2194	0	14	0.3	1
PRKN	p.Arg275Trp	Seq_rs34424986.2_ilmnrev_il mnF2BT	0	2194	0	20	0	1
PRKN	p.Trp74Cysfs	**indel.94051**	1	2213	0	0	1	1

© 2026 Wu LY et al. *JAMA Neurology*.

	Ter8							
PSAP	p.Met1Leu	**10:73610978-A-T**	1	2213	0	0	1	1
PSAP	p.Met1Leu	Seq_rs121918106.1_ilmnrev_ilmnF2BT	1	2213	0	0	1	1
PSAP	p.Met1Leu	Seq_rs121918106.2_ilmnrev_ilmnF2BT	0	2212	1	1	0	0.999548125
SNCA	p.Gly51Asp	**seq-rs431905511-B1**	1	2213	0	0	1	1
VPS35	p.Asp620Asn	**16:46696364-G-A**	0	2214	0	0	-	1

The proportion of true positive (TP), true negative (TN), false positive (FP) and false negative (FN) genotype calls, and probe sensitivity and specificity, were calculated for the 51 probes targeting mutations detected in the 2214 individuals who underwent both NeuroBooser Array (NBA) genotyping and short-read Genome Sequencing. The most accurate probes were selected for analysis, shown in bold.

© 2026 Wu LY et al. *JAMA Neurology*.

eTable 14. Variants with NeuroBooster Array (NBA) probes not detected in short-read Genome Sequencing (GS).

Gene	HGVS	Illumina probe name
GBA1	p.Leu483Pro	1:155205043-T-C
GBA1	p.Ser235Pro	Variant49129
LRRK2	p.Ile1122Val	12:40692927-A-G
LRRK2	p.Ile1122Val	Seq_rs34805604
LRRK2	p.Ile2020Thr	12:40734206-T-C
LRRK2	p.Ile2020Thr	Seq_rs35870237
LRRK2	p.Asn1437Asp	12:40703027-A-C
LRRK2	p.Asn1437Asp	Seq_rs74163686
LRRK2	p.Arg1441Cys	Seq_rs33939927.1_ilmnfwd_ilmnF2BT
LRRK2	p.Arg1441Cys	Seq_rs33939927.2_ilmnfwd_ilmnF2BT
LRRK2	p.Arg1441Cys	Seq_rs33939927.3_ilmnfwd_ilmnF2BT
LRRK2	p.Arg1441Cys	var_12_40704236
PINK1	p.Leu347Pro	1:20972133-T-C
PINK1	p.Leu347Pro	Seq_rs28940285
PINK1	p.Leu347Pro	rs28940285
PRKN	p.Gln34ArgfsTer5	Variant17166
PRKN	p.Cys431Phe	6:161771237-G-T
PRKN	p.Glu395Ter	PARK2:NM_004562.2:c.1183G>T:p.(Glu395*)
PRKN	p.Arg42Pro	1:35250488-G-C

© 2026 Wu LY et al. *JAMA Neurology*.

SNCA	p.Ala30Pro	rs104893878
SNCA	p.Ala53Thr	seq-rs104893877-B1
SNCA	p.Glu46Lys	4:90749321-G-A

A table listing additional variants of interest and corresponding Illumina NeuroBooster Array (NBA) probe names. These variants were not identified in short-read Genome Sequencing, hence probe performance could not be assessed.

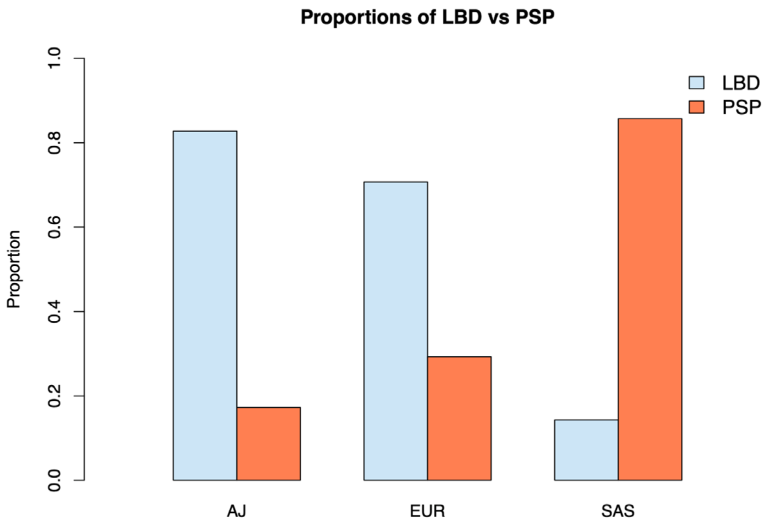

eFigure. Proportion of LBD and PSP diagnoses across ancestry groups.

Bar plot showing the relative proportion of Lewy body disease (LBD) and progressive supranuclear palsy (PSP) in individuals of Ashkenazi Jewish (AJ), European (EUR) and South Asian (SAS) ancestry. Mutation carriers were excluded from this analysis to avoid confounding effects from *GBA1* and *LRRK2*, which are known to be enriched in AJ. PSP was more frequent in SAS compared with EUR and AJ, while LBD was more frequent in AJ ($p < 0.0001$).

eReferences

1. Malek N, Weil RS, Bresner C, et al. Features of GBA-associated Parkinson’s disease at presentation in the UK Tracking Parkinson's study. *J*

© 2026 Wu LY et al. *JAMA Neurology*.

Neurol Neurosurg Psychiatry. 2018;89(7):702-709.

2. Menon PJ, Sambin S, Criniere-Boizet B, et al. Genotype-phenotype correlation in PRKN-associated Parkinson's disease. *NPJ Parkinsons Dis*. 2024;10(1):72.

3. Morales-Briceno H, Ong TL, Duma SR, et al. Recurrent biallelic p.L347P PINK1 variant in Polynesians with parkinsonism and isolated dopa-responsive dystonia. *Mov Disord Clin Pract*. 2022;9(5):696-697.

4. Chen YP, Gu XJ, Ou RW, et al. Genetic analysis of prosaposin, the lysosomal storage disorder gene in Parkinson's disease. *Mol Neurobiol*. 2021;58(4):1583-1592.

5. Beach TG, Adler CH, Sue LI, et al. Arizona Study of Aging and Neurodegenerative Disorders and brain and Body Donation Program. *Neuropathology*. 2015;35(4):354-389.

6. Alafuzoff I, Ince PG, Arzberger T, et al. Staging/typing of Lewy body related α-synuclein pathology: a study of the BrainNet Europe Consortium. *Acta Neuropathol*. 2009;117(6):635-652.

© 2026 Wu LY et al. *JAMA Neurology*.
