# Supplementary material for: Pathology and Genetics in a Global Cohort of Parkinsonian Disorders
Source: JAMA Neurol. 2026 Jun 8:e261634. Online ahead of print. doi: 10.1001/jamaneurol.2026.1634 (PMC13247843; doi:10.1001/jamaneurol.2026.1634)
Supplement: Supplement 2. — Nonauthor Collaborators. Global Parkinson’s Genetic Program (GP2). [file jamaneurol-e261634-s002.pdf]

*First name, last name, and suffix (if applicable) are required and will appear in PubMed.

*Group Name(s): Global Parkinson's Genetic Program (GP2)							
***First Name and Middle Initial(s)**	***Last Name**	***Suffix (eg, Jr, III)**	Academic Degrees	Institution	Location (city, state/province, country)	Role or Contribution, eg, chair, principal investigator	Group (if more than 1 Group listed in the byline) and/or Subgroup (eg, Steering Committee)
Yasser	Mecheri			Centre Hospitalo-Universitaire Dr Benbadis Constantine	Constantine, Algeria	Data Contributor	GP2
Bouchetara Mohamed	Sofiane			Hospital university of Oran-Algeria	Oran, Algeria	Data Contributor	GP2
Benhassine	Traki			Faculty of Biological Sciences, USTHB Bab Ezzouar, Algiers	Algiers, Algeria	Data Contributor	GP2
Emilia M	Gatto			Sanatorio de la Trinidad Mitre-INEBA	Buenos Aires, Argentina	Data Contributor	GP2
Marcelo	Kauffman			Hospital JM Ramos Mejia	Buenos Aires, Argentina	Data Contributor	GP2
Federico	Capparelli			Centro de Educación Médica e Investigaciones Clínicas Norberto Quirno	Buenos Aires, Argentina	Data Contributor	GP2
Maria Valentina	Muller			Hospital General San Martin	La Plata, Argentina	Data Contributor	GP2
Marcela Susana	Tela			Hospital Fernandez	Buenos Aires, Argentina	Data Contributor	GP2
Adamec, Dario	Sergio			HOSPITAL NACIONAL PROFESOR ALEJANDRO POSADAS	Buenos Aires, Argentina	Data Contributor	GP2
Cesar Luis	Avila			CONICET-UNT	San Miguel de Tucumán, Argentina	Data Contributor	GP2
Valentin	Selvaggi			Hospital Ramos Mejia	Buenos Aires, Argentina	Data Contributor	GP2
Nahuel	Fonseca			Consejo Nacional de Investigaciones Científicas y Técnicas	Buenos Aires, Argentina	Data Contributor	GP2
Elena	AVALE			CONICET	Buenos Aires, Argentina	Data Contributor	GP2

Supplemental Online Content: Nonauthor Collaborators
*First name, last name, and suffix (if applicable) are required and will appear in PubMed.

*First Name and Middle Initial(s)	*Last Name	*Suffix (eg, Jr, III)	Academic Degrees	Institution	Location (city, state/province, country)	Role or Contribution, eg, chair, principal investigator	Group (if more than 1 Group listed in the byline) and/or Subgroup (eg, Steering Committee)
Marcelo	Merello			Fleni	Buenos Aires, Argentina	Data Contributor	GP2
Griselda Judith	Alvarado			SIPROSA	Tucumán, Argentina	Data Contributor	GP2
Juan Pablo Diaz	Rearte			SIPROSA	Tucumán, Argentina	Data Contributor	GP2
Luciana Rojas	Vazquez			SIPROSA	Tucumán, Argentina	Data Contributor	GP2
Lucía	Wang			PARKINSON ARGENTINA/ALAPA	Buenos Aires, Argentina	Data Contributor	GP2
Carlos Matias López	Razquin			H.I.G.A. "Gral. San Martín" - ALAPA	La Plata, Argentina	Data Contributor	GP2
Andrea	Bril			Hospital Fernández	Buenos Aires, Argentina	Data Contributor	GP2
Florencia Nicole	Wainberg			Fleni	Buenos Aires, Argentina	Data Contributor	GP2
Maria Fernanda	López			Hospital Nacional Alejandro Posadas. Argentina	El Palomar, Argentina	Data Contributor	GP2
Samson	Khachatryan			Somnus Neurology Clinic	Yerevan, Armenia	Data Contributor	GP2
Zaruhi	Tavadyan			Somnus Neurology Clinic	Yerevan, Armenia	Data Contributor	GP2
Mariam	Isayan			Somnus Neurology Clinic	Yerevan, Armenia	Data Contributor	GP2
Claire E	Shepherd			Neuroscience Research Australia	Sydney, Australia	Data Contributor	GP2
Kishore	Kumar			Garvan Institute of Medical Research and Concord Repatriation General Hospital	Darlinghurst, Australia	Data Contributor	GP2
Melina	Ellis			Concord Hospital	Concord, Australia	Data Contributor	GP2
Miguel E.	Rentería			QIMR Berghofer Medical Research Institute	Herston, Australia	Data Contributor	GP2
Sulev	Koks			Murdoch University	Perth, Australia	Data Contributor	GP2
Simon	Rowe			Neuroscience Research Australia	Sydney, Australia	Data Contributor	GP2
Dennis	Yeow			Neuroscience Research Australia	Sydney, Australia	Data Contributor	GP2

Supplemental Online Content: Nonauthor Collaborators
*First name, last name, and suffix (if applicable) are required and will appear in PubMed.

***First Name and Middle Initial(s)**	***Last Name**	***Suffix (eg, Jr, III)**	Academic Degrees	Institution	Location (city, state/province, country)	Role or Contribution, eg, chair, principal investigator	Group (if more than 1 Group listed in the byline) and/or Subgroup (eg, Steering Committee)
Carolyn	Sue			Neuroscience Research Australia	Sydney, Australia	Data Contributor	GP2
Victor Flores	Ocampo			QIMR Berghofer Medical Research Institute	Brisbane, Australia	Data Contributor	GP2
Christine	Wools			Epworth hospital	Melbourne, Australia	Data Contributor	GP2
Keren Aliza	Weiss			Garvan Institute of Medical Research	Sydney, Australia	Data Contributor	GP2
Ryan L	Davis			University of Sydney	Sydney, Australia	Data Contributor	GP2
Amanda	Willis			Garvan Institute of Medical Research	Sydney, Australia	Data Contributor	GP2
Steven	He			Garvan Institute of Medical Research	Sydney, Australia	Data Contributor	GP2
Robert Arthur	Wilcox			Flinders Medical Centre	Bedford Park, Australia	Data Contributor	GP2
Denise	Howting			Perron Institute for Neurological and Translational Science	Nedlands, Australia	Data Contributor	GP2
Jack David	Price			Perron Institute	Perth, Australia	Data Contributor	GP2
Pak Leng	Cheong			Sydney Local Health District	Sydney, Australia	Data Contributor	GP2
Michel	Tchan			Westmead Hospital	Westmead, Australia	Data Contributor	GP2
Mary-Anne	Young			MonoPD	Sydney, Australia	Data Contributor	GP2
Catriona	Mclean			Florey neuroscience	Melbourne, Australia	Data Contributor	GP2
Nicholas G.	Martin			QIMR Berghofer Medical Research Institute	Brisbane, Australia	Data Contributor	GP2
Hugo Morales	Briceño			Westmead Hospital	Sydney, Australia	Data Contributor	GP2
Thomas	Kimber			Central Adelaide Local Health Network	Adelaide, Australia	Data Contributor	GP2
Kathy H. C.	Wu			St Vincent's Hospital Sydney	Darlinghurst, Australia	Data Contributor	GP2
John	O'Sullivan			University of Queensland	Brisbane, Australia	Data Contributor	GP2
Lewis M	Singleton			Perron Institute of Neurological and Translational Science	Perth, Australia	Data Contributor	GP2

Supplemental Online Content: Nonauthor Collaborators
*First name, last name, and suffix (if applicable) are required and will appear in PubMed.

***First Name and Middle Initial(s)**	***Last Name**	***Suffix (eg, Jr, III)**	Academic Degrees	Institution	Location (city, state/province, country)	Role or Contribution, eg, chair, principal investigator	Group (if more than 1 Group listed in the byline) and/or Subgroup (eg, Steering Committee)
Laura Ivete	Rudaks			Concord Repatriation General Hospital	Sydney, Australia	Data Contributor	GP2
Luis M.	García-Marín			Queensland Institute of Medical Research (QIMR Berghofer)	Brisbane, Australia	Data Contributor	GP2
Amanda	Lim			Queensland Institute of Medical Research (QIMR Berghofer)	Brisbane, Australia	Data Contributor	GP2
Sue-Faye	Siow			Garvan Institute of Medical Research	Sydney, Australia	Data Contributor	GP2
Natalia Soledad	Ogonowski			QIMR Berghofer	Brisbane, Australia	Data Contributor	GP2
Santiago Diaz	Torres			Queensland Institute of Medical Research (QIMR Berghofer) / The University of Queensland	Brisbane, Australia	Data Contributor	GP2
Fangyuan	Cao			QIMR Berghofer	Brisbane, Australia	Data Contributor	GP2
Alexander	Zimprich			Medical University Vienna Austria	Vienna, Austria	Data Contributor	GP2
Kanan	Jafarov			Istanbul Klinik	Baku, Azerbaijan	Data Contributor	GP2
Imran	Sarker			National Institute of Neurosciences and Hospital	Dhaka, Bangladesh	Data Contributor	GP2
David	Crosiers			University of Antwerp	Antwerp, Belgium	Data Contributor	GP2
Erick Gonzalez	Delgado			MedicalCenter Neurocenter S.A.	Santa Cruz de la Sierra, Bolivia	Data Contributor	GP2
Artur F.	Schumacher-Schuh			Universidade Federal do Rio Grande do Sul / Hospital de Clínicas de Porto Alegre	Porto Alegre, Brazil	Data Contributor	GP2
Carlos	Rieder			Federal University of Health Sciences of Porto Alegre	Porto Alegre, Brazil	Data Contributor	GP2
Paula Saffie	Awad			Universidade Federal do Rio Grande do Sul	Porto Alegre, Brazil	Data Contributor	GP2

*First name, last name, and suffix (if applicable) are required and will appear in PubMed.

***First Name and Middle Initial(s)**	***Last Name**	***Suffix (eg, Jr, III)**	Academic Degrees	Institution	Location (city, state/province, country)	Role or Contribution, eg, chair, principal investigator	Group (if more than 1 Group listed in the byline) and/or Subgroup (eg, Steering Committee)
Vitor	Tumas			University of São Paulo	São Paulo, Brazil	Data Contributor	GP2
Sarah	Camargos			Universidade Federal de Minas Gerais	Belo Horizonte, Brazil	Data Contributor	GP2
Lucas Faria	Costa			Universidade Federal de Minas Gerais	Belo Horizonte, Brazil	Data Contributor	GP2
Pedro Braga	Neto			Federal University of Ceará	Fortaleza, Brazil	Data Contributor	GP2
Juan Sebastián Sánchez	León			Hospital de Clínicas de Porto Alegre	Porto Alegre, Brazil	Data Contributor	GP2
Maira Rozenfeld	Olchik			Hospital de Clínicas de Porto Alegre (HCPA)	Porto Alegre, Brazil	Data Contributor	GP2
Bruno Lopes	Santos-Lobato			Hospital Ophir Loyola	Belém, Brazil	Data Contributor	GP2
Grace Helena	Letro			Hospital da Pontificia Universiade Católica de Campinas (Hospital PUC-Campinas	Campinas, Brazil	Data Contributor	GP2
Daniel	Teixeira-dos-Santos			Hospital de Clinicas de Porto Alegre	Porto Alegre, Brazil	Data Contributor	GP2
Henrique Ballalai	Ferraz			Universidade Federal de São Paulo (UNIFESP/EPM)	São Paulo, Brazil	Data Contributor	GP2
Carolina Candeias da	Silva			Universidade Federal de São Paulo UNIFESP/EPM	São Paulo, Brazil	Data Contributor	GP2
Vanderci	Borges			Universidade Federal de São Paulo UNIFESP/EPM	São Paulo, Brazil	Data Contributor	GP2
Dayany Leonel	Boone			Universidade Federal de São Paulo - UNIFESP/EPM	São Paulo, Brazil	Data Contributor	GP2
Mariana Cavalcanti	Costa			Universidade Federal de São Paulo - UNIFESP/EPM	São Paulo, Brazil	Data Contributor	GP2
Oury	Monchi			Institut universitaire de gériatrie de Montréal	Montreal, Canada	Data Contributor	GP2
Edward	Fon			McGill University	Montreal, Canada	Data Contributor	GP2

Supplemental Online Content: Nonauthor Collaborators
*First name, last name, and suffix (if applicable) are required and will appear in PubMed.

*First Name and Middle Initial(s)	*Last Name	*Suffix (eg, Jr, III)	Academic Degrees	Institution	Location (city, state/province, country)	Role or Contribution, eg, chair, principal investigator	Group (if more than 1 Group listed in the byline) and/or Subgroup (eg, Steering Committee)
Robert	Thibault			Aligning Science Across Parkinson's	Vancouver, Canada	Data Contributor	GP2
Ziv	Gan-Or			McGill University	Montreal, Canada	Data Contributor	GP2
Meron	Teferra			McGill University	Montreal, Canada	Data Contributor	GP2
Anthony	Lang			University of Toronto	Toronto, Canada	Data Contributor	GP2
Konstantin	Senkevich			McGill University	Montreal, Canada	Data Contributor	GP2
Marcelo	Miranda			Departament of Neurology, Clinica MEDS, Santiago , Chile . Fundación Diagnosis , Santiago , Chile	Santiago, Chile	Data Contributor	GP2
María Leonor	Bustamante			Faculty of Medicine Universidad de Chile	Santiago, Chile	Data Contributor	GP2
Juan Cristobal	Nuñez			Universidad de Chile - Clínica Alemana Santiago	Santiago, Chile	Data Contributor	GP2
Boris	Lucero			Universidad Católica del Maule	Talca, Chile	Data Contributor	GP2
Alicia	Colombo			University of Chile	Santiago, Chile	Data Contributor	GP2
Maria Teresa Muñoz	Personal			Universidad de Chile	Santiago, Chile	Data Contributor	GP2
Eduardo Pérez	Palma			Universidad del Desarrollo	Santiago, Chile	Data Contributor	GP2
Pedro	Chana-Cuevas			Universidad de Santiago de Chile	Santiago, Chile	Data Contributor	GP2
Ana Belen Miranda	Cortes			Fundación Diagnosis	Santiago, Chile	Data Contributor	GP2
María Eugenia Contreras	Pinto			Hospital San Juan de Dios	La serena, Chile	Data Contributor	GP2
Francisca	Canals			Inmov	Santiago, Chile	Data Contributor	GP2
Patricio Alejandro Olguín	Aguilera			Universidad de Chile, Facultad de Medicina	Santiago, Chile	Data Contributor	GP2
Elias	Fernandez-Toledo			University of Concepción	Concepcion, Chile	Data Contributor	GP2

Supplemental Online Content: Nonauthor Collaborators
*First name, last name, and suffix (if applicable) are required and will appear in PubMed.

***First Name and Middle Initial(s)**	***Last Name**	***Suffix (eg, Jr, III)**	Academic Degrees	Institution	Location (city, state/province, country)	Role or Contribution, eg, chair, principal investigator	Group (if more than 1 Group listed in the byline) and/or Subgroup (eg, Steering Committee)
Benjamin Pizarro	Galleguillos			Centro de Imagenología, Hospital Clínico Universidad de Chile	Santiago, Chile	Data Contributor	GP2
Carlos	Hernandez			Universidad del Desarrollo	Santiago, Chile	Data Contributor	GP2
Floria C.	Pancetti			Universidad Católica del Norte	Coquimbo, Chile	Data Contributor	GP2
Juan Cristobal Nuñez	Fuster			Hospital Clínico Unversidad de Chile - Clínica Alemana Santiago	Santiago, Chile	Data Contributor	GP2
José Manuel	Fernández			INMOV	Buenos Aires, Chile	Data Contributor	GP2
Lorena Hudson	Aros			INMOV (Instituto Nacional de Movimientos Anormales)	Buenos Aires, Chile	Data Contributor	GP2
Beisha	Tang			Central South University	Changsha, China	Data Contributor	GP2
Huifang	Shang			West China Hospital Sichuan University	Chengdu, China	Data Contributor	GP2
Jifeng	Guo			Xiangya Hospital	Changsha, China	Data Contributor	GP2
Piu	Chan			Capital Medical University	Beijing, China	Data Contributor	GP2
Wei	Luo			Zhejiang University	Hangzhou, China	Data Contributor	GP2
Zhenhua	Liu			Xiangya Hospital, Central South University	Changsha, China	Data Contributor	GP2
Germaine Hiu-Fai	Chan			Queen Elizabeth Hospital	Kowloon, China	Data Contributor	GP2
Nancy	Ip			The Hong Kong University of Science and Technology	Kowloon, China	Data Contributor	GP2
Nelson Yuk-Fai	Cheung			Queen Elizabeth Hospital	Kowloon, China	Data Contributor	GP2
Phillip	Chan			The Hong Kong University of Science and Technology	Kowloon, China	Data Contributor	GP2
Xiaopu	Zhou			The Hong Kong University of Science and Technology	Kowloon, China	Data Contributor	GP2
Gonzalo	Arboleda			Universidad Nacional de Colombia	Bogotá, Colombia	Data Contributor	GP2

*First name, last name, and suffix (if applicable) are required and will appear in PubMed.

***First Name and Middle Initial(s)**	***Last Name**	***Suffix (eg, Jr, III)**	Academic Degrees	Institution	Location (city, state/province, country)	Role or Contribution, eg, chair, principal investigator	Group (if more than 1 Group listed in the byline) and/or Subgroup (eg, Steering Committee)
Jorge	Orozco			Fundación Valle del Lili	Santiago De Cali, Colombia	Data Contributor	GP2
David Antonio	Pineda-Salazar			GRUPO DE NEUROCIENCIAS DE ANTIOQUIA (GNA)	Medellín, Colombia	Data Contributor	GP2
Beatriz Munoz	Ospina			Fundacion Valle del Lili	Santiago De Cali, Colombia	Data Contributor	GP2
Tatiana	Lopez-Gonzalez			Universidad Nacional de Colombia	Bogotá, Colombia	Data Contributor	GP2
Carlos	Velez-Pardo			Universidad de Antioquia	Medellín, Colombia	Data Contributor	GP2
Marlene	Jimenez-Del-Rio			Universidad de Antioquia	Medellín, Colombia	Data Contributor	GP2
Sonia Moreno	Masmela			Universidad de Antioquia	Medellín, Colombia	Data Contributor	GP2
Valentina Quintana	Pena			Icesi University	Cali, Colombia	Data Contributor	GP2
David Fernando Aguillon	Niño			University of Antioquia	Medellín, Colombia	Data Contributor	GP2
Eliana Pineda	Mateus			Universidad del Rosario	Bogotá, Colombia	Data Contributor	GP2
Karen Lizzette Velasquez	Mendez			Universidad Nacional de Colombia	Bogotá, Colombia	Data Contributor	GP2
Alvaro	Hernandez			University of Costa Rica	San Jose, Costa Rica	Data Contributor	GP2
Jaime Fornaguera	Trías			Universidad de Costa Rica	San José, Costa Rica	Data Contributor	GP2
Roger Rodriguez	Monge			Universidad de Costa Rica	San José, Costa Rica	Data Contributor	GP2
Per	Borghammer			Aarhus University	Aarhus, Denmark	Data Contributor	GP2
Rossy Cruz	Vicioso			Unión Médica Del Norte, Clínica Universitaria	Santiago de los Caballeros, Dominican Republic	Data Contributor	GP2
Alpher Perez	Arias			Clinica Union Medica del Norte	Santiago de los Caballeros, Dominican Republic	Data Contributor	GP2

*First name, last name, and suffix (if applicable) are required and will appear in PubMed.

*First Name and Middle Initial(s)	*Last Name	*Suffix (eg, Jr, III)	Academic Degrees	Institution	Location (city, state/province, country)	Role or Contribution, eg, chair, principal investigator	Group (if more than 1 Group listed in the byline) and/or Subgroup (eg, Steering Committee)
Ernestina Castro	Salazar			Unión Medica del Norte	Santiago de los Caballeros, Dominican Republic	Data Contributor	GP2
Carlos	Rodriguez-Alarcon			Interlab	Guayaquil, Ecuador	Data Contributor	GP2
Mohamed	Salama			The American University in Cairo	Cairo, Egypt	Data Contributor	GP2
Ali	Shalash			Ain Shams University Hospital	Cairo, Egypt	Data Contributor	GP2
Tatiana	Ascencio			Dr. Andres Bello university	San Salvador, El Salvador	Data Contributor	GP2
Oscar	Peña-Rodas			Universidad Dr Andrés Bello	San Salvador, El Salvador	Data Contributor	GP2
Susana Lissette Peña	Martínez			UNAB	San Salvador, El Salvador	Data Contributor	GP2
Yared Z.	Zewde			Addis Ababa University	Addis Ababa, Ethiopia	Data Contributor	GP2
Alexis	Brice			Paris Brain Institute	Paris, France	Data Contributor	GP2
Jean-Christophe	Corvol			Sorbonne Université	Paris, France	Data Contributor	GP2
Mari	Vidailhet			Salpêtriere Hospital (AP-HP), Sorbonne Université	Paris, France	Data Contributor	GP2
Mathieu	Anheim			University Hospital of Strasbourg, Strasbourg, France	STRASBOURG, France	Data Contributor	GP2
Yves	Agid			Paris Brain Institute	Paris, France	Data Contributor	GP2
Louise-Laure	Mariani			Paris Brain Institute - Sorbonne University	Paris, France	Data Contributor	GP2
Alexandra	Durr			Paris Brain Institute	Paris, France	Data Contributor	GP2
NA	NA			université Toulouse	toulouse, France	Data Contributor	GP2
Ory Magne	Fabienne			chu toulouse	toulouse, France	Data Contributor	GP2
Suzanne	Lesage			Paris Brain Institute (ICM)	Paris, France	Data Contributor	GP2
Defebvre	Luc			CHU Lille	Lille, France	Data Contributor	GP2
Tesson	Christelle			Institut du Cerveau-Paris Brain Institute-ICM	Paris, France	Data Contributor	GP2

Supplemental Online Content: Nonauthor Collaborators
*First name, last name, and suffix (if applicable) are required and will appear in PubMed.

***First Name and Middle Initial(s)**	***Last Name**	***Suffix (eg, Jr, III)**	Academic Degrees	Institution	Location (city, state/province, country)	Role or Contribution, eg, chair, principal investigator	Group (if more than 1 Group listed in the byline) and/or Subgroup (eg, Steering Committee)
Philippe	Damier			Nantes Université	Nantes, France	Data Contributor	GP2
François	Tison			University of Bordeaux, France	Bordeaux, France	Data Contributor	GP2
Stéphane	Thobois			Hospices civils de Lyon, Hopital Neurologique Pierre Wertheimer	BRON, France	Data Contributor	GP2
Jean-Luc	Houeto			Limoges University Hospital	Limoges, France	Data Contributor	GP2
Brefel Courbon	Christine			CHU Toulouse	TOULOUSE, France	Data Contributor	GP2
Sara	Sambin			CIC Neurosciences ,Paris Brain institute	Paris, France	Data Contributor	GP2
Aymeric	Lanore			Paris Brain Institute	Paris, France	Data Contributor	GP2
NS-PARK	Consortium			/	/, France	Data Contributor	GP2
Isabelle	Arnulf			Hopital Universitaire Pitie Salpetriere, AP-HP	Paris, France	Data Contributor	GP2
Stéphane	Lehericy			Hopital Universitaire Pitie Salpetriere, AP-HP	Paris, France	Data Contributor	GP2
Graziella	Mangone			Hopital Universitaire Pitie Salpetriere, AP-HP	Paris, France	Data Contributor	GP2
Poornima	Menon			Hopital Universitaire Pitie Salpetriere, AP-HP	Paris, France	Data Contributor	GP2
David	Grabli			Hopital Universitaire Pitie Salpetriere, AP-HP	Paris, France	Data Contributor	GP2
Florence	Cormier-Dequaire			Hopital Universitaire Pitie Salpetriere, AP-HP	Paris, France	Data Contributor	GP2
Bertrand	Degos			Avicenne Hospital	Bobigny, France	Data Contributor	GP2
NA	NA			Hopital Universitaire Pitie Salpetriere, AP-HP	Paris, France	Data Contributor	GP2
Eve	Benchetrit			La Timone Hospital	Marseille, France	Data Contributor	GP2
Anais	Raud			Centre de réadaptation de l’Estuaire & Résidence les jardines de la chenaie	Le Havre, France	Data Contributor	GP2

*First name, last name, and suffix (if applicable) are required and will appear in PubMed.

*First Name and Middle Initial(s)	*Last Name	*Suffix (eg, Jr, III)	Academic Degrees	Institution	Location (city, state/province, country)	Role or Contribution, eg, chair, principal investigator	Group (if more than 1 Group listed in the byline) and/or Subgroup (eg, Steering Committee)
Virginie	Czernecki			Hopital Universitaire Pitie Salpetriere, AP-HP	Paris, France	Data Contributor	GP2
Pierre	Pouget			ICM	Paris, France	Data Contributor	GP2
Tymothée	Poitou			ICM	Paris, France	Data Contributor	GP2
Elodie	Hainque			Hopital Universitaire Pitie Salpetriere, AP-HP	Paris, France	Data Contributor	GP2
Smaranda Leu	Semenescu			Hopital Universitaire Pitie Salpetriere, AP-HP	Paris, France	Data Contributor	GP2
Pauline	Dodet			Hopital Universitaire Pitie Salpetriere, AP-HP	Paris, France	Data Contributor	GP2
Samir	Bekadar			Eli Lilly and Company	Paris, France	Data Contributor	GP2
Fanny	Mochel			Hopital Universitaire Pitie Salpetriere, AP-HP	Paris, France	Data Contributor	GP2
Farid	Ichou			Hopital Universitaire Pitie Salpetriere, AP-HP	Paris, France	Data Contributor	GP2
Rahul	Gaurav			Hopital Universitaire Pitie Salpetriere, AP-HP	Paris, France	Data Contributor	GP2
Nadya	Pyatigorskaya			Hopital Universitaire Pitie Salpetriere, AP-HP	Paris, France	Data Contributor	GP2
Romain	Valabregue			ICM	Paris, France	Data Contributor	GP2
Cécile	Galléa			ICM	Paris, France	Data Contributor	GP2
Marie-Odile	Habert			Hopital Universitaire Pitie Salpetriere, AP-HP	Paris, France	Data Contributor	GP2
Dijana	Petrovska			Telecom Sud Paris, Evry	Évry, France	Data Contributor	GP2
Laetitia	Jeancolas			Telecom Sud Paris, Evry / ICM	Évry, France	Data Contributor	GP2
Manon	Gomes			Hopital Universitaire Pitie Salpetriere, AP-HP	Paris, France	Data Contributor	GP2
Stephanie	Carvalho			Hopital Universitaire Pitie Salpetriere, AP-HP	Paris, France	Data Contributor	GP2
Alizé	Chalançon			Hopital Universitaire Pitie Salpetriere, AP-HP	Paris, France	Data Contributor	GP2

*First name, last name, and suffix (if applicable) are required and will appear in PubMed.

***First Name and Middle Initial(s)**	***Last Name**	***Suffix (eg, Jr, III)**	Academic Degrees	Institution	Location (city, state/province, country)	Role or Contribution, eg, chair, principal investigator	Group (if more than 1 Group listed in the byline) and/or Subgroup (eg, Steering Committee)
Carole	Dongmo-Kenfack			Hopital Universitaire Pitie Salpetriere, AP-HP	Paris, France	Data Contributor	GP2
Mickael	Le			Hopital Universitaire Pitie Salpetriere, AP-HP	Paris, France	Data Contributor	GP2
Amelie	Bernardo			Hopital Universitaire Pitie Salpetriere, AP-HP	Paris, France	Data Contributor	GP2
Avigaelle	Abitbol			Hopital Universitaire Pitie Salpetriere, AP-HP	Paris, France	Data Contributor	GP2
Marion	Houot			Hopital Universitaire Pitie Salpetriere, AP-HP	Paris, France	Data Contributor	GP2
Sylvie	Forlani			Hopital Universitaire Pitie Salpetriere, AP-HP	Paris, France	Data Contributor	GP2
Sophia	Loiodice			Hopital Universitaire Pitie Salpetriere, AP-HP	Paris, France	Data Contributor	GP2
Ludmila	Jornea			Hopital Universitaire Pitie Salpetriere, AP-HP	Paris, France	Data Contributor	GP2
Mariam	Kekenadze			Tbilisi State Medical University	Tbilisi, Georgia	Data Contributor	GP2
Irine	Khatiashvili			S. Khechinashvili University Hospital	Tbilisi, Georgia	Data Contributor	GP2
Maia	Beridze			Tbilisi State Medical University	Tbilisi, Georgia	Data Contributor	GP2
Sophia	Sopromadze			Ivane Javakhishvili Tbilisi State University	Tbilisi, Georgia	Data Contributor	GP2
Irine	Khatiashvilli			Ivane Javakhishvili Tbilisi State University	Tbilisi, Georgia	Data Contributor	GP2
Mariam	Mshvenieradze			Ivane Javakhishvili Tbilisi State University	Tbilisi, Georgia	Data Contributor	GP2
Marika	Megrelishvili			Ilia State University	Tbilisi, Georgia	Data Contributor	GP2
Alexander	Tsiskaridze			Ivane Javakhishvili Tbilisi State University	Tbilisi, Georgia	Data Contributor	GP2
Ana	Westenberger			University of Lübeck	Lübeck, Germany	Data Contributor	GP2

Supplemental Online Content: Nonauthor Collaborators
*First name, last name, and suffix (if applicable) are required and will appear in PubMed.

*First Name and Middle Initial(s)	*Last Name	*Suffix (eg, Jr, III)	Academic Degrees	Institution	Location (city, state/province, country)	Role or Contribution, eg, chair, principal investigator	Group (if more than 1 Group listed in the byline) and/or Subgroup (eg, Steering Committee)
Anastasia	Illarionova			Deutsches Zentrum für Neurodegenerative Erkrankungen	Göttingen, Germany	Data Contributor	GP2
Brit	Mollenhauer			University Medical Center Göttingen	Göttingen, Germany	Data Contributor	GP2
Christine	Klein			University of Lübeck	Lübeck, Germany	Data Contributor	GP2
Eva-Juliane	Vollstedt			University of Lübeck	Lübeck, Germany	Data Contributor	GP2
Franziska	Hopfner			Department of Neurology, University Hospital, LMU Munich	Munich, Germany	Data Contributor	GP2
Günter	Höglinger			Department of Neurology, University Hospital, LMU Munich	Munich, Germany	Data Contributor	GP2
Harutyun	Madoev			University of Lübeck	Lübeck, Germany	Data Contributor	GP2
Joanne	Trinh			University of Lübeck	Lübeck, Germany	Data Contributor	GP2
Katja	Lohmann			University of Lübeck	Lübeck, Germany	Data Contributor	GP2
Manu	Sharma			University of Tubingen	Tübingen, Germany	Data Contributor	GP2
Sergiu	Groppa			University of Mainz	Mainz, Germany	Data Contributor	GP2
Thomas	Gasser			University of Tübingen	Tübingen, Germany	Data Contributor	GP2
Zih-Hua	Fang			The German Center for Neurodegenerative Diseases	Göttingen, Germany	Data Contributor	GP2
Karl	Heilbron			Charité - Universitätsmedizin Berlin	Berlin, Germany	Data Contributor	GP2
Wenhua	Sun			University of Teubingen	Tübingen, Germany	Data Contributor	GP2
Inke	König			University of Lübeck	Lübeck, Germany	Data Contributor	GP2
Daniela	Berg			University Medical Center Schleswig-Holstein	Lübeck, Germany	Data Contributor	GP2
Bernhard	Haslinger			Technical University of Munich	Munich, Germany	Data Contributor	GP2
Teresa	Kleinz			University of Lübeck	Lübeck, Germany	Data Contributor	GP2
Norbert	Brüggemann			University of Lübeck	Lübeck, Germany	Data Contributor	GP2

Supplemental Online Content: Nonauthor Collaborators

*First name, last name, and suffix (if applicable) are required and will appear in PubMed.

***First Name and Middle Initial(s)**	***Last Name**	***Suffix (eg, Jr, III)**	Academic Degrees	Institution	Location (city, state/province, country)	Role or Contribution, eg, chair, principal investigator	Group (if more than 1 Group listed in the byline) and/or Subgroup (eg, Steering Committee)
Konstantin	Kufer			German Centre for Neurodegenerative Diseases (DZNE) / University Hospital Bonn	Bonn, Germany	Data Contributor	GP2
Antonia Maria	Buchal			University Hospital Bonn	Bonn, Germany	Data Contributor	GP2
Matthias	Höllerhage			Hannover Medical School	Hannover, Germany	Data Contributor	GP2
Florian	Wegner			Hannover Medical School	Hannover, Germany	Data Contributor	GP2
Nils	Schroeter			UKS, University of Saarland	Homburg and Mainz, Germany	Data Contributor	GP2
Kathrin	Brockmann			University of Tübingen	Tübingen, Germany	Data Contributor	GP2
Isabel	Wurster			University of Tübingen	Tübingen, Germany	Data Contributor	GP2
Theresa	Lüth			University of Lübeck	Lübeck, Germany	Data Contributor	GP2
Christian	Beetz			CENTOGENE	Rostock, Germany	Data Contributor	GP2
Krishnakumar	Kandaswamy			Centogene GmbH	Berlin, Germany	Data Contributor	GP2
Eva	Schäffer			Kiel University	Kiel, Germany	Data Contributor	GP2
Kirsten	Zeuner			Kiel University	Kiel, Germany	Data Contributor	GP2
Gregor	Kuhlenbäumer			Kiel University	Kiel, Germany	Data Contributor	GP2
Peter	Bauer			Centogene GmbH	Rostock, Germany	Data Contributor	GP2
Martin	Klietz			Hannover Medical School	Hannover, Germany	Data Contributor	GP2
Carolin	Gabbert			University of Lübeck	Lübeck, Germany	Data Contributor	GP2
Alexander	Balck			University of Lübeck	Lübeck, Germany	Data Contributor	GP2
Christoph	Westenberger			University of Lübeck	Lübeck, Germany	Data Contributor	GP2
Claudia	Schulte			University of Tübingen	Tübingen, Germany	Data Contributor	GP2
Sebastian	Schade			Paracelsus-Elena-Klinik Kassel	Kassel, Germany	Data Contributor	GP2
Michael	Sommerauer			University Hospital Bonn	Bonn, Germany	Data Contributor	GP2
Andre	Fienemann			University of Lübeck	Lübeck, Germany	Data Contributor	GP2
Ann-Kathrin	Hauser			University of Tübingen	Tübingen, Germany	Data Contributor	GP2
Claudia	Schulte			University of Tübingen	Tübingen, Germany	Data Contributor	GP2
Sana	Hrir			University of Lübeck	Lübeck, Germany	Data Contributor	GP2

Supplemental Online Content: Nonauthor Collaborators
*First name, last name, and suffix (if applicable) are required and will appear in PubMed.

*First Name and Middle Initial(s)	*Last Name	*Suffix (eg, Jr, III)	Academic Degrees	Institution	Location (city, state/province, country)	Role or Contribution, eg, chair, principal investigator	Group (if more than 1 Group listed in the byline) and/or Subgroup (eg, Steering Committee)
Albert	Akpalu			University of Ghana Medical School	Accra, Ghana	Data Contributor	GP2
Momodou	Cham			Richard Novati Catholic Hospital, Catholic Health Service Trust	Accra, Ghana	Data Contributor	GP2
Vida	Obese			Kwame Nkrumah University of Science and Technology	Kumasi, Ghana	Data Contributor	GP2
Andrew Jacobs	Bilson			Kwame Nkrumah University of Science and Technology	Kumasi, Ghana	Data Contributor	GP2
Georgia	Xiromerisiou			University of Thessaly	Volos, Greece	Data Contributor	GP2
Georgios	Hadjigorgiou			University of Thessaly	Volos, Greece	Data Contributor	GP2
Ioannis	Dagklis			Aristotle University of Thessaloniki	Thessaloniki, Greece	Data Contributor	GP2
Ioannis	Tarnanas			Ionian University	Corfu, Greece	Data Contributor	GP2
Leonidas	Stefanis			Biomedical research Foundation of the Academy of Athens	Athens, Greece	Data Contributor	GP2
Maria	Stamelou			Diagnostic and Therapeutic Centre HYGEIA Hospital	Marousi, Greece	Data Contributor	GP2
Efthymios	Dadiotis			University of Thessaly	Volos, Greece	Data Contributor	GP2
Tsamis	Konstantinos			University of Ioannina	Ioannina, Greece	Data Contributor	GP2
Konitsiotis	Spyridon			University of Ioannina	Ioannina, Greece	Data Contributor	GP2
Iro	Boura			University of Crete	Heraklion, Greece	Data Contributor	GP2
Makrygianni	Mariza			HYGEIA Hospital	Athens, Greece	Data Contributor	GP2
Lina	Florentin			HYGEIA Hospital	Athens, Greece	Data Contributor	GP2
Maria	Makrygianni			HYGEIA Hospital	Athens, Greece	Data Contributor	GP2
Foivos S.	Kanellos			University of Ioannina	Ioannina, Greece	Data Contributor	GP2
Cleanthe	Spanaki			University of Crete	Heraklion, Greece	Data Contributor	GP2
Alex	Medina			Hospital San Felipe	Tegucigalpa, Honduras	Data Contributor	GP2
Evelin Álvarez	Herrera			Universidad Tecnológica Centroamericana (UNITEC)	Tegucigalpa, Honduras	Data Contributor	GP2

***First Name and Middle Initial(s)**	***Last Name**	***Suffix (eg, Jr, III)**	Academic Degrees	Institution	Location (city, state/province, country)	Role or Contribution, eg, chair, principal investigator	Group (if more than 1 Group listed in the byline) and/or Subgroup (eg, Steering Committee)
Heike Hesse	Joya			Universidad Tecnológica Centroamericana UNITEC	Tegucigalpa, Honduras	Data Contributor	GP2
Reyna M.	Durón			Universidad Tecnológica Centroamericana	Tegucigalpa, Honduras	Data Contributor	GP2
Glenda Oliva	Fuentes			Fundación Lucas para la Salud	Tegucigalpa, Honduras	Data Contributor	GP2
Eduardo Jose Ponce	Murillo			Universidad Tecnológica Centroamericana	Tegucigalpa, Honduras	Data Contributor	GP2
Kari	Stefansson			deCODE genetics/Amgen Inc., Reykjavik, Iceland Faculty of Medicine, University of Iceland, Reykjavik, Iceland	Reykjavik, Iceland	Data Contributor	GP2
Hreinn	Stefansson			deCODE genetics/Amgen Inc., Reykjavik, Iceland Faculty of Medicine, University of Iceland, Reykjavik, Iceland	Reykjavik, Iceland	Data Contributor	GP2
Vala	Palmadottir			deCODE genetics/Amgen Inc., Reykjavik, Iceland Faculty of Medicine, University of Iceland, Reykjavik, Iceland	Reykjavik, Iceland	Data Contributor	GP2
Astros Th.	Skuladottir			deCODE genetics/Amgen Inc., Reykjavik, Iceland Faculty of Medicine, University of Iceland, Reykjavik, Iceland	Reykjavik, Iceland	Data Contributor	GP2
Asha	Kishore			Aster Medcity	Kochi, India	Data Contributor	GP2
Divya	Kp			Sree Chitra Tirunal Institute for Medical Sciences and Technology	Thiruvananthapuram, India	Data Contributor	GP2
Pramod	Pal			National Institute of Mental Health & Neurosciences	Bengaluru, India	Data Contributor	GP2
Prashanth Lingappa	Kukkle			Manipal Hospital	Delhi, India	Data Contributor	GP2

*First name, last name, and suffix (if applicable) are required and will appear in PubMed.

*First Name and Middle Initial(s)	*Last Name	*Suffix (eg, Jr, III)	Academic Degrees	Institution	Location (city, state/province, country)	Role or Contribution, eg, chair, principal investigator	Group (if more than 1 Group listed in the byline) and/or Subgroup (eg, Steering Committee)
Roopa	Rajan			All India Institute of Medical Sciences	Delhi, India	Data Contributor	GP2
Rupam	Borgohain			Nizam's Institute Of Medical Sciences	Hyderabad, India	Data Contributor	GP2
Mehri	Salari			Shahid Beheshti University of Medical Science	Tehran, Iran	Data Contributor	GP2
Tamara	Shiner			Tel Aviv Sourasky Medical Center	Tel Aviv-Yafo, Israel	Data Contributor	GP2
Avner	Thaler			Tel Aviv Sourasky Medical Center	Tel Aviv-Yafo, Israel	Data Contributor	GP2
Noa	Bregman			Tel Aviv Medical Center	Tel Aviv-Yafo, Israel	Data Contributor	GP2
Andrea	Quattrone			Magna Græcia University of Catanzaro	Catanzaro, Italy	Data Contributor	GP2
Enza Maria	Valente			University of Pavia	Pavia, Italy	Data Contributor	GP2
Grazia	Annesi			National Research Council	Cosenza, Italy	Data Contributor	GP2
Lucilla	Parnetti			University of Perugia	Perugia, Italy	Data Contributor	GP2
Micol	Avenali			University of Pavia	Pavia, Italy	Data Contributor	GP2
Monica	Gagliardi			Magna Graecia University	Catanzaro, Italy	Data Contributor	GP2
Tommaso	Schirinzi			University of Rome Tor Vergata	Rome, Italy	Data Contributor	GP2
Caterina	Galandra			IRCCS Mondino Foundation	Pavia, Italy	Data Contributor	GP2
Anna De	Rosa			University of Naples Federico II	Naples, Italy	Data Contributor	GP2
Rosangela	Ferese			IRCCS Neuromed	Pozzilli, Italy	Data Contributor	GP2
Jolanda	Buonocore			Magna Graecia University	Catanzaro, Italy	Data Contributor	GP2
Radha	Procopio			Magna Graecia University	Catanzaro, Italy	Data Contributor	GP2
Ilaria	Palmieri			IRCCS Mondino Foundation	Pavia, Italy	Data Contributor	GP2
Michele	Terzaghi			University of Pavia	Pavia, Italy	Data Contributor	GP2
Paola	Dimartino			University of Pavia	Pavia, Italy	Data Contributor	GP2
Roberta	Bovenzi			University of Tor Vergata	Rome, Italy	Data Contributor	GP2
Maxine	Paige-Pritchett			Università di Pavia	Pavia, Italy	Data Contributor	GP2
Manabu	Funayama			Juntendo University	Tokyo, Japan	Data Contributor	GP2

Supplemental Online Content: Nonauthor Collaborators
*First name, last name, and suffix (if applicable) are required and will appear in PubMed.

***First Name and Middle Initial(s)**	***Last Name**	***Suffix (eg, Jr, III)**	Academic Degrees	Institution	Location (city, state/province, country)	Role or Contribution, eg, chair, principal investigator	Group (if more than 1 Group listed in the byline) and/or Subgroup (eg, Steering Committee)
Nobutaka	Hattori			Juntendo University faculty of medicine	Tokyo, Japan	Data Contributor	GP2
Tomotaka	Shiraishi			Jikei University School of Medicine	Tokyo, Japan	Data Contributor	GP2
Kensuke	Daida			Juntendo University	Bunkyō, Japan	Data Contributor	GP2
Altynay	Karimova			Institute of Neurology and Neurorehabilitation	Almaty, Kazakhstan	Data Contributor	GP2
Gulnaz	Kaishibayeva			Institute of Neurology and Neurorehabilitation	Almaty, Kazakhstan	Data Contributor	GP2
Aigerim	Utegenova			West Kazakhstan Marat Ospanov State Medical University	Aktobe, Kazakhstan	Data Contributor	GP2
Vadim	Akhmetzhanov			Medline medical center	Astana, Kazakhstan	Data Contributor	GP2
Seitzhan	Aidarov			National Center for Neurosurgery	Astana, Kazakhstan	Data Contributor	GP2
Tautanova	Raushan			Astana Medical University	Astana, Kazakhstan	Data Contributor	GP2
Dinara	Alzhanova			Astana Medical University	Astana, Kazakhstan	Data Contributor	GP2
Zhanybek	Myrzayev			International University of Postgraduate Education	Almaty, Kazakhstan	Data Contributor	GP2
Saltanat	Abdraimova			South Kazakhstan Medical Academy	Shymkent, Kazakhstan	Data Contributor	GP2
Nazira	Zharkinbekova			South Kazakhstan Medical Academy	Shymkent, Kazakhstan	Data Contributor	GP2
Chingiz	Shashkin			International Research Institute of Postgraduate Education	Almaty, Kazakhstan	Data Contributor	GP2
Guzel	Shiderova			Institute of Neurology and Neurorehabilitation	Almaty, Kazakhstan	Data Contributor	GP2
Bagzhan	Syzdykova			Astana Medical University	Astana, Kazakhstan	Data Contributor	GP2
Aigul. P.	Yermagambetova			West Kazakhstan Marat Ospanov Medical University	Aktobe, Kazakhstan	Data Contributor	GP2

*First name, last name, and suffix (if applicable) are required and will appear in PubMed.

*First Name and Middle Initial(s)	*Last Name	*Suffix (eg, Jr, III)	Academic Degrees	Institution	Location (city, state/province, country)	Role or Contribution, eg, chair, principal investigator	Group (if more than 1 Group listed in the byline) and/or Subgroup (eg, Steering Committee)
Alima A.	Khamidulla			West Kazakhstan Marat Ospanov Medical University	Aktobe, Kazakhstan	Data Contributor	GP2
Zhanylsyn	U.Urasheva			West Kazakhstan Marat Ospanov Medical University	Aktobe, Kazakhstan	Data Contributor	GP2
Gulnar B.	Kabdrakhmanova			West Kazakhstan Marat Ospanov Medical University	Aktobe, Kazakhstan	Data Contributor	GP2
Talgat	Khaibullin			Semey Medical University	Semey, Kazakhstan	Data Contributor	GP2
Altynay	Talgatkyzy			Semey Medical University	Semey, Kazakhstan	Data Contributor	GP2
Akper	Sagynysh			Multiple Sclerosis and Autoimmune Neurological Disorders Center at the Multidisciplinary City Hospital No. 1	Moscow, Kazakhstan	Data Contributor	GP2
Sadenova	Aigul			Multidisciplinary City Hospital No. 1	Moscow, Kazakhstan	Data Contributor	GP2
Eunice	Nyambane			Aga Khan University Nairobi	Nairobi, Kenya	Data Contributor	GP2
Cholpon	Shambetova			Kyrgyz State Medical Academy	Bishkek, Kyrgyzstan	Data Contributor	GP2
Bermet	Nurbekova			I.K. Akhunbaev Kyrgyz State Medical Academy (KSMA)	Bishkek, Kyrgyzstan	Data Contributor	GP2
Nurayim	Beishembieva			I.K. Akhunbaev Kyrgyz State Medical Academy (KSMA)	Bishkek, Kyrgyzstan	Data Contributor	GP2
Nargiza	Atambekova			I.K. Akhunbaev Kyrgyz State Medical Academy (KSMA), Clinical Hospital of Emergency Medical Care	Bishkek, Kyrgyzstan	Data Contributor	GP2
Zhumagul	Osmonova			Kyrgyz State Medical Institute of postgraduate training and continuous education named after S.B. Daniyarov	Bishkek, Kyrgyzstan	Data Contributor	GP2
Maatali Abdimanap	uulu			Osh State University, Osh City Clinical Hospital	Osh, Kyrgyzstan	Data Contributor	GP2

*First name, last name, and suffix (if applicable) are required and will appear in PubMed.

***First Name and Middle Initial(s)**	***Last Name**	***Suffix (eg, Jr, III)**	Academic Degrees	Institution	Location (city, state/province, country)	Role or Contribution, eg, chair, principal investigator	Group (if more than 1 Group listed in the byline) and/or Subgroup (eg, Steering Committee)
Aisuikum	Abdumalikova			I.K. Akhunbaev Kyrgyz State Medical Academy (KSMA), Osh City Clinical Hospital	Osh, Kyrgyzstan	Data Contributor	GP2
Adylbek	Iusupov			I.K. Akhunbaev Kyrgyz State Medical Academy (KSMA), Osh City Clinical Hospital	Osh, Kyrgyzstan	Data Contributor	GP2
Aliia	Mukhanova			International Higher School of Medicine	Bishkek, Kyrgyzstan	Data Contributor	GP2
Zhyldyz	Imanalieva			I.K. Akhunbaev Kyrgyz State Medical Academy (KSMA), "Aqua Lab" LLC	Bishkek, Kyrgyzstan	Data Contributor	GP2
Gulmira	Zhamilova			Clinical Hospital of the Presidential Administration of the Kyrgyz Republic	Bishkek, Kyrgyzstan	Data Contributor	GP2
Shokhista	Artyshova			Clinical Hospital of the Presidential Administration of the Kyrgyz Republic	Bishkek, Kyrgyzstan	Data Contributor	GP2
Sabina	Baltabaeva			I.K. Akhunbaev Kyrgyz State Medical Academy (KSMA), Medcenter.kg" clinic	Bishkek, Kyrgyzstan	Data Contributor	GP2
Mariia	Rekaeva			I.K. Akhunbaev Kyrgyz State Medical Academy (KSMA), "Unimed" Clinic	Bishkek, Kyrgyzstan	Data Contributor	GP2
Aziza	Dalbaeva			National Hospital	Bishkek, Kyrgyzstan	Data Contributor	GP2
Anna	Khamzina			I.K. Akhunbaev Kyrgyz State Medical Academy (KSMA), "Unimed" Clinic	Bishkek, Kyrgyzstan	Data Contributor	GP2
Saule	Temirbaeva			National Hospital	Bishkek, Kyrgyzstan	Data Contributor	GP2
Mukhammadiusuf	Abdykadyrov			Osh State University	Osh, Kyrgyzstan	Data Contributor	GP2

Supplemental Online Content: Nonauthor Collaborators
*First name, last name, and suffix (if applicable) are required and will appear in PubMed.

*First Name and Middle Initial(s)	*Last Name	*Suffix (eg, Jr, III)	Academic Degrees	Institution	Location (city, state/province, country)	Role or Contribution, eg, chair, principal investigator	Group (if more than 1 Group listed in the byline) and/or Subgroup (eg, Steering Committee)
Zynan Ruslan	uulu			I.K. Akhunbaev Kyrgyz State Medical Academy (KSMA)	Bishkek, Kyrgyzstan	Data Contributor	GP2
Nurbakyt	Kadyrov			Osh City Clinical Hospital	Osh, Kyrgyzstan	Data Contributor	GP2
Batma	Sattarova			Batken Regional Hospital	Batken, Kyrgyzstan	Data Contributor	GP2
Begimai	Sovetbekova			I.K. Akhunbaev Kyrgyz State Medical Academy (KSMA)	Bishkek, Kyrgyzstan	Data Contributor	GP2
Fouad	Khoury			Université Saint George - Hôpital national de neurologie et de neurochirurgie	Beirut, Lebanon	Data Contributor	GP2
Gintare	Baranauskiene			Vilnius University	Vilnius, Lithuania	Data Contributor	GP2
Rejko	Krüger			University of Luxembourg	Esch-sur-Alzette, Luxembourg	Data Contributor	GP2
Patrick	May			University of Luxembourg	Esch-sur-Alzette, Luxembourg	Data Contributor	GP2
Nomena	Rasaholiarison			Faculty of Medicine, University of Fianarantsoa	Fianarantsoa, Madagascar	Data Contributor	GP2
Ai Huey	Tan			University of Malaya	Kuala Lumpur, Malaysia	Data Contributor	GP2
Azlina	Ahmad-Annuar			University of Malaya	Kuala Lumpur, Malaysia	Data Contributor	GP2
Mohamed Ibrahim	Norlinah			Universiti Kebangsaan Malaysia	Selangor, Malaysia	Data Contributor	GP2
Nor Azian Abdul	Murad			UKM Medical Molecular Biology Institute	Kuala Lumpur, Malaysia	Data Contributor	GP2
Shahrul	Azmin			Universiti Kebangsaan Malaysia Medical Centre	Kuala Lumpur, Malaysia	Data Contributor	GP2
Shen-Yang	Lim			University of Malaya	Kuala Lumpur, Malaysia	Data Contributor	GP2
Wael	Mohamed			International Islamic University	Kuala Lumpur, Malaysia	Data Contributor	GP2

Supplemental Online Content: Nonauthor Collaborators
*First name, last name, and suffix (if applicable) are required and will appear in PubMed.

***First Name and Middle Initial(s)**	***Last Name**	***Suffix (eg, Jr, III)**	Academic Degrees	Institution	Location (city, state/province, country)	Role or Contribution, eg, chair, principal investigator	Group (if more than 1 Group listed in the byline) and/or Subgroup (eg, Steering Committee)
Yi Wen	Tay			University of Malaya	Kuala Lumpur, Malaysia	Data Contributor	GP2
Lim	Kai-Shi			University of Malaya	Kuala Lumpur, Malaysia	Data Contributor	GP2
Azalea Tenerife	Pajo			University of Malaya	Kuala Lumpur, Malaysia	Data Contributor	GP2
Chia Yuen	Kang			Hospital Queen Elizabeth	Kota Kinabalu, Malaysia	Data Contributor	GP2
Joshua Ooi Chin	Ern			Queen Elizabeth Hospital	Kota Kinabalu, Malaysia	Data Contributor	GP2
Khairul Azmi	Ibrahim			HOSPITAL SULTANAH NUR ZAHIRAH KUALA TERENGGANU	KUALA TERENGGANU, Malaysia	Data Contributor	GP2
Ahmad Shahir Bin	Mawardi			Hospita Kuala Lumpur	Kuala Lumpur, Malaysia	Data Contributor	GP2
Lim Thien	Thien			Island Hospital	Penang, Malaysia	Data Contributor	GP2
Tzi Shin	Toh			University of Malaya	Kuala Lumpur, Malaysia	Data Contributor	GP2
Daniel	Martinez-Ramirez			Tecnologico de Monterrey	Monterrey, Mexico	Data Contributor	GP2
Paula	Reyes-Pérez			Universidad Nacional Autónoma de México	Santiago de Querétaro, Mexico	Data Contributor	GP2
Alejandra Medina	Rivera			Universidad Nacional Autónoma de México	Santiago de Querétaro, Mexico	Data Contributor	GP2
Nancy Monroy	Jaramillo			Instituto Nacional de Neurología y Neurocirugía	Mexico City, Mexico	Data Contributor	GP2
Nadia Alejandra Gandarilla	Martinez			Centro Neurológico del Centro Médico ABC, Campus Santa Fe	Mexico City, Mexico	Data Contributor	GP2
Ingrid	Estrada-Bellmann			UNIVERSITY HOSPITAL “DR JOSE E GONZALEZ”	Monterrey, Mexico	Data Contributor	GP2
Araliz	Puente			Hospital Ángeles Puebla	Puebla, Mexico	Data Contributor	GP2

*First name, last name, and suffix (if applicable) are required and will appear in PubMed.

***First Name and Middle Initial(s)**	***Last Name**	***Suffix (eg, Jr, III)**	Academic Degrees	Institution	Location (city, state/province, country)	Role or Contribution, eg, chair, principal investigator	Group (if more than 1 Group listed in the byline) and/or Subgroup (eg, Steering Committee)
Ana Paula Angulo	Arrieta			Hospital Ángeles Puebla, Universidad Anáhuac Puebla	Puebla, Mexico	Data Contributor	GP2
Eugenia Morelos	Figaredo			ISSSTE Morelia	Morelia, Mexico	Data Contributor	GP2
Karla Salinas	Barboza			HOSPITAL GENERAL DE MEXICO	Mexico City, Mexico	Data Contributor	GP2
Dante Bernardo Oropeza	Canto			Hospital Angeles de Puebla	Puebla, Mexico	Data Contributor	GP2
Mayela	Rodríguez-Violante			Instituto Nacional de Neurología y Neurocirugía	Mexico City, Mexico	Data Contributor	GP2
Ana Jimena	Hernández-Medrano			Instituto Nacional de Neurología y Neurocirugía Manuel Velasco Suárez	Mexico City, Mexico	Data Contributor	GP2
Amin	Cervantes-Arriaga			Instituto Nacional de Neurología y Neurocirugía	Mexico City, Mexico	Data Contributor	GP2
Edith Janeth Gaspar	Martínez			Universidad Nacional Autónoma de México	Santiago de Querétaro, Mexico	Data Contributor	GP2
Alejandra E-	Ruiz-Contreras			Universidad Nacional Autonoma de Mexico	Mexico City, Mexico	Data Contributor	GP2
Alejandra	Lázaro-Figueroa			National Autonomous University of Mexico	Mexico City, Mexico	Data Contributor	GP2
Sarael	Alcauter			Universidad Nacional Autónoma de México	Mexico City, Mexico	Data Contributor	GP2
Edwin Roberto	Ramírez-Benítez			Instituto Nacional de Neurología y Neurocirugía	Mexico City, Mexico	Data Contributor	GP2
Karina	Cruz-Santillán			Instituto Nacional de Neurología y Neurocirugía	Mexico City, Mexico	Data Contributor	GP2
Rodolfo	Solís-Vivanco			Instituto Nacional de Neurología y Neurocirugía	Mexico City, Mexico	Data Contributor	GP2
Bayasgalan	Tserensodnom			Mongolian National University of Medical Sciences	Ulaanbaatar, Mongolia	Data Contributor	GP2
Khosbayar	Tulgaa			Mongolian National University of Medical Sciences	Ulaanbaatar, Mongolia	Data Contributor	GP2

*First name, last name, and suffix (if applicable) are required and will appear in PubMed.

***First Name and Middle Initial(s)**	***Last Name**	***Suffix (eg, Jr, III)**	Academic Degrees	Institution	Location (city, state/province, country)	Role or Contribution, eg, chair, principal investigator	Group (if more than 1 Group listed in the byline) and/or Subgroup (eg, Steering Committee)
Oyujin	Ulziibaatar			Mongolian National University of Medical Sciences	Ulaanbaatar, Mongolia	Data Contributor	GP2
Ahmed	Bouhouche			Specialties Hospital, CHU Ibn Sina	Rabat, Morocco	Data Contributor	GP2
Mossafa	Hossain			Clinique OCEANIC	CASABLANCA, Morocco	Data Contributor	GP2
Rajeev	Ojha			Tribhuvan University	Kirtipur, Nepal	Data Contributor	GP2
Wilma Van De	Berg			Vanderbilt University Medical Center	Amsterdam, Netherlands	Data Contributor	GP2
Bas	Bleom			Radboud University	Nijmegen, Netherlands	Data Contributor	GP2
Bart Van De	Warrenburg			Radboud University Medical Center	Nijmegen, Netherlands	Data Contributor	GP2
Lisette	Charbonnier			Brain Research and Innovation Center	Amsterdam, Netherlands	Data Contributor	GP2
Tim J.	Anderson			University of Otago	Dunedin, New Zealand	Data Contributor	GP2
Toni L.	Pitcher			University of Otago	Dunedin, New Zealand	Data Contributor	GP2
Daniel Jeremy	Myall			New Zealand Brain Research Institution	Christchurch, New Zealand	Data Contributor	GP2
John C.	Dalrymple-Alford			University of Canterbury	Christchurch, New Zealand	Data Contributor	GP2
Joseph	Donnelly			Te Whatu Ora - Health New Zealand Auckland, New Zealand	Auckland, New Zealand	Data Contributor	GP2
Arinola	Sanyaolu			University of Lagos	Lagos, Nigeria	Data Contributor	GP2
Njideka	Okubadejo			University of Lagos	Lagos, Nigeria	Data Contributor	GP2
Oluwadamilola	Ojo			College of Medicine of the University of Lagos	Lagos, Nigeria	Data Contributor	GP2
Simon Izuchukwu	Ozomma			University of Calabar Teaching Hospital	Calabar, Nigeria	Data Contributor	GP2
Kolawole	Wahab			University of Ilorin	Ilorin, Nigeria	Data Contributor	GP2

Supplemental Online Content: Nonauthor Collaborators
*First name, last name, and suffix (if applicable) are required and will appear in PubMed.

***First Name and Middle Initial(s)**	***Last Name**	***Suffix (eg, Jr, III)**	Academic Degrees	Institution	Location (city, state/province, country)	Role or Contribution, eg, chair, principal investigator	Group (if more than 1 Group listed in the byline) and/or Subgroup (eg, Steering Committee)
Oladunni	Abiodun			General Hospital	Lagos, Nigeria	Data Contributor	GP2
Sani	Abubakar			Ahmadu Bello University	Kaduna State, Nigeria	Data Contributor	GP2
Fatimah	Abdulai			University of Abuja Teaching Hospital	Gwagwalada, Nigeria	Data Contributor	GP2
Charles	Achoru			Jos University Teaching Hospital	Jos, Nigeria	Data Contributor	GP2
Osigwe	Agabi			College of Medicine, University of Lagos	Lagos, Nigeria	Data Contributor	GP2
Uchechi	Agulanna			Lagos University Teaching Hospital	Lagos, Nigeria	Data Contributor	GP2
Rufus	Akinyemi			Neuroscience and Ageing Research Unit, Institute for Advanced Medical Research and Training, College of Medicine, University of Ibadan	Ibadan, Nigeria	Data Contributor	GP2
Wemimo	Alaofin			University of Ilorin	Ilorin, Nigeria	Data Contributor	GP2
Ifeyinwa	Ani-Osheku			Asokoro District Hospital	Abuja, Nigeria	Data Contributor	GP2
Roosevelt	Anyanwu			College of Medicine, University of Lagos	Lagos, Nigeria	Data Contributor	GP2
Cyril	Erameh			Irrua Specialist Teaching Hospital	Ilorin, Nigeria	Data Contributor	GP2
Daniel	Ezuduemoih			Lagos University Teaching Hospital	Lagos, Nigeria	Data Contributor	GP2
Abdullahi	Ibrahim			Federal University of Health Sciences Teaching Hospital	Azare, Nigeria	Data Contributor	GP2
Erica	Ikwenu			Lagos University Teaching Hospital	Lagos, Nigeria	Data Contributor	GP2
Frank	Imarhiagbe			University of Benin	Benin City, Nigeria	Data Contributor	GP2
Ismaila	Ishola			College of Medicine, University of Lagos	Lagos, Nigeria	Data Contributor	GP2
Emmanuel	Iwuozo			Benue State University	Makurdi, Nigeria	Data Contributor	GP2

Supplemental Online Content: Nonauthor Collaborators
*First name, last name, and suffix (if applicable) are required and will appear in PubMed.

*First Name and Middle Initial(s)	*Last Name	*Suffix (eg, Jr, III)	Academic Degrees	Institution	Location (city, state/province, country)	Role or Contribution, eg, chair, principal investigator	Group (if more than 1 Group listed in the byline) and/or Subgroup (eg, Steering Committee)
Morenikeji	Komolafe			Obafemi Awolowo University	Ile-Ife, Nigeria	Data Contributor	GP2
Alero	Nnama			University of Port Harcourt Teaching Hospital	Port Harcourt, Nigeria	Data Contributor	GP2
Paul	Nwani			Nnamdi Azikiwe University Teaching Hospital	Nnewi, Nigeria	Data Contributor	GP2
Franscisca	Nwaokorie			College of Medicine, University of Lagos	Lagos, Nigeria	Data Contributor	GP2
Ernest	Nwazor			Rivers State University Teaching Hospital	Port Harcourt, Nigeria	Data Contributor	GP2
Yahaya	Obiabo			Federal University of Health Sciences	Otukpo, Nigeria	Data Contributor	GP2
Nkechi	Obianozie			University of Abuja Teaching Hospital	Gwagwalada, Nigeria	Data Contributor	GP2
Olanike	Odeniyi			General Hospital	Lagos, Nigeria	Data Contributor	GP2
Francis	Odiase			University of Benin	Benin City, Nigeria	Data Contributor	GP2
Ewere Marie	Ogbimi			Delta State University	Abraka, Nigeria	Data Contributor	GP2
Adebimpe	Ogunmodede			Federal Medical Center	Owo, Nigeria	Data Contributor	GP2
Francis	Ojini			University of Lagos	Lagos, Nigeria	Data Contributor	GP2
Rashidat	Olanigan			Lagos State University Teaching Hospital	Ikeja, Nigeria	Data Contributor	GP2
Adedunni	Olusanya			College of Medicine, University of Lagos & R-Jolad Hospital	Lagos, Nigeria	Data Contributor	GP2
Chiamaka	Okereke			University of Nigeria Teaching Hospital	Ituku-Ozalla, Nigeria	Data Contributor	GP2
Gerald	Onwuegbuzie			University of Abuja	Abuja, Nigeria	Data Contributor	GP2
Godwin	Osaigbovo			Jos University Teaching Hospital	Jos, Nigeria	Data Contributor	GP2
Nosakhare	Osemwegie			University of Port Harcourt	Port Harcourt, Nigeria	Data Contributor	GP2
Olajumoke	Oshinaike			Lagos State University College of Medicine	Ikeja, Nigeria	Data Contributor	GP2
Lukman	Owolabi			Bayero University Kano	Kano, Nigeria	Data Contributor	GP2

Supplemental Online Content: Nonauthor Collaborators
*First name, last name, and suffix (if applicable) are required and will appear in PubMed.

*First Name and Middle Initial(s)	*Last Name	*Suffix (eg, Jr, III)	Academic Degrees	Institution	Location (city, state/province, country)	Role or Contribution, eg, chair, principal investigator	Group (if more than 1 Group listed in the byline) and/or Subgroup (eg, Steering Committee)
Raymond	Owolabi			Federal Medical Center	Owo, Nigeria	Data Contributor	GP2
Shyngle	Oyakhire			National Hospital	Abuja, Nigeria	Data Contributor	GP2
Fadimatu	Sa'Ad			Federal Teaching Hospital	Gombe, Nigeria	Data Contributor	GP2
Funmilola	Taiwo			University College Hospital	Ibadan, Nigeria	Data Contributor	GP2
Francisca	Nwaokorie			University of Lagos	Lagos, Nigeria	Data Contributor	GP2
Lasse	Pihlstrøm			Oslo University Hospital	Oslo, Norway	Data Contributor	GP2
Manuela	Tan			Oslo University Hospital	Oslo, Norway	Data Contributor	GP2
Ingeborg Haugesag	Lie			Oslo University Hospital	Oslo, Norway	Data Contributor	GP2
Jodi	Maple-Grødem			Stavanger University Hospital	Stavanger, Norway	Data Contributor	GP2
Solveig E J	Dalbro			Oslo University Hospital	Oslo, Norway	Data Contributor	GP2
Ellen Hoven	Maurtveten			Oslo University Hospital	Oslo, Norway	Data Contributor	GP2
Shoaib	Ur-Rehman			University of Science and Technology Bannu	Bannu, Pakistan	Data Contributor	GP2
Mohamed	Nour			Razi Hospital	Rawalpindi, Pakistan	Data Contributor	GP2
Mario	Cornejo-Olivas			Universidad Cientifica del Sur	Lima, Peru	Data Contributor	GP2
Nicanor Mori	Quispe			Hospital Nacional Daniel A. Carrión	Lima, Peru	Data Contributor	GP2
JULIA ESTHER RIOS	PINTO			UNIVERSIDAD PERUANA LOS ANDES	Huancayo, Peru	Data Contributor	GP2
Maryenela	Illanes-Manrique			Instituto Nacional de Ciencias Neurologicas	Lima, Peru	Data Contributor	GP2
Cintia Margoth Armas	Puente			Instituto Nacional de Ciencias Neurológicas	Lima, Peru	Data Contributor	GP2
Torres	Luis			Instituto Nacional de Ciencias Neurologicas	Lima, Peru	Data Contributor	GP2
Maria Leila	Doquenia			Metropolitan Medical Center	Manila, Philippines	Data Contributor	GP2
Raymond	Rosales			Metropolitan Medical Center	Manila, Philippines	Data Contributor	GP2
Gerard	Saranza			Chong Hua Hospital	Cebu, Philippines	Data Contributor	GP2

*First name, last name, and suffix (if applicable) are required and will appear in PubMed.

***First Name and Middle Initial(s)**	***Last Name**	***Suffix (eg, Jr, III)**	Academic Degrees	Institution	Location (city, state/province, country)	Role or Contribution, eg, chair, principal investigator	Group (if more than 1 Group listed in the byline) and/or Subgroup (eg, Steering Committee)
Agata	Gajos			Medical University of Lodz	Lodz, Poland	Data Contributor	GP2
Elena	Iakovenko			Research Center of Neurology	Moscow, Russia	Data Contributor	GP2
Anna	Gareeva			Ufa Federal Research Center	Ufa, Russia	Data Contributor	GP2
Gulnara	Akhmadeeva			Ufa Scientific Center	Ufa, Russia	Data Contributor	GP2
Irina	Gilyazova			Russian Academy of Sciences / Bashkir State Medical University	Ufa, Russia	Data Contributor	GP2
Bashayer Al	Mubarak			King Faisal Specialist Hospital and Research Center	Riyadh, Saudi Arabia	Data Contributor	GP2
Muhammad	Umair			King Abdullah International Medical Research Center	Jeddah, Saudi Arabia	Data Contributor	GP2
Nada Abdullah	Altassan			King Faisal Specialist Hospital and Research Center	Riyadh, Saudi Arabia	Data Contributor	GP2
Nataša Dragašević	Mišković			Neurology Clinic, University Clinical Center of Serbia	Belgrade, Serbia	Data Contributor	GP2
Andona	Milovanović			Neurology Clinic, University Clinical Center of Serbia	Belgrade, Serbia	Data Contributor	GP2
Eng-King	Tan			National Neuroscience Institute	Singapore, Singapore	Data Contributor	GP2
Jia Nee	Foo			Nanyang Technological University	Singapore, Singapore	Data Contributor	GP2
Elaine	Chew			Nanyang Technological University	Singapore, Singapore	Data Contributor	GP2
Vesna Van	Midden			Ljubljana University Medical Centre	Ljubljana, Slovenia	Data Contributor	GP2
Ferzana	Amod			University of KwaZulu-Natal	Durban, South Africa	Data Contributor	GP2
Jonathan	Carr			University of Stellenbosch	Stellenbosch, South Africa	Data Contributor	GP2
Soraya	Bardien			Stellenbosch University	Stellenbosch, South Africa	Data Contributor	GP2
Nikita	Pillay			University of the Western Cape	Bellville, South Africa	Data Contributor	GP2

*First name, last name, and suffix (if applicable) are required and will appear in PubMed.

***First Name and Middle Initial(s)**	***Last Name**	***Suffix (eg, Jr, III)**	Academic Degrees	Institution	Location (city, state/province, country)	Role or Contribution, eg, chair, principal investigator	Group (if more than 1 Group listed in the byline) and/or Subgroup (eg, Steering Committee)
Kathryn	Step			Stellenbosch University	Cape Town, South Africa	Data Contributor	GP2
Riaan Van	Coller			University of Pretoria	Pretoria, South Africa	Data Contributor	GP2
Beomseok	Jeon			Seoul National University Hospital	Seoul, South Korea	Data Contributor	GP2
Yun Joong	Kim			Yongin Severance Hospital	Seoul, South Korea	Data Contributor	GP2
Jung Hwan	Shin			Seoul National University	Seoul, South Korea	Data Contributor	GP2
Joowon	Jang			Seoul National University	Seoul, South Korea	Data Contributor	GP2
Jee-Young	Lee			SMG-SNU Boramae Medical Center, College of Medicine Seoul National University	Seoul, South Korea	Data Contributor	GP2
Ryul	Kim			SMG-SNU Boramae Meidcal Center	Seoul, South Korea	Data Contributor	GP2
HAN-Joon	Kim			Seoul National University Hospital	Seoul, South Korea	Data Contributor	GP2
Esther	Cubo			Hospital Universitario Burgos	Burgos, Spain	Data Contributor	GP2
Ignacio	Alvarez			University Hospital Mutua Terrassa	Barcelona, Spain	Data Contributor	GP2
Janet	Hoenicka			Institut de Recerca Sant Joan de Deu	Barcelona, Spain	Data Contributor	GP2
Katrin	Beyer			Research Institute Germans Trias i Pujol	Barcelona, Spain	Data Contributor	GP2
Maria Teresa	Periñan			Instituto de Biomedicina de Sevilla	Seville, Spain	Data Contributor	GP2
Pau	Pastor			University Hospital Germans Trias i Pujol	Barcelona, Spain	Data Contributor	GP2
Ruben	Fernandez-Santiago			Hospital Clínic de Barcelona	Barcelona, Spain	Data Contributor	GP2
Pilar Gómez	Garre			Instituto de Biomedicina de Sevilla	Seville, Spain	Data Contributor	GP2

*First name, last name, and suffix (if applicable) are required and will appear in PubMed.

***First Name and Middle Initial(s)**	***Last Name**	***Suffix (eg, Jr, III)**	Academic Degrees	Institution	Location (city, state/province, country)	Role or Contribution, eg, chair, principal investigator	Group (if more than 1 Group listed in the byline) and/or Subgroup (eg, Steering Committee)
Pablo	Mir			Instituto de Biomedicina de Sevilla	Seville, Spain	Data Contributor	GP2
Mario	Ezquerra			FCRB-IDIBAPS	Barcelona, Spain	Data Contributor	GP2
Celia Painous	Marti			Hospital Clinic Barcelona	Barcelona, Spain	Data Contributor	GP2
Lola J.	Díaz-Feliz			Fernando Pessoa University, San Roque Hospital	Las Palmas de Gran Canaria, Spain	Data Contributor	GP2
José Matías Arbelo	González			Hospital Universitario San Roque Las Palmas/ Universidad Fernando Pessoa Canarias (UFPC)	Las Palmas de Gran Canaria, Spain	Data Contributor	GP2
Juan Carlos Martínez	Castrillo			Hospital Ramón y Cajal	Madrid, Spain	Data Contributor	GP2
Marina	Mata			Hospital Universitario Infanta Sofia	Madrid, Spain	Data Contributor	GP2
Oriol De	Fabregues			Hospital Universitari Vall d'Hebron	Barcelona, Spain	Data Contributor	GP2
Lydia	Vela-Desojo			Hospital Universitario Fundación Alcorcón	Madrid, Spain	Data Contributor	GP2
Manuel Menendez	Gonzalez			Hospital Universitario Central de Asturias	Oviedo, Spain	Data Contributor	GP2
Yaroslau	Compta			IDIBAPS / Hospital Clinic	Barcelona, Spain	Data Contributor	GP2
Alicia	Garrido			IDIBAPS-FCRB. Hospital Clinic Barcelona	Barcelona, Spain	Data Contributor	GP2
Maria J	Marti			Hospital Clinic de Barcelona. Institut d'Investigacio Biomedica August Pi i Sunyer (IDIBAPS)	Barcelona, Spain	Data Contributor	GP2
Almudena	Sánchez-Gómez			Hospital Clinic of Barcelona	Barcelona, Spain	Data Contributor	GP2
Alexia T. Sánchez	Reyes			Universidad Fernando Pessoa Canarias	Las Palmas de Gran Canaria, Spain	Data Contributor	GP2

Supplemental Online Content: Nonauthor Collaborators
*First name, last name, and suffix (if applicable) are required and will appear in PubMed.

***First Name and Middle Initial(s)**	***Last Name**	***Suffix (eg, Jr, III)**	Academic Degrees	Institution	Location (city, state/province, country)	Role or Contribution, eg, chair, principal investigator	Group (if more than 1 Group listed in the byline) and/or Subgroup (eg, Steering Committee)
Laia Muñoz	Llahuna			IR Sant Pau	Barcelona, Spain	Data Contributor	GP2
Joaquim Aumatell	Escabies			IR SANT PAU	Barcelona, Spain	Data Contributor	GP2
Javier Pagonabarraga	Mora			IR SANT PAU	Barcelona, Spain	Data Contributor	GP2
Ignacio Illán	Gala			IR SANT PAU	Barcelona, Spain	Data Contributor	GP2
Esteban	Muñoz			Hospital Clínic de Barcelona	Barcelona, Spain	Data Contributor	GP2
Manuela San Eufrasio	Martínez			Instituto de Biomedicina de Sevilla	Sevilla, Spain	Data Contributor	GP2
Laura Muñoz	Delgado			Instituto de Biomedicina de Sevilla	Sevilla, Spain	Data Contributor	GP2
Rafael Díaz	Belloso			Instituto de Biomedicina de Sevilla	Sevilla, Spain	Data Contributor	GP2
Sergio García	Díaz			Instituto de Biomedicina de Sevilla	Sevilla, Spain	Data Contributor	GP2
Marta Bonilla	Toribio			Instituto de Biomedicina de Sevilla	Sevilla, Spain	Data Contributor	GP2
Dolores Buiza	Rueda			Instituto de Biomedicina de Sevilla	Sevilla, Spain	Data Contributor	GP2
Antonio Cristobal Luque	Ambrosiani			Instituto de Biomedicina de Sevilla	Sevilla, Spain	Data Contributor	GP2
Silvia Jesus	Maestre			Instituto de Biomedicina de Sevilla	Sevilla, Spain	Data Contributor	GP2
Daniel Macías	García			Instituto de Biomedicina de Sevilla	Sevilla, Spain	Data Contributor	GP2
Elena Ojeda	Lepe			Instituto de Biomedicina de Sevilla	Sevilla, Spain	Data Contributor	GP2
Rocío Pineda	Sánchez			Instituto de Biomedicina de Sevilla	Sevilla, Spain	Data Contributor	GP2
Ana Castellano	Guerrero			Instituto de Biomedicina de Sevilla	Sevilla, Spain	Data Contributor	GP2

Supplemental Online Content: Nonauthor Collaborators
*First name, last name, and suffix (if applicable) are required and will appear in PubMed.

***First Name and Middle Initial(s)**	***Last Name**	***Suffix (eg, Jr, III)**	Academic Degrees	Institution	Location (city, state/province, country)	Role or Contribution, eg, chair, principal investigator	Group (if more than 1 Group listed in the byline) and/or Subgroup (eg, Steering Committee)
Astrid Daniela Adarmes	Gómez			Instituto de Biomedicina de Sevilla	Sevilla, Spain	Data Contributor	GP2
Cristina Pérez	Calvo			Instituto de Biomedicina de Sevilla	Seville, Spain	Data Contributor	GP2
Alejandro Salguero	Oviedo			Instituto de Biomedicina de Sevilla	Sevilla, Spain	Data Contributor	GP2
Lorena Clavijo	Jiménez			Instituto de Biomedicina de Sevilla	Sevilla, Spain	Data Contributor	GP2
Natalia	Lopez			Global Parkinson's Genetics Program (GP2)	Madrid, Spain	Data Contributor	GP2
Gamini Karapitiya	Pathirana			National Hospital of Sri Lanka	Colombo, Sri Lanka	Data Contributor	GP2
Ruwani	Wijeyekoon			Association of Sri Lankan Neurologists	Colombo, Sri Lanka	Data Contributor	GP2
Muhammed Saamir	Mohideen			National Hospital Galle	Galle, Sri Lanka	Data Contributor	GP2
Manjula	Caldera			Teaching Hospital Anuradhapura	Anuradhapura, Sri Lanka	Data Contributor	GP2
Arjuna	Fernando			National Hospital of Sri Lanka	Colombo, Sri Lanka	Data Contributor	GP2
Darshana	Sirisena			CNTH Ragama	Ragama, Sri Lanka	Data Contributor	GP2
Senaka	Bandusena			National Hospital of Sri Lanka	Colombo, Sri Lanka	Data Contributor	GP2
Thashi	Chang			University of Colombo, Sri Lanka	Colombo, Sri Lanka	Data Contributor	GP2
Darshana Dias	Wijegunasinghe			Colombo North Teaching hospital	Colombo, Sri Lanka	Data Contributor	GP2
Gamini	Pathirana			NHSL	Colombo, Sri Lanka	Data Contributor	GP2
Bimsara Sajotha Nalin	Senanayake			Institution of Neurology, National Hospital of Sri Lanka	Colombo, Sri Lanka	Data Contributor	GP2
Ajantha	Keshavaraj			Teaching Hospital Jaffna	Jaffna, Sri Lanka	Data Contributor	GP2
Sarah	El-Sadig			Faculty of medicine university of Khartoum	Khartoum, Sudan	Data Contributor	GP2

Supplemental Online Content: Nonauthor Collaborators
*First name, last name, and suffix (if applicable) are required and will appear in PubMed.

***First Name and Middle Initial(s)**	***Last Name**	***Suffix (eg, Jr, III)**	Academic Degrees	Institution	Location (city, state/province, country)	Role or Contribution, eg, chair, principal investigator	Group (if more than 1 Group listed in the byline) and/or Subgroup (eg, Steering Committee)
Inas	Elsayed			Faculty of pharmacy University of Gezira	Wad Madani, Sudan	Data Contributor	GP2
Kajsa	Brolin			Lund University	Lund, Sweden	Data Contributor	GP2
Per	Svenningsson			Karolinska Institute	Stockholm, Sweden	Data Contributor	GP2
Maria	Swanberg			Lund University	Lund, Sweden	Data Contributor	GP2
Christiane	Zweier			Inselspital Bern, University of Bern	Bern, Switzerland	Data Contributor	GP2
Gerd	Tinkhauser			University Hospital Bern	Bern, Switzerland	Data Contributor	GP2
Paul	Krack			Inselspital Bern, University of Bern	Bern, Switzerland	Data Contributor	GP2
Deborah	Bartholdi			University Hospital Bern	Bern, Switzerland	Data Contributor	GP2
Jonas	Ihle			Hopital Triemli Zürich	Zürich, Switzerland	Data Contributor	GP2
Chin-Hsien	Lin			National Taiwan University Hospital	Taipei City, Taiwan	Data Contributor	GP2
Hsiu-Chuan	Wu			Chang Gung Memorial Hospital	Taoyuan City, Taiwan	Data Contributor	GP2
Pin-Jui	Kung			National Taiwan University	Taipei City, Taiwan	Data Contributor	GP2
Ruey-Meei	Wu			National Taiwan University Hospital	Taipei City, Taiwan	Data Contributor	GP2
Yihru	Wu			Chang Gung Memorial Hospital	Taoyuan City, Taiwan	Data Contributor	GP2
Pin-Shiuan,	Chen			National Taiwan University Hospital	Taipei, Taiwan	Data Contributor	GP2
Ganieva	Manizha			Avicenna Tajik State Medical University	Dushanbe, Tajikistan	Data Contributor	GP2
Maksudjon	Isrofilov			Avicenna Tajik State Medical University	Dushanbe, Tajikistan	Data Contributor	GP2
Kigocha	Okeng'o			Muhimbili National Hospital-Mloganzila	Dar es Salaam, Tanzania	Data Contributor	GP2
Philip	Adebayo			The Aga Khan University	Dar es Salaam, Tanzania	Data Contributor	GP2
Rim	Amouri			Mongi Ben Hmida National Institute of Neurolog	Tunis, Tunisia	Data Contributor	GP2

Supplemental Online Content: Nonauthor Collaborators
*First name, last name, and suffix (if applicable) are required and will appear in PubMed.

***First Name and Middle Initial(s)**	***Last Name**	***Suffix (eg, Jr, III)**	Academic Degrees	Institution	Location (city, state/province, country)	Role or Contribution, eg, chair, principal investigator	Group (if more than 1 Group listed in the byline) and/or Subgroup (eg, Steering Committee)
Samia Ben	Sassi			Mongi Ben Hmida National Institute of Neurology	Tunis, Tunisia	Data Contributor	GP2
Chokri	Mhiri			Habib Bourguiba University Hospital	Sfax, Tunisia	Data Contributor	GP2
Nabli Fatnassi	Fatma			National institute Mongi Ben Hmida of Neurology	Tunis, Tunisia	Data Contributor	GP2
Amine	Rachdi			Mongi Ben Hamida institute of Neurology	NA, Tunisia	Data Contributor	GP2
Zakaria	Saied			National Institute Mongi Ben Hamida of Neurology	Tunis, Tunisia	Data Contributor	GP2
Mouna Ben	Djebara			Razi Hospital	Tunis, Tunisia	Data Contributor	GP2
Rania	Zouari			National institute of neurology mongi ben hmida	Tunis, Tunisia	Data Contributor	GP2
A. Nazlı	Başak			Koç University	Istanbul, Turkey	Data Contributor	GP2
Özgür Öztop	Çakmak			Koç University	Istanbul, Turkey	Data Contributor	GP2
Sibel	Ertan			Koç University	Istanbul, Turkey	Data Contributor	GP2
Rezzak	Yilmaz			University of Ankara	Ankara, Turkey	Data Contributor	GP2
Binnur	Çelik			University of Ankara	Ankara, Turkey	Data Contributor	GP2
Gençer	Genç			Şişli Etfal Training and Research Hospital, University of Health Sciences, İstanbul, TR	Istanbul, Turkey	Data Contributor	GP2
Muhittin Cenk	Akbostancı			Private Practice	Ankara, Turkey	Data Contributor	GP2
Basar	Bilgic			Istanbul University, Faculty of Medicine	Istanbul, Turkey	Data Contributor	GP2
Bedia	Samanci			Istanbul University, Faculty of Medicine	Istanbul, Turkey	Data Contributor	GP2
Murat	Emre			Istanbul university	Istanbul, Turkey	Data Contributor	GP2
Haşmet	Hanağasi			ISTANBUL FACULTY OF MEDICINE	Istanbul, Turkey	Data Contributor	GP2
Aysegul	Gunduz			Istanbul University-Cerrahpasa, Cerrahpasa Faculty of Medicine	Istanbul, Turkey	Data Contributor	GP2

Supplemental Online Content: Nonauthor Collaborators
*First name, last name, and suffix (if applicable) are required and will appear in PubMed.

*First Name and Middle Initial(s)	*Last Name	*Suffix (eg, Jr, III)	Academic Degrees	Institution	Location (city, state/province, country)	Role or Contribution, eg, chair, principal investigator	Group (if more than 1 Group listed in the byline) and/or Subgroup (eg, Steering Committee)
Gulcin Benbir	Senel			Istanbul University-Cerrahpasa, Cerrahpasa Faculty of Medicine	Istanbul, Turkey	Data Contributor	GP2
Alastair	Noyce			Queen Mary University of London	London, United Kingdom	Data Contributor	GP2
Anette	Schrag			University College London	London, United Kingdom	Data Contributor	GP2
Anthony	Schapira			University College London	London, United Kingdom	Data Contributor	GP2
Camille	Carroll			University of Plymouth	Plymouth, United Kingdom	Data Contributor	GP2
Donald	Grosset			University of Glasgow	Glasgow, United Kingdom	Data Contributor	GP2
Eleanor J.	Stafford			University College London	London, United Kingdom	Data Contributor	GP2
Henry	Houlden			University College London	London, United Kingdom	Data Contributor	GP2
Huw R	Morris			University College London	London, United Kingdom	Data Contributor	GP2
John	Hardy			University College London	London, United Kingdom	Data Contributor	GP2
Kin Ying	Mok			Univeristy College London	London, United Kingdom	Data Contributor	GP2
Mie	Rizig			University College London	London, United Kingdom	Data Contributor	GP2
Nicholas	Wood			University College London	London, United Kingdom	Data Contributor	GP2
Nigel	Williams			Cardiff University	Cardiff, United Kingdom	Data Contributor	GP2
Olaitan	Okunoye			University College London	London, United Kingdom	Data Contributor	GP2

Supplemental Online Content: Nonauthor Collaborators
*First name, last name, and suffix (if applicable) are required and will appear in PubMed.

*First Name and Middle Initial(s)	*Last Name	*Suffix (eg, Jr, III)	Academic Degrees	Institution	Location (city, state/province, country)	Role or Contribution, eg, chair, principal investigator	Group (if more than 1 Group listed in the byline) and/or Subgroup (eg, Steering Committee)
Rauan	Kaiyrzhanov			University College London	London, United Kingdom	Data Contributor	GP2
Rimona	Weil			University College London	London, United Kingdom	Data Contributor	GP2
Seth	Love			University of Bristol	Bristol, United Kingdom	Data Contributor	GP2
Simona	Jasaityte			University College London	London, United Kingdom	Data Contributor	GP2
Sumit	Dey			Queen Mary University of London	London, United Kingdom	Data Contributor	GP2
Spencer	Finch			Queen Mary University of London	London, United Kingdom	Data Contributor	GP2
Valentina	Escott-Price			Cardiff University	Cardiff, United Kingdom	Data Contributor	GP2
Hamin	Lee			St George's, University of London	London, United Kingdom	Data Contributor	GP2
Roger	Barker			University of Cambridge	Cambridge, United Kingdom	Data Contributor	GP2
Mina	Ryten			University College London	London, United Kingdom	Data Contributor	GP2
Michele	Hu			University of Oxford	Oxford, United Kingdom	Data Contributor	GP2
Laura	Parkkinen			University of Oxford	Oxford, United Kingdom	Data Contributor	GP2
Kailash	Bhatia			University College London	London, United Kingdom	Data Contributor	GP2
Richard	Walker			Northumbria Healthcare at NHS Foundation Trust	Newcastle upon Tyne, United Kingdom	Data Contributor	GP2
Steve	Gentleman			Imperial College London	London, United Kingdom	Data Contributor	GP2

Supplemental Online Content: Nonauthor Collaborators
*First name, last name, and suffix (if applicable) are required and will appear in PubMed.

***First Name and Middle Initial(s)**	***Last Name**	***Suffix (eg, Jr, III)**	Academic Degrees	Institution	Location (city, state/province, country)	Role or Contribution, eg, chair, principal investigator	Group (if more than 1 Group listed in the byline) and/or Subgroup (eg, Steering Committee)
Thomas	Warner			University College London	London, United Kingdom	Data Contributor	GP2
David	Burn			Newcastle University	Newcastle upon Tyne, United Kingdom	Data Contributor	GP2
Christian	Lambert			Imperial College London	London, United Kingdom	Data Contributor	GP2
Caroline	Williams-Gray			University of Cambridge	Cambridge, United Kingdom	Data Contributor	GP2
Deborah	Attuah			YLD	London, United Kingdom	Data Contributor	GP2
Raquel	Real			University College London	London, United Kingdom	Data Contributor	GP2
Yen	Tai			Imperial College London	London, United Kingdom	Data Contributor	GP2
Alexandra	Zirra			Queen Mary University of London	London, United Kingdom	Data Contributor	GP2
Christopher M	Morris			Newcastle University	Newcastle upon Tyne, United Kingdom	Data Contributor	GP2
Matilda Lily	Fenn			University College London	London, United Kingdom	Data Contributor	GP2
Andrew C	Robinson			The University of Manchester	Manchester, United Kingdom	Data Contributor	GP2
Lesley Yue	Wu			University College London	London, United Kingdom	Data Contributor	GP2
Tessa Du	Toit			UCL	Londo, United Kingdom	Data Contributor	GP2
Joshua Luc Isherwood	Frost			UCL Queen Square Institute of Neurology	London, United Kingdom	Data Contributor	GP2
Federico	Roncaroli			University of Manchester	Manchester, United Kingdom	Data Contributor	GP2

Supplemental Online Content: Nonauthor Collaborators
*First name, last name, and suffix (if applicable) are required and will appear in PubMed.

***First Name and Middle Initial(s)**	***Last Name**	***Suffix (eg, Jr, III)**	Academic Degrees	Institution	Location (city, state/province, country)	Role or Contribution, eg, chair, principal investigator	Group (if more than 1 Group listed in the byline) and/or Subgroup (eg, Steering Committee)
Ashvin	Kuri			Queen Mary University of London	London, United Kingdom	Data Contributor	GP2
Sheena	Waters			Queen Mary University of London	London, United Kingdom	Data Contributor	GP2
Laura	Smith			Queen Mary University of London	London, United Kingdom	Data Contributor	GP2
Eduardo De	Pablo-Fernández			Queen Mary University of London	London, United Kingdom	Data Contributor	GP2
Anisa	Shahid			Queen Mary University of London	London, United Kingdom	Data Contributor	GP2
Cristina	Simonet			Queen Mary University of London	London, United Kingdom	Data Contributor	GP2
Charlotte	Dore			University College London	London, United Kingdom	Data Contributor	GP2
Oiher	Serrano-Asensio			University College London	London, United Kingdom	Data Contributor	GP2
Marco	Toffoli			University College London	London, United Kingdom	Data Contributor	GP2
Riona	Fumi			University College London, Institute of Neurology	London, United Kingdom	Data Contributor	GP2
Brook	Huxford			Queen Mary University of London	London, United Kingdom	Data Contributor	GP2
Harneek	Chohan			Queen Mary University of London	London, United Kingdom	Data Contributor	GP2
Sophie	Meyer			Queen Mary University of London	London, United Kingdom	Data Contributor	GP2
Laura	Pérez-Carbonell			Queen Mary University London / Guy's and St Thomas' NHS Foundation Trust	London, United Kingdom	Data Contributor	GP2
Solomiia	Bandrivska			University College London	London, United Kingdom	Data Contributor	GP2

*First name, last name, and suffix (if applicable) are required and will appear in PubMed.

***First Name and Middle Initial(s)**	***Last Name**	***Suffix (eg, Jr, III)**	Academic Degrees	Institution	Location (city, state/province, country)	Role or Contribution, eg, chair, principal investigator	Group (if more than 1 Group listed in the byline) and/or Subgroup (eg, Steering Committee)
Saiesha	Dindayal			University College London	London, United Kingdom	Data Contributor	GP2
Charlotte	Andrews			Queen Mary University of London	London, United Kingdom	Data Contributor	GP2
Emily Navarro	Jones			University College London	London, United Kingdom	Data Contributor	GP2
Kamalesh	Dey			Queen Mary University of London	London, United Kingdom	Data Contributor	GP2
Faiza	Durrani			Centre for Preventive Neurology	London, United Kingdom	Data Contributor	GP2
Marte	Jensen			University College London	London, United Kingdom	Data Contributor	GP2
Yoana	Kordovska			University College London	London, United Kingdom	Data Contributor	GP2
Jennifer	Brady			University College London	London, United Kingdom	Data Contributor	GP2
Nolia	Lumley			University College London	London, United Kingdom	Data Contributor	GP2
Erika	Lam			University College London	London, United Kingdom	Data Contributor	GP2
Tatiana	Georgiades			University College London	London, United Kingdom	Data Contributor	GP2
Kawmadi Wasundera	Gunawardena			Oxford University Hospitals NHS Foundation Trust	Oxford, United Kingdom	Data Contributor	GP2
Víctor E. Raggio	Risso			Facultad de Medciina, Universidad de la República	Montevideo, Uruguay	Data Contributor	GP2
Elena	M.Dieguez			Facultad de Medicina universidad de de la tepublica montevideo uruguay	Montevideo, Uruguay	Data Contributor	GP2

Supplemental Online Content: Nonauthor Collaborators
*First name, last name, and suffix (if applicable) are required and will appear in PubMed.

*First Name and Middle Initial(s)	*Last Name	*Suffix (eg, Jr, III)	Academic Degrees	Institution	Location (city, state/province, country)	Role or Contribution, eg, chair, principal investigator	Group (if more than 1 Group listed in the byline) and/or Subgroup (eg, Steering Committee)
Lara M.	Lange			Laboratory of Neurogenetics, National Institute on Aging/Institute of Neurogenetics, University of Luebeck	Bethesda, USA	Data Contributor	GP2
Angel	Vinuela			University of Puerto Rico	San Juan, USA	Data Contributor	GP2
Alyssa	O'Grady			The Michael J. Fox Foundation for Parkinson's Research	New York, USA	Data Contributor	GP2
Andrew B	Singleton			Global Parkinson’s Genetics Program (GP2)	Bethesda, USA	Data Contributor	GP2
Andrew K.	Sobering			Augusta University / University of Georgia Medical Partnership	Augusta, USA	Data Contributor	GP2
Bernadette	Siddiqi			The Michael J. Fox Foundation for Parkinson's Research	New York, USA	Data Contributor	GP2
Bradford	Casey			The Michael J. Fox Foundation for Parkinson's Research	New York, USA	Data Contributor	GP2
Brian	Fiske			The Michael J. Fox Foundation for Parkinson's Research	New York, USA	Data Contributor	GP2
Cabell	Jonas			Mid-Atlantic Permanente Medical Group	Bethesda, USA	Data Contributor	GP2
Carlos	Cruchaga			Washington University	St. Louis, USA	Data Contributor	GP2
Caroline B.	Pantazis			Coalition for Aligning Science	Bethesda, USA	Data Contributor	GP2
Claire	Wegel			Indiana University	Bloomington, USA	Data Contributor	GP2
Cornelis	Blauwendraat			Aligning Science Across Parkinson's (ASAP)	Bethesda, USA	Data Contributor	GP2
Dan	Vitale			Data Tecnica	Bethesda, USA	Data Contributor	GP2
Deborah	Hall			Rush University	Chicago, USA	Data Contributor	GP2
Dena	Hernandez			National Institutes of Health	Bethesda, USA	Data Contributor	GP2
Ekemini	Riley			Coalition for Aligning Science	Washington, USA	Data Contributor	GP2
Faraz	Faghri			Data Tecnica	Bethesda, USA	Data Contributor	GP2

Supplemental Online Content: Nonauthor Collaborators
*First name, last name, and suffix (if applicable) are required and will appear in PubMed.

***First Name and Middle Initial(s)**	***Last Name**	***Suffix (eg, Jr, III)**	Academic Degrees	Institution	Location (city, state/province, country)	Role or Contribution, eg, chair, principal investigator	Group (if more than 1 Group listed in the byline) and/or Subgroup (eg, Steering Committee)
Geidy E.	Serrano			Banner Sun Health Research Institute	Sun City, USA	Data Contributor	GP2
Hampton	Leonard			Data Tecnica	Bethesda, USA	Data Contributor	GP2
Hirotaka	Iwaki			Data Tecnica	Washington, USA	Data Contributor	GP2
Honglei	Chen			Michigan State University	East Lansing, USA	Data Contributor	GP2
Ignacio F.	Mata			Cleveland Clinic	Cleveland, USA	Data Contributor	GP2
Ignacio Juan Keller	Sarmiento			Northwestern University	Evanston, USA	Data Contributor	GP2
Jared	Williamson			Kaiser Permanente	Oakland, USA	Data Contributor	GP2
Jonggeol Jeff	Kim			Baylor College of Medicine	Bethesda, USA	Data Contributor	GP2
Joseph	Jankovic			Baylor College of Medicine	Houston, USA	Data Contributor	GP2
Joshua	Shulman			Baylor College of Medicine / Texas Children's Hospital	Houston, USA	Data Contributor	GP2
J	Solle			The Michael J. Fox Foundation for Parkinson's Research	New York, USA	Data Contributor	GP2
Kaileigh	Murphy			The Michael J. Fox Foundation for Parkinson's Research	New York, USA	Data Contributor	GP2
Kamalini Ghosh	Galvelis			Parkinson's Foundation	Princeton, USA	Data Contributor	GP2
Karen	Nuytemans			University of Miami Miller School of Medicine	Miami, USA	Data Contributor	GP2
Karl	Kieburtz			Beth Israel Deaconess Medical Center	Boston, USA	Data Contributor	GP2
Katerina	Markopoulou			North Shore University Health System	Chicago, USA	Data Contributor	GP2
Kenneth	Marek			Institute for Neurodegenerative Disorders	New Haven, USA	Data Contributor	GP2
Kristin S.	Levine			Data Tecnica	Washington, USA	Data Contributor	GP2
Lana M.	Chahine			University of Pittsburgh	Pittsburgh, USA	Data Contributor	GP2
Laura	Ibanez			Washington University	St. Louis, USA	Data Contributor	GP2
Laurel	Screven			Global Parkinson’s Genetics Program (GP2)	Bethesda, USA	Data Contributor	GP2

Supplemental Online Content: Nonauthor Collaborators
*First name, last name, and suffix (if applicable) are required and will appear in PubMed.

*First Name and Middle Initial(s)	*Last Name	*Suffix (eg, Jr, III)	Academic Degrees	Institution	Location (city, state/province, country)	Role or Contribution, eg, chair, principal investigator	Group (if more than 1 Group listed in the byline) and/or Subgroup (eg, Steering Committee)
Lauren	Ruffrage			University of Alabama at Birmingham	Birmingham, USA	Data Contributor	GP2
Lisa	Shulman			University of Maryland	Baltimore, USA	Data Contributor	GP2
Luca	Marsili			University of Cincinnati	Cincinnati, USA	Data Contributor	GP2
Maggie	Kuhl			The Michael J. Fox Foundation for Parkinson's Research	New York, USA	Data Contributor	GP2
Marissa	Dean			University of Alabama at Birmingham	Birmingham, USA	Data Contributor	GP2
Mary B	Makarious			Data Tecnica	Bethesda, USA	Data Contributor	GP2
Mathew	Koretsky			Data Tecnica	Bethesda, USA	Data Contributor	GP2
Megan J.	Puckelwartz			Northwestern University	Chicago, USA	Data Contributor	GP2
Mike A.	Nalls			Data Tecnica	Bethesda, USA	Data Contributor	GP2
Naomi	Louie			The Michael J. Fox Foundation for Parkinson's Research	New York, USA	Data Contributor	GP2
Niccolò Emanuele	Mencacci			Northwestern University	Evanston, USA	Data Contributor	GP2
Roger	Albin			Universit of Michigan	Ann Arbor, USA	Data Contributor	GP2
Roy	Alcalay			Columbia University	New York, USA	Data Contributor	GP2
Ruth	Walker			James J. Peters Veterans Affairs Medical Center	New York, USA	Data Contributor	GP2
Sohini	Chowdhury			The Michael J. Fox Foundation for Parkinson's Research	New York, USA	Data Contributor	GP2
Sonya	Dumanis			Aligning Science Across Parkinson's	Washington, USA	Data Contributor	GP2
Steven	Lubbe			Northwestern University	Chicago, USA	Data Contributor	GP2
Tao	Xie			University of Chicago	Chicago, USA	Data Contributor	GP2
Tatiana	Foroud			Indiana University School of Medicine	Indianapolis, USA	Data Contributor	GP2
Thomas	Beach			Sun Health Research Institution	Sun City, USA	Data Contributor	GP2
Todd	Sherer			The Michael J Fox Foundation for Parkinson's Research	New York, USA	Data Contributor	GP2

Supplemental Online Content: Nonauthor Collaborators
*First name, last name, and suffix (if applicable) are required and will appear in PubMed.

***First Name and Middle Initial(s)**	***Last Name**	***Suffix (eg, Jr, III)**	Academic Degrees	Institution	Location (city, state/province, country)	Role or Contribution, eg, chair, principal investigator	Group (if more than 1 Group listed in the byline) and/or Subgroup (eg, Steering Committee)
Dana	Lewis			Aligning Science Across Parkinson's	Baltimore, USA	Data Contributor	GP2
Shreya	Menon			Gladstone Institutes	San Francisco, USA	Data Contributor	GP2
Melissa	Nirenberg			Icahn School of Medicine at Mount Sinai	New York, USA	Data Contributor	GP2
Spencer	Grant			National Institutes of Health	Bethesda, USA	Data Contributor	GP2
Shannon	Ballard			Data Tecnica	Bethesda, USA	Data Contributor	GP2
Chad	Shaw			Baylor College of Medicine	Houston, USA	Data Contributor	GP2
Sidra	Aslam			Banner Health	Phoenix, USA	Data Contributor	GP2
Devin	Sharp			Aligning Science Across Parkinson's	Vancouver, USA	Data Contributor	GP2
Rachel	Saunders-Pullman			Icahn School of Medicine at Mount Sinai	New York, USA	Data Contributor	GP2
Michiko Kimura	Bruno			The Queen's Medical Center	Honolulu, USA	Data Contributor	GP2
Matt	Farrer			University of Florida College of Medicine	Gainesville, USA	Data Contributor	GP2
Haydeh	Payami			The University of Alabama at Birmingham Heersink School of Medicine	Birmingham, USA	Data Contributor	GP2
Ryan	Pfingst			The Michael J Fox Foundation	New York, USA	Data Contributor	GP2
James B	Leverenz			Cleveland Clinic	Cleveland, USA	Data Contributor	GP2
Elizabeth	Disbrow			LSU Health Shreveport	Shreveport, USA	Data Contributor	GP2
Debi	Brooks			The Michael J Fox Foundation	New York, USA	Data Contributor	GP2
Randy	Schekman			University of California, Berkeley	Berkeley, USA	Data Contributor	GP2
Un	Kang			NYU Grossman School of Medicine	New York, USA	Data Contributor	GP2
Zbigniew K.	Wszolek			Mayo Clinic College of Medicine	Rochester, USA	Data Contributor	GP2
Cyrus	Zabetian			VA Puget Sound Health Care System	Seattle, USA	Data Contributor	GP2

Supplemental Online Content: Nonauthor Collaborators
*First name, last name, and suffix (if applicable) are required and will appear in PubMed.

***First Name and Middle Initial(s)**	***Last Name**	***Suffix (eg, Jr, III)**	Academic Degrees	Institution	Location (city, state/province, country)	Role or Contribution, eg, chair, principal investigator	Group (if more than 1 Group listed in the byline) and/or Subgroup (eg, Steering Committee)
Zach	Chaney			The Michael J Fox Foundation	New York, USA	Data Contributor	GP2
Christine	Swanson-Fischer			National Institutes of Health	Rockville, USA	Data Contributor	GP2
Conor	Hennessey			The Michael J Fox Foundation	New York, USA	Data Contributor	GP2
Cassandra	Barrett			The Michael J Fox Foundation	New York, USA	Data Contributor	GP2
Beate	Ritz			University of California, Los Angeles	Los Angeles, USA	Data Contributor	GP2
Bradley	Boeve			Mayo Clinic	Rochester, USA	Data Contributor	GP2
Ashley	Rawls			University of Florida College of Medicine	Gainesville, USA	Data Contributor	GP2
Holly A.	Shill			Barrow Neurological Institute	Phoenix, USA	Data Contributor	GP2
Erika	Driver-Dunckley			Mayo Clinic AZ	Scottsdale, USA	Data Contributor	GP2
Bruce A.	Chase			Endeavor Health (formerly NorthShore University Health System)	Skokie, USA	Data Contributor	GP2
Mahesh	Padmanaban			University of Chicago	Chicago, USA	Data Contributor	GP2
Thiago Peixoto	Leal			Cleveland Clinic	Cleveland, USA	Data Contributor	GP2
Owen A.	Ross			Mayo Clinic	Jacksonville, USA	Data Contributor	GP2
Michael	Rose			The Ohio State University Medical Center	Columbus, USA	Data Contributor	GP2
Ariane	Park			The Ohio State University Medical Center	Columbus, USA	Data Contributor	GP2
Victoria	Klee			The Ohio State University	Columbus, USA	Data Contributor	GP2
James C.	Beck			Parkinson's Foundation	New York, USA	Data Contributor	GP2
Suzanne	Judd			UAB	Birmingham, USA	Data Contributor	GP2
Daniel	Weintraub			U. Pennsylvania	Philadelphia, USA	Data Contributor	GP2
Vikas	Kotagal			University of Michigan	Ann Arbor, USA	Data Contributor	GP2
Nicolaas I.	Bohnen			University of Michigan	Ann Arbor, USA	Data Contributor	GP2
Prabesh	Kanel			University of Michigan	Ann Arbor, USA	Data Contributor	GP2
Chatkaew	Pongmala			University of Michigan	Ann Arbor, USA	Data Contributor	GP2

Supplemental Online Content: Nonauthor Collaborators
*First name, last name, and suffix (if applicable) are required and will appear in PubMed.

***First Name and Middle Initial(s)**	***Last Name**	***Suffix (eg, Jr, III)**	Academic Degrees	Institution	Location (city, state/province, country)	Role or Contribution, eg, chair, principal investigator	Group (if more than 1 Group listed in the byline) and/or Subgroup (eg, Steering Committee)
Erin	Williams			Van Andel Institute	Grand Rapids, USA	Data Contributor	GP2
Audrey	Strongosky			Mayo Clinic Florida	Jacksonville, USA	Data Contributor	GP2
Michael	Henderson			Van Andel Institute	Grand Rapids, USA	Data Contributor	GP2
Daniel C.	Rohrer			Van Andel Institute	Grand Rapids, USA	Data Contributor	GP2
Alexander	Blanski			Van Andel Research Institute	Grand Rapids, USA	Data Contributor	GP2
Christina	Missler			Van Andel Institute	Grand Rapids, USA	Data Contributor	GP2
Alyssa	Johansson			Van Andel Institute	Grand Rapids, USA	Data Contributor	GP2
Felipe	Duarte-Zambrano			Cleveland Clinic	Cleveland, USA	Data Contributor	GP2
Gist	Croft			The New York Stem Cell Foundation	New York, USA	Data Contributor	GP2
Lisa	Voltolina			New York Stem Cell Foundation Reseach Institute	New York, USA	Data Contributor	GP2
Whitley	Aamodt			University of Pennsylvania	Philadelphia, USA	Data Contributor	GP2
Stewart A	Factor			Emory University	Atlanta, USA	Data Contributor	GP2
Alberto J.	Espay			University of Cincinnati	Cincinnati, USA	Data Contributor	GP2
Nabila	Dahodwala			University of Pennsyvlania	Philadelphia, USA	Data Contributor	GP2
Chantale	Branson			Morehouse School of Medicine	Atlanta, USA	Data Contributor	GP2
Emily	Hill			University of Cincinnati	Cincinnati, USA	Data Contributor	GP2
Krutika	Patel			New York Stem Cell Foundation	Denver, USA	Data Contributor	GP2
Shyamal	Mehta			Mayo Clinic, Arizona	Scottsdale, USA	Data Contributor	GP2
Emily	Waldo			Cleveland Clinic	Cleveland, USA	Data Contributor	GP2
Miguel Inca	Martinez			Cleveland Clinic Foundation	Cleveland, USA	Data Contributor	GP2
Anne-Marie	Wills			Massachusetts General Hospital	Boston, USA	Data Contributor	GP2
Ejaz A.	Shamim			Kaiser Permanente, MidAtlantic Permanente Research Institute	Washington, USA	Data Contributor	GP2
Charles H.	Adler			Mayo Clinic College of Medicine, Mayo Clinic Arizona	Scottsdale, USA	Data Contributor	GP2
Ileana	Lorenzini			Banner Sun Health Research Institute	Sun City, USA	Data Contributor	GP2

Supplemental Online Content: Nonauthor Collaborators
*First name, last name, and suffix (if applicable) are required and will appear in PubMed.

***First Name and Middle Initial(s)**	***Last Name**	***Suffix (eg, Jr, III)**	Academic Degrees	Institution	Location (city, state/province, country)	Role or Contribution, eg, chair, principal investigator	Group (if more than 1 Group listed in the byline) and/or Subgroup (eg, Steering Committee)
Peter	Heutink			Global Parkinson's Genetics Program (GP2)	Pacifica, USA	Data Contributor	GP2
Bernabé	Bustos			Northwestern University Feinberg School of Medicine	Chicago, USA	Data Contributor	GP2
Shalini	Padmanabhan			Michael J. Fox Foundation	New York City, USA	Data Contributor	GP2
Serena	Fong			Michael J. Fox Foundation	New York City, USA	Data Contributor	GP2
Vidhu	Agarwal			National Institute of Health	Bethesda, USA	Data Contributor	GP2
Nicole	Kuznetsov			National Institute of Health	Bethesda, USA	Data Contributor	GP2
Lietsel	Jones			Data Tecnica International	Bethesda, USA	Data Contributor	GP2
Julia	Staisch			Ochsner Clinic Foundation	New Orleans, USA	Data Contributor	GP2
Camilla	Kilbane			University Hospitals Cleveland Medical Center	Cleveland, USA	Data Contributor	GP2
Erin Furr	Stimming			The University of Texas Health Science Center at Houston	Houston, USA	Data Contributor	GP2
Scott	Norris			Washington University in St. Louis	St. Louis, USA	Data Contributor	GP2
Erica	Bell			The Ohio State University Medical Center	Columbus, USA	Data Contributor	GP2
Natalia	Chunga			Louisiana State University Health Sciences Center at Shreveport	Shreveport, USA	Data Contributor	GP2
Mohamed	Elkasaby			University Hospitals Cleveland Medical Center/Case Western Reserve University	Cleveland, USA	Data Contributor	GP2
Shivika	Chandra			UTHealth-Houston	Houston, USA	Data Contributor	GP2
Erin	Foster			Washington University School of Medicine	St. Louis, USA	Data Contributor	GP2
Vanessa	Hinson			MUSC	Charleston, USA	Data Contributor	GP2
Scott A.	Norris			Washington University	St. Louis, USA	Data Contributor	GP2
Andrew Nader	AMeri			MUSC	Charleston, USA	Data Contributor	GP2

Supplemental Online Content: Nonauthor Collaborators
*First name, last name, and suffix (if applicable) are required and will appear in PubMed.

***First Name and Middle Initial(s)**	***Last Name**	***Suffix (eg, Jr, III)**	Academic Degrees	Institution	Location (city, state/province, country)	Role or Contribution, eg, chair, principal investigator	Group (if more than 1 Group listed in the byline) and/or Subgroup (eg, Steering Committee)
Duan	Nguyen			Hue University	Huế, Vietnam	Data Contributor	GP2
Toan	Nguyen			Hue University	Huế, Vietnam	Data Contributor	GP2
NguyễN Thái Thuỳ	Ngân			University Medical Center, Ho Chi Minh City	Ho Chi Minh, Vietnam	Data Contributor	GP2
Ha Ngoc Le	Uyen			University Medical Center Ho Chi Minh city	Ho Chi Minh, Vietnam	Data Contributor	GP2
Tai Ngoc	Tran			University Medical Center HCMC	Ho Chi Minh, Vietnam	Data Contributor	GP2
Khang	Vo			University Medical Center	Ho Chi Minh, Vietnam	Data Contributor	GP2
Vinh Thanh	Nguyen			University Medical Center Ho Chi Minh City	Ho Chi Minh, Vietnam	Data Contributor	GP2
Lam Hoang	Son			University Medical Center Ho Chi Minh City	Ho Chi Minh City, Vietnam	Data Contributor	GP2
Masharip	Atadzhanov			University of Zambia	Lusaka, Zambia	Data Contributor	GP2
