# Supplementary material for: Pathology and Genetics in a Global Cohort of Parkinsonian Disorders
Source: JAMA Neurol. 2026 Jun 8:e261634. Online ahead of print. doi: 10.1001/jamaneurol.2026.1634 (PMC13247843; doi:10.1001/jamaneurol.2026.1634)
Supplement: Supplement 3. — Data Sharing Statement. [file jamaneurol-e261634-s003.pdf]

Data Sharing Statement

Wu. Pathology and Genetics in a Global Cohort of Parkinsonian Disorders. *JAMA Neurol*. Published June 08, 2026. doi:10.1001/jamaneurol.2026.1634

Data
Data available: Yes
Data types: Deidentified participant data
How to access data: Researchers need to sign a data access agreement here: https://amp-pdrd.org/register-for-amp-pd and can then access data on the cloud: https://verily.com/solutions/pre-platform/data-partners/gp2 Includes clinical, pathological and genetic data
When available: beginning date: 12-01-2025

Supporting Documents
Document types: Statistical/analytic code
How to access documents: Data code: https://github.com/GP2code/MD-GAP-GP2-CPC
When available: beginning date: 12-01-2025

Additional Information
Who can access the data: Researchers who are affiliated with an institution with a research proposal approved by AMP-PD
Types of analyses: Any genetic analysis
Mechanisms of data availability: Researchers need to sign a data access agreement with AMP-PD. Once the agreement is signed, they can create a Verily account and access data on the platform.
